# Active vs Traditional Methods of Recruiting Children for a Clinical Trial in Rural Primary Care Clinics: A Cluster-Randomized Clinical Trial

**DOI:** 10.1001/jamanetworkopen.2022.44040

**Published:** 2022-11-29

**Authors:** Paul M. Darden, Ann M. Davis, Jeannette Y. Lee, Milan Bimali, Alan E. Simon, Andrew M. Atz, Crystal S. Lim, Thao-Ly T. Phan, James R. Roberts, Russell J. McCulloh, Lee Pyles, Michelle Shaffer, Jessica N. Snowden

**Affiliations:** 1 Population Health Research, Arkansas Children’s Research Institute, Little Rock; 2 Department of Pediatrics, University of Arkansas for Medical Sciences, Little Rock; 3 Center for Children’s Healthy Lifestyles & Nutrition, Kansas City, Missouri; 4 Department of Pediatrics, University of Kansas Medical Center, Kansas City; 5 Department of Biostatistics, University of Arkansas for Medical Sciences, Little Rock; 6 Environmental influences on Child Health Outcomes Program, National Institutes of Health, Rockville, Maryland; 7 Department of Pediatrics, Medical University of South Carolina, Charleston; 8 Department of Psychiatry and Human Behavior, University of Mississippi Medical Center, Jackson; 9 Nemours Children’s Health and Sidney Kimmel Medical College at Thomas Jefferson University, Wilmington, Delaware; 10 Department of Pediatrics, University of Nebraska Medical Center, Omaha; 11 Department of Pediatrics, University of West Virginia, Morgantown; 12 Department of Pediatrics, University of Arkansas for Medical Sciences, Little Rock

## Abstract

**Importance:**

To our knowledge, there are no published randomized clinical trials of recruitment strategies. Rigorously evaluated successful recruitment strategies for children are needed.

**Objective:**

To evaluate the feasibility of 2 recruitment methods for enrolling rural children through primary care clinics to assess whether either or both methods are sufficiently effective for enrolling participants into a clinical trial of a behavioral telehealth intervention for children with overweight or obesity.

**Design, Setting, and Participants:**

This cluster-randomized clinical trial of 2 recruitment methods was conducted at 4 primary care clinics in 4 separate states. Each clinic used both recruitment methods in random order. Clinic eligibility criteria included at least 40% pediatric patients with Medicaid coverage and at least 100 potential participants. Eligibility criteria for children included a rural home address, age 6 to 11 years, and body mass index at or above the 85th percentile. Recruitment began February 3, 2020, and randomization of participants occurred on August 17, 2020. Data were analyzed from October 3, 2021, to April 21, 2022.

**Interventions:**

Two recruitment methods were assessed: the active method, for which a list of potential participants seen within the past year at each clinic was generated through the electronic health record and consecutively approached by research staff based on visit date to the clinic, and the traditional method, for which recruitment included posters, flyers, social media, and press release. Clinics were randomized to the order in which the 2 methods were implemented in 4-week periods, followed by a 4-week catch-up period using the method found most effective in previous periods.

**Main Outcomes and Measures:**

For each recruitment method, the number and proportion of randomized children among those who were approached was calculated.

**Results:**

A total of 104 participants were randomized (58 girls [55.8%]; mean age, 9.3 [95% CI, 9.0-9.6] years). Using the active method, 535 child-parent dyads were approached and 99 (18.5% [95% CI, 15.3%-22.1%]) were randomized. Using the traditional method, 23 caregivers expressed interest, and 5 (21.7% [95% CI, 7.5%-43.7%]) were randomized. All sites reached full enrollment using the active method and no sites achieved full enrollment using the traditional method. Mean time to full enrollment was 26.3 (range, 21.0-31.0) days.

**Conclusions and Relevance:**

This study supports the use of the active approach with local primary care clinics to recruit children with overweight and obesity from rural communities into clinical trials.

**Trial Registration:**

ClinicalTrials.gov Identifier: NCT04142034

## Key Points

**Question:**

Is active or traditional recruitment sufficient for recruiting children from rural communities into a group behavioral telehealth intervention for overweight and obesity?

**Findings:**

In this cluster-randomized clinical trial testing recruitment methods in 4 clinics in 4 states, recruiting eligible participants from a list of recent visits to the clinic (active recruitment) resulted in a substantially greater number of randomized participants than traditional recruitment such as posters and advertisement (99 vs 5 randomized participants).

**Meaning:**

The active recruitment approach is an effective method for recruitment into clinical trials in primary care clinics that care for rural children.

## Teaser

This cluster-randomized clinical trial evaluates 2 methods of recruitment among families of children with overweight and obesity from rural communities for a future effectiveness study.

## Introduction

Randomized clinical trials (RCTs) are the gold standard for evaluating the efficacy and effectiveness of new medical treatments.^1^ Research indicates several challenges to conducting these trials, and the most common threat to completing an RCT is recruitment. A recent review^2^ concluded that 25% of RCTs are not completed owing to lack of recruitment and that these discontinued studies are more likely to remain unpublished. Investigations of recruitment in clinical trials show that slow recruitment often delays study completion (in ≤53% of studies) or the study is closed without achieving half of planned recruitment (24% of studies), with an estimated 31% to 34% of trials actually meeting their initial recruitment targets.^3^ These delays can be costly and can also limit the ability of the trial to test proposed hypotheses.

Prior studies on clinical trial procedures have not demonstrated conclusive or generalizable solutions to poor recruitment. One systematic review^4^ indicated that monetary incentives, repeated invitations to participate, and increased information on the consent form may be helpful to increase recruitment rates, but the findings were not conclusive. Another review^5,6^ indicated that telephone reminders to nonresponders, opt-out procedures, and open study designs may be helpful, but the authors noted several disadvantages to these procedures as well, including threats to trial validity. A project that evaluated recruitment into a community nutritional trial^7^ found that use of institutional email lists was most effective compared with Facebook and print advertisement. Some studies have tested specific recruitment methods, but these have been for recruitment into hypothetical trials, which are difficult to generalize to actual RCTs.^3^ A 2018 Cochrane review by Treweek et al^8^ found no RCTs that published pretrial planning of recruitment strategies and no RCTs of recruitment strategies.

Beyond general challenges to recruitment, important groups are largely underrepresented in RCTs. Specifically, individuals who are members of racial and ethnic minority groups and those living in rural communities are less likely to be enrolled in trials.^9,10^ If rural and underserved populations are not represented in clinical trials, the findings may not be generalizable to these groups. Barriers and facilitators to the participation of children from rural communities in clinical trials are understudied^11^ and include mistrust of research and the health care system, perceived risks, cost, and transportation issues.^12^ Residents of rural communities may be more concerned than those from nonrural communities about the time commitment and more influenced by possible results benefiting affected family members.^13^ Engagement of local and trusted community resources and primary care clinics as research sites can increase participation among rural populations,^14^ and rural clinics may be more likely to engage in practice-based network research than nonrural clinics.^15^ Compounding these recruitment challenges for rural and underserved communities, there are also substantial barriers to recruiting participants into behavioral obesity trials, including logistical challenges, cost, weight stigma, and lack of interest from patients or health care practitioners.^16^ The use of a telehealth intervention, as in the present study, could address issues related to transportation and the time needed to participate in rurally focused interventions.

The purpose of the present project was to implement a cluster RCT designed to evaluate 2 methods of recruitment among families of children with overweight and obesity from rural communities for a future fully powered effectiveness study. Specifically, we evaluated active recruitment (approaching potential participants in a standardized sequential manner based on visit date to the clinic) and traditional recruitment (posters, flyers, social media, websites, and press release) using a cluster-randomized design at the clinic level. We wanted to determine whether either or both recruitment methods would achieve a target recruitment rate of at least 20% and if there were differences between the recruitment methods with respect to recruitment rate or recruitment duration.

## Methods

**Figure 1.**
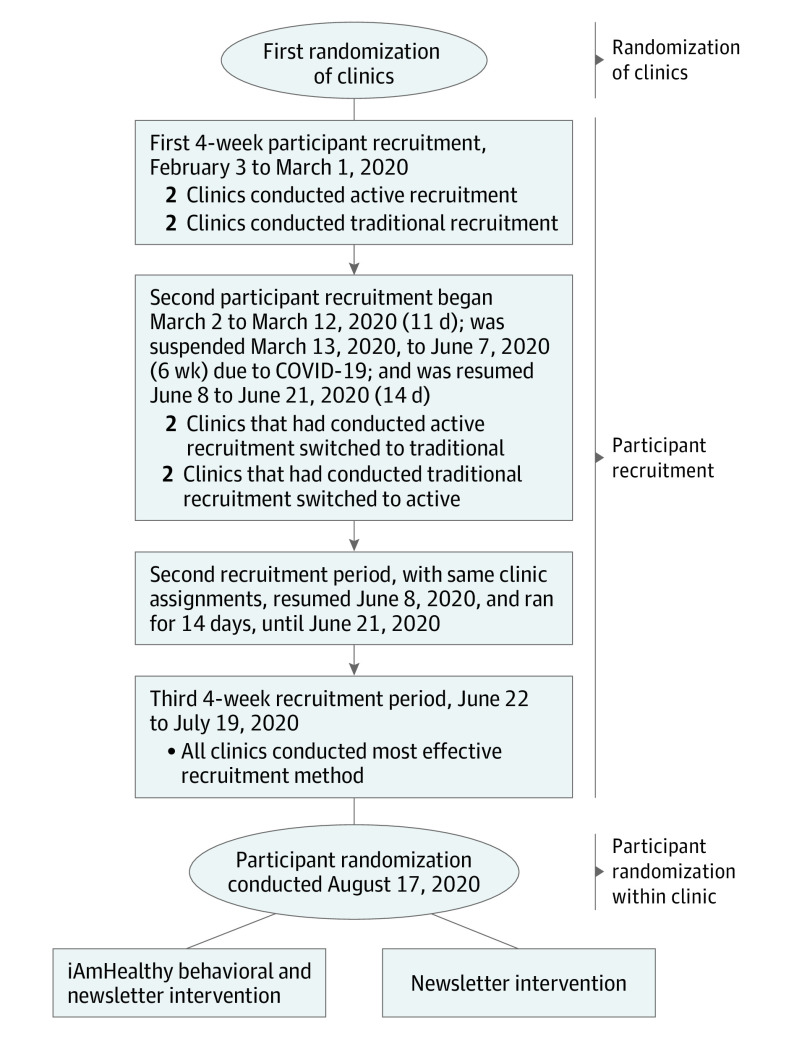
Study Design and Timeline

This cluster RCT was performed in 4 clinics (clusters) treating children from rural communities in 4 states that are members of the Environmental Influences on Child Health Outcomes Institutional Development Award State Pediatric Clinical Trials Network (ISPCTN).^17^ The trial protocol is available in Supplement 1. The study design has been published^18^ and is described in brief below. Recruitment began February 3, 2020, with randomization of participants on August 17, 2020 (Figure 1). Children with overweight or obesity and their parents or caregivers were recruited into an RCT of a telehealth behavioral management intervention plus a newsletter vs a newsletter control group. The behavioral intervention included virtual group and individual sessions for 6 months attended by the caregivers and children. The University of Arkansas for Medical Sciences served as the central Institutional Review Board for all sites and approved the study. For the cluster RCT, clinics did not provide written informed consent. For the individual RCT, study personnel obtained written informed consent from the caregiver and, when appropriate, assent from child participants. Clinic personnel did not participate in any research activity. The study followed the Consolidated Standards of Reporting Trials (CONSORT) reporting guideline.

### Patient and Clinic Eligibility

Each site partnered with a local primary care clinic that met clinic inclusion criteria and was willing to allow access to their patients. Clinic eligibility criteria are outlined in Table 1 and have been published previously.^19^

**Table 1.** Inclusion and Exclusion Criteria for the Study for Child and Clinic

Criterion	Definition
**Inclusion criteria for the child**	
Rural residence	Child lives in a zip code with an RUCA code ≥4^a^
Aged 6-11 y at the time of consent	Narrow age range to decrease developmental variability
BMI percentile ≥85%	Criteria for the definition of overweight and obese
English speaking	Child and primary caregiver must speak English
**Inclusion criteria for the clinic**	
Previous collaboration with the ISPCTN site	A previous project between ISPCTN site and participating clinic
40% Medicaid enrollment	40% of the pediatric patient pool of the clinic had Medicaid coverage
Seen 100 potential participants in the past year	Clinic has seen >100 children meeting inclusion criteria in the past year to support recruitment
Uses an EMR	Clinic uses an EMR
Resources to support the conduct of the project	Space for consenting and height and weight measurement
**Exclusion criteria for the child**	
Physical limitation or injury	Children who cannot be physically active
Known significant medical issue	Significant medical issue (such as cancer) known to the clinic that could affect protocol adherence
Developmental delay or cognitive impairment	Primary caregivers and/or children with a known developmental delay or other cognitive impairment, which may impede participation in groups, are excluded
Enrolled in a weight-loss trial	To avoid confounding
Sibling already consented	Siblings allowed to attend the intervention but not be a participant owing to measurement bias

Abbreviations: BMI, body mass index; EMR, electronic medical record; ISPCTN, Institutional Development Award State Pediatric Clinical Trials Network; RUCA, Rural Urban Commuting Area.

^a^ Defined using US Department of Agriculture RUCA ZIP Code Crosswalk version 3.1 from the University of North Dakota.^19^ RUCA codes 4 or greater are considered rural.

### Recruitment Methods

#### Active

The active recruitment method used lists of eligible patients generated from each clinic’s electronic health record. All clinics were able to generate lists of children meeting all eligibility criteria except rurality. Each site provided a frequency list of zip codes that were categorized as rural and urban based on Rural-Urban Commuting Area code. The active recruitment method consisted of retrospective and prospective approaches. For the retrospective approach, a list of eligible potential participants was generated based on patients seen in the last year. A letter of introduction to the study on clinic or practice letterhead and signed by the primary care practitioners was mailed to each potential participant to briefly explain the study. It included a telephone number to call if the family was not interested and did not wish to be contacted for recruitment by the study team (ie, opt out). Two weeks after letters were sent (ie, to allow families time to opt out), research staff began contacting potential study participants via the telephone number listed in the clinic electronic health record (telephone call or text). Staff started with those who had had the most recent visits and worked backward. This approach was considered retrospective active recruitment. For the prospective approach, research staff recruited from a list of potential participants generated from upcoming appointments.

For the active method, *approached* was defined as potential participants engaging in reciprocal interactions with any member of the study team. This could have been a telephone call and conversation with the caregiver or a text to the caregiver’s telephone number with a reply.

#### Traditional

The traditional recruitment method consisted of a variety of techniques commonly used for clinical trial recruitment, including placing posters throughout the clinic and in local community centers, flyers handed out within the clinic, advertisements on social media (ie, Facebook) and on research and patient education websites, press releases, and recommendations by staff and/or health care practitioners to consider participation. Each site and clinic chose the traditional methods used. For this method, *approached* was defined as caregivers who expressed interest in the study and interacted with a member of the study team.

### Procedures

**Figure 2.**
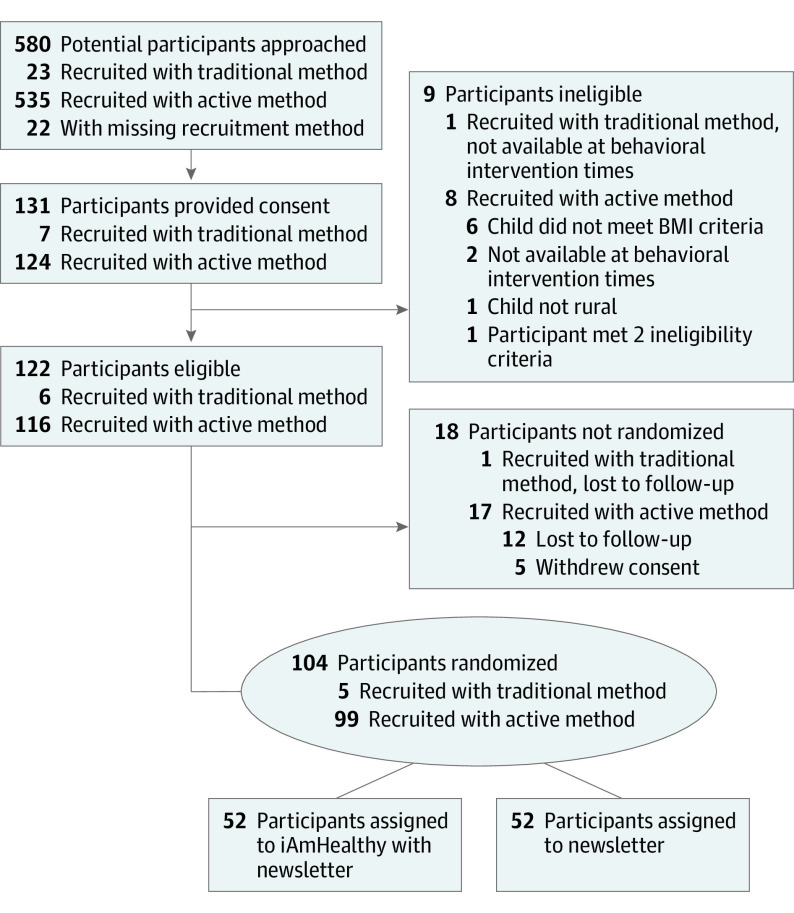
Study Flow Diagram BMI indicates body mass index.

Randomization was unblinded and, at the clinic level, conducted by the ISPCTN Data Coordinating and Operations Center and constrained such that 2 clinics were in each recruitment method at a time (Figure 1). Clinics were randomized to the recruitment method order after clinic eligibility was confirmed. Recruitment proceeded for three 4-week periods, 2 randomized periods, and a catch-up period. Two sites began with the active method for recruitment period 1 and then switched to the traditional method for recruitment period 2. The other 2 sites began with the traditional method and then switched to the active method. During the catch-up period, each site could choose the method that worked best to complete their recruitment and meet enrollment targets (Figure 1). Recruitment was interrupted by COVID-19–related institutional restrictions on recruitment. During the recruitment suspension due to COVID-19, recruitment procedures shifted to being fully virtual, and the catch-up period was increased from 2 to 4 weeks. To describe the characteristics of the participants, race and ethnicity and sex were self-reported and recorded at the initial interview. More details on the study methods are available elsewhere.^18^ Data reported herein were collected by research-trained coordinators at each site. Each site was asked to recruit a minimum of 16 and a maximum of 32 participants, with a target of 28 participants during the almost 12-week recruitment period (81 days [Figure 1]) from each participating clinic. The cluster RCT ended with the individual randomization of participants (Figure 1 and Figure 2).

### Planned Statistical Analysis

Because the purpose of this study was to assess whether either or both of these recruitment methods were appropriate to fill a full-scale trial, our a priori specified goal for each recruitment method was to test the null hypothesis that the proportion randomized was 13% against the alternative hypothesis that it was 20% at a significance level of 2-sided *P* < .05 and power of 0.90 using an exact test for proportions. An intraclass correlation coefficient of 0.01 and cluster size of 70 for each recruitment method was used. This required a planned sample size of 560 potential participants approached (280 per arm). The specification of the null hypothesis at 13% randomized was to exclude the alternative hypothesis of 20% randomized, an important expectation for feasibility of the recruitment methods. For each recruitment method and period, the proportion of approached individuals who were randomized into the study was estimated with the binomial proportion and its exact 95% CI. The general mixed model with a binomial distribution and logit link was used to determine whether the randomization proportion differed by period or recruitment method using clinic as a random effect.

Time to full recruitment for each recruitment option for each clinic was defined as the number of days from the start of that recruitment method to consent of the 14th participant randomized by that method (one-half of full recruitment for each site for the trial). However, no clinic reached 14 participants using the traditional option, so no statistical comparison of time to full recruitment was performed between the traditional and active methods. All analyses used SAS, version 9.4 (SAS Institute Inc). Data were analyzed from October 3, 2021, to April 21, 2022.

## Results

Opt-out letters were sent to 1482 potential participants, and 13 (0.9%) requested no further contact related to the study (ie, opted out). A total of 580 potential participants were approached for recruitment, 535 using the active method, 23 using the traditional method, and 22 for whom the method of recruitment could not be determined (Figure 2). All 4 randomized clinics completed the project, participated in both recruitment methods, had participants randomized into the study, and are included in all analyses.

The study team approached nearly twice as many potential participants as expected with the active method (535 vs the expected 280) and less than one-tenth of the expected participants using the traditional method (23 vs the expected 280). All sites spent twice as much recruitment time in the active method (randomized period plus catch-up) as in the traditional method of recruitment (randomized period only) (Figure 1). In the active method, almost all potential participants were approached using the retrospective active method. The prospective active method (recruitment of patients with upcoming appointments) was not widely used and would have been difficult in the second half of the study owing to COVID-19 suppressing typical clinic volumes. The number of consented participants comprised 124 using the active method and 7 using the traditional method, respectively (Figure 2). Twenty-seven consented participants did not proceed to randomization: 9 were ineligible, 5 caregivers withdrew consent from the active approach, and 13 were lost to follow-up. Figure 2 provides reasons for and sequence of ineligibility and withdrawal.

Of 580 potential participants approached, 131 consented and 104 were randomized. Complete demographic data available for randomized participants are given in Table 2. Of the 131 participants who consented, 124 consented using the active recruitment method, compared with only 7 using the traditional recruitment method (Table 3). Of the 124 participants who consented using the active method, 12 consented using the prospective approach whereas 112 consented using the retrospective approach. Overall, 18.6% of the 558 potential participants contacted were randomized: 99 (18.5% [95% CI, 15.3%-22.1%]) for the active method and 5 (21.7% [95% CI, 7.5%-43.7%]) for the traditional method. The proportion of participants approached who were randomized varied by clinic from 22 of 165 (13.3%) to 30 of 114 (26.3%) (Table 3). The difference in recruitment between methods (active minus traditional methods) was −3.2% (95% CI, −20.4 to 13.94). The null hypothesis that the recruitment rate was 13% was rejected in favor of the alternative that it is 20% for the active method (*P* < .001) but not the traditional method (*P* = .11). Thus, the active method demonstrated a recruitment rate that would be acceptable for a full-scale trial, whereas the traditional method did not.

**Table 2.** Sex and Race and Ethnicity of Randomized Child Participants by Treatment Group, Clinic, and Recruitment Method

Characteristic	Randomized participants^a^						
	By group			By clinic^b^			
	iAH plus newsletter intervention (n = 52)	Newsletter intervention (n = 52)	All (n = 104)	1 (n = 24)^c^	2 (n = 28)^c^	3 (n = 30)^d^	4 (n = 22)^e^
Sex							
Boys	20 (38.5)	26 (50.0)	46 (44.2)	8 (33.3)	11 (39.3)	15 (50.0)	12 (54.5)
Girls	32 (61.5)	26 (50.0)	58 (55.8)	16 (66.7)	17 (60.7)	15 (50.0)	10 (45.5)
Unknown	0	0	0	0	0	0	0
Age, mean (95% CI), y	9.4 (9.0-9.9)	9.2 (8.7-9.6)	9.3 (9.0-9.6)	9.1 (8.4-9.8)	9.3 (8.7-9.9)	9.2 (8.6-9.8)	9.5 (8.7-10.3)
Race							
Black	16 (30.8)	8 (15.4)	24 (23.1)	8 (33.3)	0	16 (55.3)	0
White	33 (63.5)	35 (67.3)	68 (65.4)	10 (41.7)	25 (89.3)	13 (43.3)	20 (90.9)
Multiple or other^f^	3 (5.8)	9 (17.3)	12 (11.5)	6 (25.0)	3 (10.7)	1 (3.3)	2 (9.1)
Ethnicity							
Hispanic	5 (9.6)	3 (5.8)	8 (7.7)	3 (12.5)	3 (10.7)	2 (6.7)	0
Non-Hispanic	45 (86.5)	49 (94.2)	94 (90.4)	20 (83.3)	25 (89.3)	28 (93.3)	21 (95.5)
Unknown	2 (3.8)	0	2 (1.9)	1 (4.2)	0	0	1 (4.5)
Medicaid							
Yes	30 (57.7)	21 (40.4)	51 (49.0)	15 (62.5)	6 (21.4)	23 (76.7)	7 (31.8)
No	22 (42.3)	31 (59.6)	53 (51.0)	9 (37.5)	22 (78.6)	7 (23.3)	15 (68.2)

Abbreviation: iAH, iAmHealthy.

^a^ Unless otherwise indicated, data are expressed as No. (%) of participants.

^b^ Because of small numbers using the traditional method, demographic details are not given by recruitment method within each clinic.

^c^ Includes 0 participants recruited with the traditional method.

^d^ Includes 3 participants recruited with the traditional method.

^e^ Includes 2 participants recruited with the traditional method.

^f^ Includes 11 participants with self-reported multiple options for race and 1 participant identifying as Puerto Rican.

**Table 3.** Patients Approached, Providing Consent, and Randomized and Time to Full Enrollment by Clinic and Overall

Clinic	Recruitment method	No. approached (n = 558)	Provided consent, No. (%) (n = 131)^a^	Time to full recruitment, consented, d^b^	Randomized, No. (%) (n = 104)^a^	Time to full recruitment, randomized, d^b^
1	Traditional	6	1 (16.7)	NA	0	NA
	Active	121	28 (23.1)	21	24 (19.8)	33
	Overall	127	29 (22.8)	NA	24 (18.9)	NA
2	Traditional	2	0	NA	0	NA
	Active	150	34 (22.7)	29	28 (18.7)	35
	Overall	152	34 (22.4)	NA	28 (18.4)	NA
3	Traditional	11	3 (27.3)	NA	3 (27.3)	NA
	Active	103	33 (32.0)	24	27 (26.2)	36
	Overall	114	36 (31.6)	NA	30 (26.3)	NA
4	Traditional	4	3 (75.0)	NA	2 (50.0)	NA
	Active	161	29 (18.0)	31	20 (12.4)	50
	Overall	165	32 (19.4)	NA	22 (13.3)	NA
Overall	Traditional	23	7 (30.4)	NA	5 (21.7)	NA
	Active	535	124 (23.2)	26.3 (21.0-31.0)^c^	99 (18.5)	38.5 (33.0-50.0)^c^
	Overall	558	131 (23.5)	NA	104 (18.6)	NA

Abbreviation: NA, not applicable.

^a^ The proportion reported is proportion of approached who were consented or randomized, respectively.

^b^ Calculated as the number of days from the beginning of the active recruitment method for that site to the date of consent of the 14th participant. Time to full enrollment for the traditional method is not shown because no clinic reached full enrollment (ie, 14 randomized participants using that method).

^c^ Indicates mean (range).

The small number of participants randomized through the traditional method precluded reporting of demographics by method owing to privacy concerns. The randomized participants included 58 girls (55.8%) and 46 boys (44.2%) with a mean age of 9.3 years (95% CI, 9.0-9.6). In terms of race and ethnicity, 24 participants (23.1%) were Black, 68 (65.4%) were White, and 12 (11.5%) were of multiple or other races and ethnicities. A dramatic difference in these characteristics was found between clinics. Clinics varied by race from 0 Black participants to 16 of 30 (53.3%) and by Medicaid participation from 21.4% to 76.7% (Table 2).

When we examined the time to full enrollment (Table 3), we saw that although the proportion enrolled by the 2 methods was not different, the times to full enrollment using each method over time were different. All sites were able to reach full recruitment using the active method, whereas no clinic reached full enrollment using the traditional method. The mean time to full enrollment by the active method among those consented was 26.3 (range, 21-31) days. Among clinics, examining their randomized participants, the mean time to full enrollment by clinic was 38.5 (range, 33-50) days. We found that before and after the pause in recruitment for COVID-19, there was little effect on time to full enrollment.

## Discussion

Robust recruitment is critical for clinical trial success. To our knowledge, this is the first study to use a randomized method to test different methods of recruiting participants to an active trial. Furthermore, it involved the recruitment of children living in rural communities, making the study highly innovative and potentially relevant for future trials that aim to recruit children and families from underserved and rural communities. The traditional method of recruitment, commonly used in clinical trials and relying on participant self-identification, resulted in a small number (n = 23) of approached potential participants. This number was far short of our goal of 280 participants, and far short of the 535 approached using the active method. Time to full recruitment varied by clinic, but all clinics successfully recruited using the active method.

The present findings are consistent with those of previous nonrandomized studies of adults. Bjorn et al^20^ examined methods of recruitment for adult participants into 9 trials and found that active approaches attained higher recruitment rates than traditional approaches (active, 17%-26% recruitment rate; traditional, 4%-7% recruitment rate). Interestingly, they also reported that the active approach yielded more representative samples, although representativeness was not tested in the present study. To our knowledge, no previous studies have randomized recruitment methods, but a recent review by Cui et al^21^ reported on recruitment across 43 pediatric obesity studies targeting children in underserved populations. Findings indicate that 64% of studies did not report a recruitment goal, and only 8 reported the duration of the recruitment period. Recruitment rate ranged from 10% to 90% among studies that reported recruitment rate. It is also important to note that only 5 of the studies were conducted in medical clinics; most were conducted in school or daycare settings.^21^ Previous research^16^ has indicated additional challenges around approaching patients during a medical visit for participation in pediatric obesity research, including time restraints of clinicians, lack of interest from health care practitioners, and stigma. Our study avoided this constraint by primarily approaching recently seen potential participants outside the clinic.

Contacting of patients by study staff as opposed to clinic staff can have Health Insurance Portability and Accountability Act implications because protected patient information must be released from the clinic team to the study team. Our use of an opt-out letter (ie, a letter that allowed patients a way to not be contacted if they expressed this to their health care practitioner in a specific period) was sufficient for the institutional review board to allow this release of information to the study team.

### Limitations

Several factors could limit the generalizability of these findings. First, some caregivers reported that they did not have time for the demands of the study, and therefore did not enroll or withdrew from the project, potentially limiting generalizability to studies with different demands. Second, the present study only included 4 clinics serving children from rural communities in 4 states, which could limit generalizability to nonrural clinics or clinics in other states. Interestingly, all 4 rural clinics were able to produce a recruitment list with relative ease, but these results may not generalize to clinics that are not able to do so. Third, the participating clinics had a previous relationship with the academic site so recruitment results may have differed in the absence of a prior relationship. Also, the recruitment period for the present study was short. It could be that these methods would perform differently during a longer recruitment period. Additionally, the timing of this study during COVID-19 may have differentially affected each recruitment method. Although three-fourths of the randomized recruitment periods and half of the total recruitment period occurred before the COVID-19 shutdown (Figure 1), clinic visit patterns may well have been affected throughout recruitment and could have affected the performance of the recruitment methods.

## Conclusions

In this cluster RCT of recruitment methods, the active method resulted in sufficient recruitment to support a full-scale RCT of pediatric obesity treatment in rural children. Future research may determine whether these findings apply to other types of pediatric trials and in nonrural areas.

## Notes

Supplement 1.
Trial Protocol

Supplement 2.
Data Sharing Statement
