# Supplementary material for: Active vs Traditional Methods of Recruiting Children for a Clinical Trial in Rural Primary Care Clinics: A Cluster-Randomized Clinical Trial
Source: JAMA Netw Open. 2022 Nov 29;5(11):e2244040. doi: 10.1001/jamanetworkopen.2022.44040 (PMC9709648; doi:10.1001/jamanetworkopen.2022.44040)
Supplement: Supplement 1. — Trial Protocol [file jamanetwopen-e2244040-s001.pdf]

iAmHealthy

Feasibility Trial of the iAMHealthy Intervention for Healthy Weight in Rural Children Recruited From Primary Care Clinics

NCT04142034

Protocol (March 4, 2021)

Feasibility Trial of the iAmHealthy Intervention for Healthy Weight in Rural Children Recruited from Primary Care Clinics

UAMS IRB (cIRB) Protocol Number: 249932

Version Number: 13

04 March 2021

Operational Principal Investigator:

Jessica Snowden, MD (University of Arkansas for Medical Sciences)

Principal Investigators:

Ann Davis, PhD (University of Kansas Medical Center)

Paul Darden, MD (University of Oklahoma Medical Center)

Funded by: National Institutes of Health (NIH)

Summary of Changes from Version 01 to Version 02:

(Primarily responses to pre-review contingencies)

Affected Section(s)	Summary of Revisions Made (01 to 02)	Rationale
Compliance Statement	Deleted references to FDA regulations 21 CRF Parts 50, 56, 312, and 812	This is not an FDA-regulated study.
5.5 Recruitment of Clinics	Add the following to the last bullet of the section: “The clinic nurse will only provide contact information (of potential participants) to the ISPCTN site personnel and provide IRB-approved recruitment materials to potential participants. The clinic nurse will also ask potential participants if it is okay for the ISPCTN site personnel to contact the potential participants.”	Clarification requested by UAMS IRB. Clarification concerns role of clinic nurse and whether or not clinics are considered engaged in research.
5.5 Retention Plans Table	Change column header FROM “newsletter-only” to “retention contact” and deleted “after each weekly group session” and “after each monthly session” from tables.	Simple corrections.
7.1 Participant Consent/Screening and Baseline Visits	To first “consent” paragraph, added that the research-trained designee will be from the ISPCTN site<br>To second “consent” paragraph, added “The link will be sent to the primary caregiver by a member of the ISPCTN awardee site or a member of the ISPCTN DCOC.”	Clarifications requested by UAMS IRB. Clarifications concern whether or not clinics are considered engaged in research.
7.1 Participant Consent/Screening and Baseline Visits	To “randomization” paragraph, added that the backup site staff member will be an ISPCTN site staff member.	Clarifications requested by UAMS IRB. Clarifications concern whether or not clinics are considered engaged in research.
7.2 Administration and Feasibility Study Intervention	Added that the backup site staff member will be an ISPCTN site staff member.	Clarification requested by UAMS IRB. Clarification concerns whether or not clinics are considered engaged in research.

Summary of Changes from Version 02 to Version 03:

(Responses to expedited review contingencies + additional corrections)

Affected Section(s)	Summary of Revisions Made (02 to 03)	Rationale
Glossary of terms	Added "Backup Assessor" and definition.	Requested by IRB.
4.3, subsection "Justification for Delivering …"	Deleted "In addition we will lock the tablets and only allow participants to use them for intervention-related materials."	This is not part of the tablet preparation process for this study.
5.5, subsection "Recruitment of clinics"	Added:<br>(a) "The clinic nurse will not conduct study procedures such as data collection."<br>(b) The clinic nurse would, "upon being instructed by the unblinded coordinator, distribute tablets to those participants assigned to the tablet group."	(a) Requested change by IRB.<br>(b) Procedural correction.
5.5, subsection "retention plans"	Added paragraph on compensation for participants.	Requested change by IRB.
5.5 subsection "retention plans"	Revised content of tables for weeks 1-12 and for weeks 13-24.<br>Changes include:<br>- Deletion of requirement to send "kid questions."<br>- Changed "texting/emailing" questions for caregivers to asking question during phone call about AEs/SAEs.<br>- Added footnotes regarding schedule for AE/SAE calls.<br>- Deletion of requirement to send exercise tips.<br>- Deletion of requirement to send recipes.<br>- Deletion of requirement to send summary of materials covered/presented to date.	Simplification and procedural changes required because newsletter-only group is not required to have electronic devices.
5.5, subsection "Tracking Retention Plans"	• Changed who will be sending and tracking. Changed from "DCOC" to "unblinded coordinator."<br>• Deleted paragraph about texting sites and paragraph about sending branded emails.	• Method was changed for who will be tracking.<br>• Texting and branded emails will not be sent for retention because newsletter-only participants are not

Affected Section(s)	Summary of Revisions Made (02 to 03)	Rationale
		required to have electronic devices.
7.2, Subsection “Acquisition and Preparation of Tablets.	Deleted statement that tablets will be locked from non-study-related apps and programs.	The tablet preparation does not include this process.
8.1, Discontinuation of Study Intervention	Deleted statement requiring collection of data if trial is discontinued.	Response to IRB contingencies. Statement should not have been in protocol.
9.2, subsection “Time period and frequency for event assessment and follow-up	Added schedule for AE/SAE calls to participant and changed who the DCOC personnel will contact for SAE follow-up (changed from “participant” to “unblinded coordinator”).	Clarifications and simple correction.
11.1, subsection “Confidentiality and Privacy”	Noted that “other oversite offices” may have access to participants’ information.	Requested change by IRB.

Summary of Changes from Version 03 to Version 04:

Affected Section(s)	Summary of Revisions Made (03 to 04)	Rationale
Header and footer	Formatting changes	Font consistent with protocol text
Throughout protocol	ISPCTN to ECHO ISPCTN	NIH-requested branding change
Table of Abbreviations	Updated ISPCTN definition to include ECHO	NIH-requested branding change
1.1 Synopsis	Changed Objectives to coincide with changes in Section 10.1.	DSMB requested change
3 Objectives and Endpoints	Objective 1: updated to coincide with changes in Section 10.1.	DSMB requested change
5.5 subsection “Recruitment of Clinics”	5th bullet: Updated to correct what clinic nurse will do	Protocol correction
5.5 subsection “Retention Plans”	Deleted: Immediately in timeline for first retention card.<br>End of Week 12 retention contact<br>Participant/caregiver thank you with diploma attachment<br>Added: Text similar to what will be included on each card	Protocol correction

Affected Section(s)	Summary of Revisions Made (03 to 04)	Rationale
7.1 subsection "Participant Consent/Screening…"	Deleted: The link will be sent by a member of the ISPCTN awardee site or a member of the ISPCTN DCOC<br><br>Added: If the primary caregiver does not want to complete the iAmHealthy Demographics Form during the consent/assent visit	Protocol correction
7.2, subsection, Acquisition and Preparation of Tablets"	Corrected process for tablet distribution	Protocol correction
7.3 Measures to Minimize Bias…	Changed number of child/caregiver pairs sites can randomize from 28 to 32<br><br>Deleted the system will bind site coordinator from other data collection time points	Protocol correction
8.1 Discontinuation of Study Intervention	Added: as specified in the MOP	Protocol clarification
8.2 Participant Discontinuation…	Changed number sites can consent up to from 28 to 32	Protocol clarification
9.1, subsection "Secondary Outcome Measures"	Deleted: Harpenden Hotain Stadiometer, model 603 (Holtain, Crymych, UK), Bluetooth SECA digital scale, model 703 (SECA, Hamburg, Germany), and the sentence about Model 703 accuracy.<br>Add: Detecto Portable Height Rod (PHR) (Detecto, Webb City, MO)<br>Seca 813 High Capacity Digital Flat Scale for Individual Patient Use (Seca North America, Chino, CA)	Protocol update
9.1, subsection "Secondary Outcome Measures"	Deleted: we and monthly<br>Halt screening/baseline<br>Added: The ECHO ISPCTN site coordinator<br>Weekly (reliability assessment)<br>Starting (to consent process)	Simplification of process

Affected Section(s)	Summary of Revisions Made (03 to 04)	Rationale
9.2, subsection “Time period and Frequency…	Added “contact the ECHO ISPCTN unblinded coordinator	Update protocol
9.2, subsection “Reporting of Pregnancy”	Added: If a participant reports pregnancy	Clarify process
10.1, subsection “Primary Objective”	Added: Hypothesis 2	DSMB request
10.4, subsection “Analysis of Primary Endpoints”	Add: Second paragraph to Objective 1B	DSMB request
11.1, subsection “Data Collection and Management Responsibilities”	Deleted: included in the MOP<br>A 21 CRF Part 11 compliant EDC system<br>Added: an EDC system that complies with HIPAA regulations	Protocol correction
11.1, subsection “Key Roles and Study Governance”	Basic formatting changes for consistency<br>Updated address of Operational PI<br>Add Medical Monitor	Address change to reflect recent move

Summary of Changes from Version 04 to Version 05:

Affected Section(s)	Summary of Revisions Made (04 to 05)	Rationale
Throughout protocol	Two-week run-up changed to four-week run-up	Procedural correction
7.2, subsections Acquisition and Preparation of Tablets; Tablet Delivery and Storage	Changed process for distributing and returning tablets	Procedural correction
7.3 Measures to Minimize Bias: Randomization and Blinding	Clarified randomization process	Procedural clarification
9.1, subsection Secondary Outcome Measures	Clarified inter-rater reliability process	Procedural clarification
Glossary of Terms	Correct alphabetized list	Protocol correction

Summary of Key Changes from Version 05 to Version 06

Affected Section(s)	Summary of Revisions Made (05 -06)	Rationale
1.3 SOA	Add asterisks to Baseline and Height and Weight rows. Add footnote that consent and baseline activities can occur during the same visit.<br><br>Merge cells in Demographics row to show that the demographics questionnaire can be completed anytime during consent/screening baseline (Word will not track this change)<br><br>Add "X" in Study Personnel Time Assessment Row under consent/screening column and baseline column. Deleted "X" under Weeks 25-28. This assessment will be completed during consent/screening and baseline and not during post-intervention.<br><br>Study Personnel Satisfaction Survey: Merged cells in Day 0 and Week 1-4 and added "X" to show that this survey will be done during this time and during post-intervention.	Procedural change
Section 5.5, Subsection Recruitment Goals and Monitoring	3rd paragraph: added the following text:<br><br>If sites successfully recruit 16 participants in recruitment month two, sites can continue recruiting in the same recruitment option up to 32 participants without waiting for the two-week recruitment extension period. If sites are not successful in recruiting 16 participants, sites must continue recruiting in the recruitment option until 16 participants are recruited or the recruitment option ends. During the two-week recruitment extension, all sites can recruit participants using the recruitment option in which the site was most successful, up to the cap of 32 participants/site.	Procedural change to allow sites to continue recruiting once they reach the 16 participants without waiting for recruitment period extension.
Section 5.5, Subsection Retention Plans	Corrected sentence structure<br><br>Deleted "with message outlining next steps" and substituted "card" for "information packet"	Increase readability and comprehension; correct sentence structure<br><br>Procedure change

Affected Section(s)	Summary of Revisions Made (05 -06)	Rationale
Section 5.5, Subsection Tracking Retention Plans	1st Paragraph<br><br>Deleted “and touchpoint information packets”<br><br>Deleted “ECHO ISPCTN site coordinator will request that the”<br><br>Substituted “randomization and intervention” for “the results of the participant screening”<br><br>Deleted last sentence describing contents of information packet and packet tracking<br><br>2nd Paragraph<br><br>Deleted “which touch point card was mailed”<br><br>Deleted “was mailed” and added “track”<br><br>Deleted “and track” to sentence about participant responses to touch-point cards	Procedure change
7.1 Screening and Consent	Deleted “we” and added “the ECHO ISPCTN site coordinator and/or site investigator”	Clarify protocol
7.1 Screening and Consent, Subsection Participant Consent/Screening and Baseline Visits	Added “screening/baseline” to the sentence about when the demographics form can be completed.<br><br>Deleted “assent”	Procedure change
7.3 Measures to Minimize Bias: Randomization and Blinding	Added “consent/screening” to the sentence about when the site coordinators can take height and weight measurements.	Procedure change
7.4 Study Intervention Compliance	Change the amount of sessions that the supervising psychologists will assess for reliability.<br><br>From 25% of selected sessions to 25% of all sessions for primary assessor and 50% of these sessions for the second assessor.	Protocol correction

Affected Section(s)	Summary of Revisions Made (05 -06)	Rationale
9.1 Efficacy Assessment, Subsection Secondary Outcome Measures	Deleted "we." Added "ECHO ISPCTN site coordinators and/or site investigators."<br><br>Deleted "calibrate." Added, "conduct an accuracy check on equipment."	Protocol clarification
9.1 Efficacy Assessment, Subsection Other Measures; Demographics	Added "during consent/screening/baseline"	Procedure change
9.1 Efficacy Assessment, Subsection Other Measures; Study Personnel Time Assessment	Added "During the recruitment phase the DCOC will weekly send a time assessment survey"	Procedure change and clarification of process
9.1 Efficacy Assessment, Subsection Other Measures; Study Personnel Satisfaction Survey	Added, "The DCOC will ask all study personnel to complete a brief satisfaction survey shortly after the recruitment period ends and after the final measurements are completed."	Procedure change
9.1 Efficacy Assessment, Subsection Other Measures, Time Period and Frequency for Event Assessment and Follow-up	Deleted "by monthly asking the participant about the severe AE"<br><br>Added "inquire/follow severe AEs via monthly participant phone calls" to unblinded coordinators responsibilities	Procedure change
Throughout	Corrected minor typographical errors.	Simple corrections.

Summary of Key Changes from Version 06 to Version 07

Affected Section(s)	Summary of Revisions Made (06 to 07)	Rationale
1.2 Schema	Updated to show new timeline	Reflect changes due to enrollment halt because of pandemic

Affected Section(s)	Summary of Revisions Made (06 to 07)	Rationale
1.3 Schedule of Activities	Added SOA table 2 to show new timeline and activities because of enrollment pause. In both SOA tables merged columns to show individual sessions occur throughout intervention period for iAmHealthy behavioral intervention participants	Illustrate changes in timeline because of enrollment halt and to show that individual sessions occur throughout intervention period
2.1 Enrollment Halt	Added explanation for enrollment halt	Explanation of enrollment halt because of pandemic
5.5, subsection Recruitment Option 1: Consecutive	Added "If the clinic staff can produce a list from electronic medical records that shows the BMI percentages of potential participants, this back and forth procedure is not required."	Flexibility in procedures
5.5, subsection Recruitment Goals and Monitoring	Changed recruitment extension window from 2 to 4 weeks	Increase recruitment time because of enrollment halt
5.5, subsection Stopping Rule	Changed recruitment time from 2.5 months to 12 weeks	Consistency with recruitment time extension
5.5 subsection Retention Plans, Compensation	Clarified when participants will receive compensation. Increased ending compensation to $100<br><br>Added that unblinded coordinators will make monthly retention calls	Clarify procedure; increase participant compensation because of enrollment halt<br><br>Clarified purpose of unblinded coordinators calls.
6 Training	Added that the DCOC will train coordinators and study staff on teleconsent informed consent process and documentation and in remote height and weight procedures<br><br>Clarified procedure for behavioral intervention team reporting severe AEs and SAEs to unblinded coordinator	Additional training needed for virtual study procedures<br><br>Consistency with rest of protocol
6.1 Height and Weight Measurement Training	Added section to describe how blinded ECHO ISPCTN coordinators will practice taking virtual height and weight measurements. Moved interrater reliability description from section 9.1, subsection Secondary Outcome measures to section 6.1	Better organize information and have all training information in training section

Affected Section(s)	Summary of Revisions Made (06 to 07)	Rationale
Throughout	Updated heading numbers. Corrected typos or minor errors, and changed six-months to post-intervention.	Updates and clarity
7.1 Consent, Screening, Baseline, and Post-Intervention	Changed subsection Participant Consent/Screening and Baseline Visits to Consent/Assent and Other Informational Documents Provided to Participants<br><br>Moved text that was in section 11.1 to section 7.1<br><br>Added that ECHO ISPCTN coordinators can conduct the consent/assent process either in person or by teleconsent.<br><br>Added how participants indicated assent/consent.<br><br>Added instructions for how the consent process will be conducted. Added that the clinic nurse can give potential participants study information via email, fax, or in person.<br><br>Changed how participants will receive a signed copy of consent form.	Protocol clarification<br><br>Move all information and procedure description about consent to same section of protocol for better organization.<br><br>Added teleconsent consent/assent, how participants can indicate consent/assent, and consent and assent process to allow study procedures to continue during quarantine.<br><br>Added methods for clinic nurse to give potential participants information so clinic patients can receive study information during quarantine.

Affected Section(s)	Summary of Revisions Made (06 to 07)	Rationale
Section 7.1 Consent, Screening, Baseline, and Post-Intervention	Added Screening subsection. Updated screening procedures for virtual screening<br><br>Updated Baseline subsection to Baseline Measurements subsection and clarified baseline procedures and timing<br><br>Added Post-Intervention Measurements Subsection. For clarity, changed 6-month measurements to post-intervention measurements. Added that post-intervention remote height and weight measurements will be taken in parallel with in-clinic height and weight measurements. Moved text from section 9.1, subsection Secondary Outcome Measures.	Process update to allow enrollment and screening to continue during quarantine.<br><br>Because of enrollment halt, all baseline measurements will occur after enrollment/recruitment ends.<br><br>Clarify when measurements will happen and procedure update. Moved text for better organization and for measurement procedures to be in the same place.
8.1 Discontinuation of Study Intervention	Changed when the study will track and record safety events; changed relevant to significant	Consistency with SOA and consistency within protocol
8.2 Participant Discontinuation/Withdrawal from the Study	1st paragraph, 3rd bullet – clarified severe AE. Updated when lost to follow up procedures would begin to account for virtual study procedures	Clarification and change in study procedures
9.1 subsection Objective 1 – Participant Recruitment Rate	Changed recruitment period for each option from one month to four weeks	Clarification of recruitment period
9.1 subsection Other Measures	Updated how these measures will be conducted or distributed<br><br>Demographics form – updated when the form has to be completed (by randomization)	Update procedure to allow study to resume as virtual study and that some procedures may take longer because of virtual study.
9.2 subsection Definition of Adverse Events	Updated definition of adverse event to match DCOC standard operating procedure	Consistency with DCOC SOP

Affected Section(s)	Summary of Revisions Made (06 to 07)	Rationale
9.2 subsection Time Period and Frequency for Event Assessment and Follow-Up	Updated procedure for interventionists if suspect severe AE or SAE<br><br>Add when the unblinded coordinators will start making safety calls<br><br>Added that the ECHO ISPCTN coordinator will track SAEs and update how long unblinded coordinators will track SAEs	Clarify and update procedure<br><br>Text consistent with SOA<br><br>Procedure update and clarification
9.3 subsection Definition of Unanticipated Problems	Updated definition to match DCOC standard operating procedure. Change in definition caused a change in reference numbers	Consistency between protocol and coordinating center SOPs
10.4 Statistical Analysis, subsection Sensitivity Analysis	Added a sensitivity analysis to account for participants who qualified for the study pre-enrollment halt, but do not qualify for the study at baseline measures	New sensitivity analysis to allow already enrolled participants to stay in the study
11.1 subsection Data collection and Management Responsibilities	Clarified best practice for case report forms	Clarified procedure
Table 5	Updated to coincide with DCOC SOPs	Make protocol consistent with DCOC SOPs

Summary of Key Changes from Version 07 to Version 08

Affected Section(s)	Summary of Revisions Made (07 to 08)	Rationale
7.1 Consent, Screening, Baseline, and Post-Intervention, subsection *Screening*	Added the list of equipment participants will receive for remote height and weight measurements.<br><br>Added a statement that participants who qualify for the study will keep the remote height and weight equipment, and participants who do not qualify for the study will return the equipment in a study-team provided postage-paid box.	Addition requested by UAMS IRB
7.1 Consent, Screening, Baseline, and Post-Intervention, subsection *Baseline Measurements*	Added a phrase to specify when baseline measurements will end.	Clarify process

Summary of Key Changes from Version 08 to Version 09

Affected Section(s)	Summary of Revisions Made (08 to 09)	Rationale
1.3 Schedule of Activities, SOA 2	Updated: 1st column, 2nd row to allow list to contain patients seen in past 12-24 months<br><br>Added: Header row, Baseline column – added asterisks<br><br>Changed: Day 0 to Week 0<br><br>Added: footer – added explanation for asterisks	Allow more potential participants to be contacted<br><br>Allow for more time to screen participants<br><br>Allow more time for randomization and participant communication
Section 5.1 Inclusion Criteria	Last sentence, last paragraph<br>Deleted: 6 month	
5.5 Strategies for Recruitment and Retention, subsection Recruitment Option 1: Consecutive	Increased number of months patient visit data could include – from 12 to 24 months	Allow more potential participants to be contacted
5.5 Strategies for Recruitment and Retention, subsection Recruitment Goals and Monitoring	Deleted the text below from the 3rd paragraph, 2nd sentence<br>"and we will ask all clinics to recruit the exact number of missing participants using the recruitment option at which the clinic was most successful"	Clarify process
7.1 Consent, Screening, Baseline, and Post-intervention	Added: Screening and Baseline subsection. Explains that participants enrolled before the enrollment halt must have baseline measurements taken during the baseline period. Participants enrolled after the halt, should have screening done during the consent/screening period, but can conduct screening during baseline period, in conjunction with baseline measurements.	Clarification of process

Affected Section(s)	Summary of Revisions Made (08 to 09)	Rationale
7.1 Consent, Screening, Baseline, and Post-intervention, subsection Screening	2nd paragraph, 3rd sentence<br><br>Added: The ECHO ISPCTN site coordinator or staff member will mail or ship participants the scale and tape measure listed below or suitable replacements, approved by the iAmHealthy leadership team. Please see the iAmHealthy MOP for a complete list of equipment.<br><br>Deleted: The ECHO ISPCTN site coordinator or staff member will mail participants the equipment listed below or suitable replacements, approved by the iAmHealthy leadership team.<br><br>Bulleted list of equipment: deleted<br><br>Fiskars 01 005358 Wooden Ruler (model number 01 005358)<br><br>Scotch® Home and Office Masking Tape, 3/4 inch x 54.6 yards tan (3436)<br><br>No residue blue masking tape, 9 pack blue painter tape 2 inches x 15 yard/roll for decoration and painting, art and craft<br><br>Ticonderoga my first pencils, wood-cased #2 HB soft, pre-sharpened with eraser	Provide flexibility for study equipment
9.1 Efficacy Assessments, subsection Secondary Outcome Measures	#2, 2nd paragraph<br><br>Changed: six months to post-intervention	Clarify time point

Summary of Key Changes from Version 09 to Version 10

Affected Section(s)	Summary of Revisions Made (09 to 10)	Rationale
SOA 2	Expanded asterisk definition to include that height and weight measurements captured during the baseline period would be used for screening and baseline	Clarify definition

Affected Section(s)	Summary of Revisions Made (09 to 10)	Rationale
5.5, Subsection Stopping Rule	Clarified stopping rule to include the baseline period in the assessment and updated the number of participants reached to consented and screened-in participants	Per DSMB request
5.5, Subsection Additional Retention Plans	Removed the phrase “and before the intervention begins”	Protocol correction
5.5, Subsection Touch-point Cards	Added that cards could be sent via USPS or FedEx	Changed this to add flexibility
7.1, Subsection ActiGraph Activity Monitor and Diet Recall	Deleted that this has to occur after screening	Clarification of Process
7.1, Subsection Baseline Measurements	Added when feasible to the 4th sentence specifying that height and weight measurements have to be taken in triplicate	Clarify process
7.2 Randomization	Updated the measures that must be complete to randomize a participant	Per DSMB and clarification of randomization criteria
7.3, Subsection Administration of Newsletter Intervention	Add that newsletters could be sent via FedEx, or suitable replacement	Provide flexibility in process
9.2, Subsection Time Period and Frequency for Event Assessment	changed operational PI to ECHO ISPCTN site investigator in 3rd paragraph	Protocol correction

Summary of Key Changes from Version 10 to Version 11

Affected Section(s)	Summary of Revisions Made (10 to 11)	Rationale
4.4	Removed last sentence in this section	Remove lost to follow up process
5.5, Subsection Retention Plans	Removed specific weeks from retention tables. Added feedback phone call question to weeks 9-12	Provide more time to mail newsletter sent and include feedback call during weeks 9-12
8.2	Removed lost to follow up as a criterion for participant discontinuation/withdrawal and process for lost to follow up.<br><br>Removed lost to follow up process	Allow participants to stay in study longer

Affected Section(s)	Summary of Revisions Made (10 to 11)	Rationale
9.2, Subsection Time Period and Frequency for Event Assessment and Follow-up	Removed lost to follow up as a criterion for stop tracking severe AEs.<br><br>Changed unblinded iAmHealthy coordinator to unblinded ECHO ISPCTN coordinator	Removed lost to follow up from protocol and from severe AE process.<br><br>Protocol correction

Summary of Key Changes from Version 11 to Version 12

Affected Section(s)	Summary of Revisions Made (11 to 12)	Rationale
7.3, Subsection Group Sessions	Changed that dieticians will lead makeup sessions instead of psychologist/social worker.	Protocol correction
8.2 Participant Discontinuation/Withdrawal from the Study	Added that ECHO ISPCTN site coordinators, site investigators, or unblinded ECHO ISPCTN coordinators can ask child/caregiver pairs why they chose to withdraw from the study and that the pair can choose not to answer this question.	Process update

Summary of Key Changes from Version 12 to Version 13

Affected Section(s)	Summary of Revisions Made (12 to 13)	Rationale
1.3, Schedule of Activities (SOA) 2	Added 4 weeks to post column for activity monitor return and activity monitor data entry.<br><br>Added footnote that study leadership can grant an extension to complete post-intervention activities.	Extension added because shipping and postal delays can cause a 2-week delay in sites receiving activity monitors.<br><br>Allow sites extra time to complete post-intervention activities under extenuating circumstances.
5.5, Retention plans	Changed when the last card is sent to participants.	To allow more time to send cards

TABLE OF CONTENTS

STATEMENT OF COMPLIANCE 20
Glossary of Terms 20
Table of Abbreviations....21
1 PROTOCOL SUMMARY 23
1.1 SYNOPSIS 23
1.2 Schema 27
1.3 Schedule of Activities (SOA) 28
SOA 1 28
SOA 2 29
2 INTRODUCTION 31
2.1 Study Rationale 31
2.2 Background....31
Pediatric Obesity among Rural Populations 31
Rationale for Feasibility Study and Objectives 32
Treatment of Pediatric Obesity 34
Rationale for Newsletter Control Arm 34
Rationale for Individual-Level Assignment 35
Rationale for Clinic-level assignment 35
2.4 Risk/Benefit Assessment 35
Known Potential Risks 35
Known Potential Benefits 36
Assessment of Potential Risks and Benefits 36
3 OBJECTIVES AND ENDPOINTS....36
4 STUDY DESIGN 38
4.1 Overall Design 38
4.2 Scientific Rationale for Study Design....39
Primary Objective 39
Secondary Objectives 39
Tertiary Objectives 40
4.3 Justification for Intervention Model....40
Justification for Delivering the iAMHealthy Intervention via Mobile Technology 40
iAmHealthy Behavioral Intervention in Clinic Settings....41
4.4 End of Study Definition 42
5 STUDY POPULATION....42
5.1 Inclusion Criteria 42
5.2 Exclusion Criteria 43
5.3 Lifestyle Considerations 44
5.4 Screen Failures 44
5.5 Strategies for Recruitment and Retention 44
Enrollment Halt 44
Recruitment of Clinics 44
Randomization of Clinics 45
Recruitment of Participants 46
Retention Plans 49
Tracking Retention Plans....51
6 TRAINING....52
6.1 Height and Weight Inter-Rater Reliability 52
6.2 Clinical Helpline and Email....53
7 STUDY INTERVENTION....53
7.1 Consent, Screening, Baseline, and Post-Intervention 53

Consent/Assent and Other Informational Documents Provided to Participants 53
Screening and Baseline 55
Post-intervention Measurements 57
7.2 Randomization 57
7.3 Administration of Feasibility Study Intervention 57
Newsletter Intervention (Both Arms) 58
iAmHealthy Behavioral Intervention 58
Acquisition and Preparation of Tablets 61
Selection of Tablets and Internet Providers 61
Tablet Delivery and Storage 62
7.4 Measures to Minimize Bias: Randomization and Blinding 62
7.5 Study Intervention Compliance 62
7.6 Concomitant Therapy 63
8 STUDY INTERVENTION DISCONTINUATION AND PARTICIPANT DISCONTINUATION/WITHDRAWAL 63
8.1 Discontinuation of Study Intervention 63
8.2 Participant Discontinuation/Withdrawal from the Study 64
9 STUDY ASSESSMENTS AND PROCEDURES 64
9.1 Efficacy Assessments 64
Primary Outcome Measures 65
Secondary Outcome Measures 66
Tertiary Outcomes 67
Other Measures 67
9.2 Adverse Events/Serious Adverse Events 68
Definition of Adverse Events 68
Definition of Serious Adverse Events 68
Classification of an Adverse Event 69
Time Period and Frequency for Event Assessment and Follow-Up 70
Adverse Event Reporting 71
Serious Adverse Event Reporting 71
Reporting Events to Participants 72
Events of Special Interest 72
Reporting of Pregnancy 72
9.3 Unanticipated Problems 73
Definition of Unanticipated Problems 73
Unanticipated Problem Reporting 73
Reporting Unanticipated Problems to Participants 73
10 STATISTICAL CONSIDERATIONS 74
10.1 Statistical Hypotheses 74
Primary Objective 74
Secondary Objectives 74
10.2 Sample Size Determination 75
10.3 Populations for Analyses 76
10.4 Statistical Analyses 76
General Approach 76
Analysis of the Primary Endpoint(s) 76
Analysis of the Secondary Endpoint(s) 78
Analysis of Tertiary Endpoint(s) 78
Safety Analyses 79
Baseline Descriptive Statistics 79
Planned Interim Analyses 79
Sub-Group Analyses 79
Sensitivity Analysis 79
Tabulation of Individual Participant Data 79

11 SUPPORTING DOCUMENTATION AND OPERATIONAL CONSIDERATIONS 80
11.1 Regulatory, Ethical, and Study Oversight Considerations 80
Informed Consent Process 80
Study Discontinuation and Closure 80
Confidentiality and Privacy....80
Future Use of Stored Specimens and Data....81
Key Roles and Study Governance 82
Safety Oversight 83
Clinical Monitoring 83
Quality Assurance and Quality Control 84
Data Handling and Record Keeping....84
Study Deviations....85
Publication and Data Sharing Policy 85
Conflict of Interest Policy 86
Appendix A: SAMPLE NEWSLETTER 87
REFERENCES....89

STATEMENT OF COMPLIANCE

This trial will be carried out in accordance with International Conference for Harmonisation (ICH) Good Clinical Practice (GCP) and the United States (US) Code of Federal Regulations (CFR) applicable to clinical studies (45 CFR Part 46).

National Institutes of Health (NIH)-funded investigators and clinical trial staff who are responsible for the conduct, management, or oversight of NIH-funded clinical trials have completed Human Subjects Protection and ICH GCP Training.

The Data Coordinating and Operations Center (DCOC) will submit the protocol, informed consent form(s), recruitment materials, and all participant materials to the Central Institutional Review Board (cIRB) for review and approval. ECHO ISPCTN site awardees must use the cIRB-approved protocol and informed consents. The Central IRB must approve any amendment to the protocol before site awardees implement changes to the trial. In addition, all changes to the consent form must be cIRB approved; the DCOC will determine if site awardees need to obtain a new or revised consent form from participants who provided consent on a previously approved consent form.

GLOSSARY OF TERMS

We use the following terms throughout this protocol and fully explain them in the relevant sections of the protocol.

Backup Assessor: someone from the ECHO ISPCTN site who will take over blinded assessments in the event that the primary ECHO ISPCTN site coordinator is unblinded for any reason.

Blinded Assessor: The ECHO ISPCTN site coordinator and research-trained designated backup.

Body Mass Index (BMI): A person's weight in kilograms divided by his or her height in meters squared. NIH now defines normal weight, overweight, and obesity according to BMI, rather than the traditional height/weight charts.

Body Mass Index z-score (BMIz): A relative measure of child weight adjusted for age and sex. BMIz refers to the number of standard deviations away from the median BMI for a reference population of a specific age and sex.

Body Mass Index adjusted z-score (BMIaz): A relative measure of child weight calculated similarly to BMIz, but that adjusts for compression over 97th percentile. In this study, BMIaz is calculated according to the method of Freedman.[1]

Clinic: One of the four primary care clinics where the ECHO ISPCTN site awardee personnel will consent participants.

Clinic nurse: The nurse contact employed at the primary care clinic.

Clinic provider: The healthcare provider contact at each clinic.

Consecutive recruitment option (recruitment option 1): Clinic personnel will approach potential participants based on past (retrospective) or current (prospective) appointments.

Consented Participants: Participants who have completed the consent process.

DCOC: Data Coordinating and Operations Center for the IDeA States Pediatric Clinical Trials Network.

ECHO ISPCTN site awardee(s): The institution from an IDeA state that received a U24 grant to participate in the ECHO ISPCTN.

ECHO ISPCTN site coordinator: The research/nurse coordinator employed at the ECHO ISPCTN site awardee who will coordinate the iAmHealthy feasibility trial in the IDeA state in which the coordinator is employed.

ECHO ISPCTN site investigator: The site principal investigator for the iAmHealthy project.

EDC: electronic data capture

iAmHealthy behavioral intervention: In addition to receiving the American Academy of Pediatrics (AAP) newsletter, child/caregiver pairs participating in the iAmHealthy behavioral intervention arm will receive group sessions and individual sessions with the iAmHealthy behavioral intervention team via an electronic tablet provided by the sponsor.

iAmHealthy behavioral intervention arm: The intervention arm that will receive the iAmHealthy behavioral intervention and AAP newsletter for six months.

iAmHealthy behavioral intervention teams: Each team will be composed of a dietician, who will lead the individual session, and either a fully licensed psychologist (PhD or MA) or a licensed clinical social worker, who will lead the group sessions, with experience in weight management. These teams will be responsible for delivering the group sessions and the individual sessions to the iAmHealthy behavioral intervention arm.

Newsletter intervention: A behavioral intervention in the form of an AAP newsletter that all participating child/caregiver pairs, whether they are in the newsletter-only intervention arm or in the iAmHealthy behavioral intervention arm, will receive for each month of this six-month trial.

Newsletter-only intervention arm: The intervention arm that will receive only the AAP newsletter for six months.

Primary Caregiver: The adult/adults principally responsible for the participating child, typically a parent.

Randomized Participants: Participants who have completed the consent process as well as the screening and baseline measures and have been randomized to an intervention arm.

Screen Failure: Participants who were evaluated for participation in the study but did not meet the criteria for participation.

Traditional recruitment option (recruitment option 2): Methods focused on participant self-identification, such as hanging flyers in clinics, distributing flyers at check-in, and any other common contact methods used to reach their patients, such as newsletters, email or social media, or other means as they deem appropriate, etc., with IRB approval.

Unblinded ECHO ISPCTN coordinator: A research coordinator within the ECHO ISPCTN who will know randomized participant intervention arm assignment (unblinded)and will inform participants of their intervention arm, will make monthly contact participants to inquire about severe AEs and SAEs, and will record severe AEs and SAEs.

USPSTF: United States Preventive Services Task Force. An independent group of national experts in prevention and evidence-based medicine that works to improve the health of all Americans by making evidence-based recommendations about clinical preventive services such as screenings, counseling services, or preventive medications.

TABLE OF ABBREVIATIONS

AAP	American Academy of Pediatrics
AE	Adverse Event
ALCOA-C	Attributable, Legible, Contemporaneous, Original, Accurate and Complete (standard practice for completing source documents per ICH E6(R2))
BMI	Body Mass Index
BMIaz	Body Mass Index adjusted z-score
BMIz	Body Mass Index z-score

CAPA	Corrective and Preventative Action
CFR	Code of Federal Regulations
cIRB	Central Institutional Review Board
DCOC	Data Coordinating and Operations Center
DSMP	Data Safety Monitoring Plan
DSMB	Data Safety and Monitoring Board
ECHO	Environmental Influences on Child Health Outcomes
FDA	Food and Drug Administration
GCP	Good Clinical Practice
HIPAA	Health Insurance Portability and Accountability Act
ICC	intraclass correlation coefficient
ICH E6 (R2)	International Conference for Harmonisation Good Clinical Practice Integrated Addendum
IDeA	Institutional Development Award
ECHO ISPCTN	Environmental influences on Child Health Outcomes IDeA States Pediatric Clinical Trials Network
ITT	Intent-to-Treat
KUCTT	University of Kansas Center for Telemedicine and TeleHealth
MOP	Manual of Procedures
NCT	National Clinical Trial
NIH	National Institutes of Health
OHRP	Office for Human Research Protections
PHI	Protected Health Information
PI	Principal Investigator
PP	Per-Protocol Population
QA	Quality Assurance
QC	Quality Control
RCT	Randomized Controlled Trial
RUCA	Rural-Urban Commuting Area Codes
SAE	Serious Adverse Event
SAP	Statistical Analysis Plan
SMART IRB	Streamlined, Multisite, Accelerated Resources for Trials IRB Reliance platform
SOA	Schedule of Activities
SOP	Standard Operating Procedure
TAU	Treatment As Usual
TBD	To Be Determined
UAMS	University of Arkansas for Medical Sciences
UP	Unanticipated Problem
UPIRTSO	Unanticipated Problems Involving Risks To Subjects or Others
US	United States
USDA	United States Department of Agriculture
USPSTF	United States Preventive Services Task Force

1 PROTOCOL SUMMARY

1.1 SYNOPSIS

Title: Feasibility Trial of the iAmHealthy Intervention for Healthy Weight in Rural Children Recruited from Primary Care Clinics

Study Description: Conduct a multisite feasibility trial for the iAmHealthy intervention with two randomized controlled recruitment options (consecutive recruitment and traditional recruitment). We will examine retention, dose, and blinding that will inform the development of a larger, treatment-outcome fully powered randomized controlled trial (RCT) of the iAmHealthy behavioral intervention in the ECHO ISPCTN.

Objectives: **Primary Objectives.** Conduct a rigorous feasibility trial for a future RCT (iAmHealthy versus newsletter) in four clinics affiliated with ECHO ISPCTN site awardees to assess key variables: participant recruitment, participant retention, intervention dose, and blinding.

Objective 1. Participant Recruitment Rate

A) Evaluate the two proposed recruitment options to determine which are feasible for recruitment of rural participants. Hypothesis: The percent of randomized participants, among those contacted with each recruitment option, will be greater than 20%.

B) Compare the two proposed recruitment options. Hypothesis 1: The consecutive recruitment option (option 1) will yield a higher percent of participants randomized than those contacted through the traditional recruitment option (option 2). Hypothesis 2: Option 1 will require less "time to full recruitment" than option 2.

Objective 2. Participant Retention - Evaluate the percentage of randomized participants that remain in the study through the final measurement time point (sixth month) in both arms (i.e., iAmHealthy behavioral intervention and newsletter only). Hypothesis: The study will retain greater than 75% of the randomized participants through the final measurement (sixth month) in each arm.

Objective 3. Dose - Evaluate the percentage of participants in the iAmHealthy behavioral intervention arm and retained through the final measurement who receive a sufficient dose (i.e., 80% [20.8 hours] of the 26 planned contact hours) of the intervention. Hypothesis: 80% of the participants randomized to the iAmHealthy behavioral intervention arm who are retained through the final measurement will receive a sufficient dose (i.e., 80% [20.8 hours] of the 26 planned contact hours) of the intervention.

Objective 4. Staff Blinding - Evaluate the agreement between the blinded assessor's estimation of participant arm assignment and actual participant arm assignment. Hypothesis: The study will achieve blinding in both arms, as determined by calculating the New Blinding Index score with a 95% confidence interval that includes 0.[2,3]

Secondary Objectives

1. Obtain a preliminary estimate of effectiveness of the iAmHealthy behavioral intervention on change in child BMIaz, compared to the newsletter-only intervention, from the baseline measurement to the final measurement time point (sixth month).
2. Obtain a preliminary estimate of the effectiveness of the iAmHealthy behavioral intervention on change in child BMI, compared to the newsletter-only intervention, from the baseline measurement to the final measurement time point (sixth month).
3. Obtain a preliminary estimate of the effectiveness of the iAmHealthy behavioral intervention on change in primary caregiver BMI, compared to the newsletter-only intervention, from the baseline measurement to the final measurement time point (sixth month).
4. Obtain a preliminary estimate of the effectiveness of the iAmHealthy behavioral intervention on child nutrition (i.e., fruits, vegetables, etc.; see Section 7.2, subsection *Individual Sessions*), compared to the newsletter-only intervention, from the baseline measurement to the final measurement time point (sixth month).
5. Obtain a preliminary estimate of the effectiveness of the iAmHealthy behavioral intervention on moderate to vigorous physical activity (child), compared to the newsletter-only intervention, from the baseline measurement to the final measurement time point (sixth month).

Tertiary Objectives

1. Assess the reasons that parents decline to participate in the iAmHealthy study and whether those reasons differ by recruitment option or clinic.
2. Assess recruitment options by determining the total number of participants randomized via each recruitment option at each clinic and assess whether each clinic met its goal of at least eight randomized participants via each recruitment option.

Endpoints:

Primary Endpoints

1. Whether or not each participant contacted was randomized
2. Whether or not each randomized participant attended the final (sixth month) measurement
3. The number of contact hours received by participants randomized to the iAmHealthy intervention and whether or not these participants attended the final (sixth month) measurement
4. Each blinded staff member will indicate to which intervention arm each participant was assigned

Secondary Endpoints

1. Measurement of change in BMIaz (child) from baseline to final measurement (sixth month)
2. Measurement of change in BMI (child) from baseline to six-month measurement (end of intervention)
3. Measurement of change in primary caregiver BMI from baseline to final measurement (sixth month)
4. Measurement of change in the number of servings of sugar-sweetened beverages, servings of “red foods” (i.e., foods with ≥7 grams of fat and/or ≥12 grams of sugar per serving), and servings of fruits and vegetables, as measured by 24-hour food recall (child), at baseline and final measurement (sixth month)
5. Measurement of change in number of minutes of moderate to vigorous physical activity, as measured by physical activity monitoring (child), from baseline to final measurement (sixth month)

Tertiary Endpoints

1. For each child whose parent(s) decline participation, reasons for nonparticipation will be collected from the parent(s)
2. For each clinic, the number of randomized participants for each recruitment option will be determined

Study Population: We estimate that we will approach up to 560, with the goal of randomizing 112 participants (Per clinic [4 clinics]: 28 participants, 14 participants per treatment arm); Children ages 6 to 11 years of age with BMI ≥85th percentile.

Phase: N/A

Description of Sites/Facilities Consenting Participants: The study team will perform the iAmHealthy trial in four ECHO ISPCTN site states. Specifically, the study team will perform the iAmHealthy trial in one clinic per state.

Recruitment Options: Randomization of recruitment options occurs at the clinic level. Each clinic, in a randomly ordered sequence, will implement two methods of recruitment. See Section 5.5, subsection *Recruitment of Participants*, for more detail on the methods of the two recruitment options.

**Description of Study Intervention:**	Randomization of participants into intervention arms will occur at the individual level.<br><br>**Newsletter-only Intervention Arm**<br>Child/caregiver pairs in the newsletter-only intervention arm will receive:<br>• usual care at their clinic for all issues, including overweight and obesity;<br>• a monthly newsletter that focuses on general child health. The child/caregiver pairs will receive six newsletters (one per month) during the six-month intervention period.<br><br>**iAmHealthy Behavioral Intervention Arm**<br>Child/caregiver pairs in the iAmHealthy behavioral intervention arm will receive:<br>• usual care at their clinic for all issues, including overweight and obesity;<br>• a monthly newsletter that focuses on general child health. The child/caregiver pairs will receive six newsletters (one per month) during the six-month intervention period (same newsletter provided to the newsletter-only intervention participants);<br>• weekly group sessions for the first three months, followed by monthly group sessions during the next three months, for a total of 15 group sessions. The trial's iAmHealthy behavioral intervention psychologist or social worker (one team per clinic) will deliver these group sessions via an electronic tablet that the trial sponsor will supply;<br>• 11 hours of individual "homework" sessions with the iAmHealthy behavioral intervention dietician.<br><br>Note: The iAmHealthy behavioral intervention psychologists/social worker will individualize group sessions and the iAmHealthy behavioral intervention dietician will adapt individual sessions by focusing on local cultural, religious, and ethnic factors relevant to the recommended changes in eating and activity habits.<br><br>Per the 2017 USPSTF guidelines, the iAmHealthy behavioral intervention includes 26 contact hours (15 hours of group sessions and 11 hours of individual sessions).
**Participant Duration:**	From randomization to sixth months

1.2 SCHEMA

Figure 1 shows the planned schema of activities during this feasibility trial. Section 5.5, subsection *Recruitment of Participants*, details the participant recruitment options and implementation.

Clinic Recruitment	Participating ECHO ISPCTN-site awardees recruit clinics in their state that meet the specified clinic-eligibility criteria. ↓	
4-week Run-up Period	The four participating clinics will provide patient-visit data (i.e. billing data and electronic medical record) to ECHO ISPCTN site staff who will organize this list from more recent clinic visit dates to older visit dates. ECHO ISPCTN site staff will use an honest broker, when required. Then, the list will be unduplicated by patient (potential participant list) and an abbreviated list returned to clinic staff to identify BMI percentiles (see Section 5.5, subsections *Recruitment Option 1 and Recruitment Option 2*) ↓	
Recruitment, Consent, & Screening (Weeks -10 through -5)	Four clinics will implement recruitment options 1 and 2 in random order, as assigned by the DCOC. ECHO ISPCTN site staff with the assistance of clinic staff will identify and contact potential participants (see Section 5.5, subsections *Recruitment Option 1 and Recruitment Option 2*). The ECHO ISPCTN site coordinator will screen and consent participants. (See Figures 3 and 4 for details about recruitment options and Table 2 for recruitment options randomization sequence.) By the time participant randomization occurs, participants will complete the 24-hour food recall and physical activity monitoring. ↓	
Baseline Measures (Weeks -4 through -1)	The ECHO ISPCTN site coordinator will obtain height and weight measurements of child participant and primary caregiver. ↓	
Randomization (Day 0)	Participant randomization (Both groups will receive usual care throughout the trial.) ↓	↓
	iAmHealthy behavioral intervention ↓	Newsletter-only intervention ↓
Months 1-3 (Weeks 1-12)	• Weekly sessions<br>• Monthly newsletter<br>• Individual sessions (homework) ↓	• Monthly newsletter ↓
Months 4-6 (Weeks 13-24)	• Monthly sessions<br>• Monthly newsletter<br>• Individual sessions (homework) ↓	• Monthly newsletter ↓
Post-Intervention Assessment (Weeks 25-28)	• 24-hour food recall and physical activity monitoring<br>• Height and weight of child and primary caregiver	• 24-hr food recall and physical activity monitoring<br>• Height and weight of child and primary caregiver

Figure 1. Schema of the iAmHealthy Feasibility Trial

1.3 SCHEDULE OF ACTIVITIES (SOA)

SOA 1

The SOA 1, shown below, is the pre-coronavirus enrollment pause SOA. Please see SOA 2 for the post-enrollment pause schedule of activities.

		Screening/ Baseline Measures		Intervention							Post
**Trial Period**	**4-week run-up**	**Consent/Screening Weeks -15 to -3**	**Baseline* Weeks -3 to -1**	**Day 0**	**Week 1-4**	**Week 5-8**	**Weeks 9-12**	**Weeks 13-16**	**Weeks 17-20**	**Weeks 21-24**	**Weeks 25-28**
Clinic provide patient-visit data	X										
Develop list of consecutively seen patients in past year and who meet eligibility criteria	X										
Inclusion/Exclusion Criteria		X									
Informed Consent/Assent		X									
Randomization				X							
Height[a,b*]			BA								BA
Weight[a,b*]			BA								BA
24-hour food recall[c,d,e]		X									X
Physical activity monitoring[c,f]		BA									BA
Newsletter[a]					X	X	X	X	X	X	
Weekly session (for iAmHealthy behavioral intervention arm)[a,d,g]					X	X	X				
Monthly session (for iAmHealthy behavioral intervention arm)[a,d,h]								X	X	X	
Individual sessions (iAmHealthy behavioral intervention arm)[a,d]					11 hours of homework help via remote technology						
Severe AE, SAE, and UP review					X	X	X	X	X	X	X
Calculation of participant recruitment rate, retention, dose, & New Blinding Index											X
Demographics[i]		X									
Technology feasibility[i]											X
Post-trial questionnaire[i]											X
Study Personnel Time Assessment		X	X								
Study Personnel Satisfaction Survey				X							X

Abbreviations: AE=adverse event; SAE=serious adverse event; UP=unanticipated problem; BA=blinded assessor
[a] child and caregiver; [b] at clinic; [c] child only; [d] remotely; [e] Participants will complete one three-day 24-hour food recall at baseline and one three-day 24-hour food recall (three days is two week days [Mon. – Fri.] and one weekend day [Sat. or Sun.] at post-intervention. [f] Collected via ActiGraph monitor and data transferred to USB and downloaded by site coordinator. [g] There must be at least five days, but not more than nine days, between weekly sessions. [h] There must be at least three weeks, but not more than five weeks, between monthly sessions. [i] Caregiver will complete.
* Consent and baseline activities can occur during the same visit.

SOA 2

The table below shows the schedule of activities once enrollment resumes.

Trial Period		Screening/Baseline Measures		Intervention							Post	
	4-week run-up	Consent/Screening Weeks -10 - -5	Baseline* Weeks -4 - -1	Week 0	Weeks 1-4	Weeks 5-8	Weeks 9-12	Weeks 13-16	Weeks 17-20	Weeks 21-24	Weeks 25-28**	Weeks 29-33***
Clinic provide patient-visit data	X											
Develop list of consecutively seen patients in past 12-24 months and who meet eligibility criteria	X											
Inclusion/Exclusion Criteria		X										
Informed Consent/Assent		X										
Randomization				X								
Height[a*]			BA								BA	
Weight[a*]			BA								BA	
24-hour food recall[c,d,e]		X									X	
Physical activity monitoring[c,f]		BA									BA	BA
Newsletter[a]					X	X	X	X	X	X		
Weekly session (for iAmHealthy behavioral intervention arm)[a,d,g]					X	X	X					
Monthly session (for iAmHealthy behavioral intervention arm)[a,d,h]								X	X	X		
Individual sessions (for iAmHealthy behavioral intervention arm)[a,d]					11 hours of homework help via remote technology							
Severe AE, SAE, and UP review					X	X	X	X	X	X	X	
Calculation of participant recruitment rate											X	
Calculation of participant retention											X	
Calculation of dose											X	
Calculation of New Blinding Index											X	
Demographics[i]		X										
Technology feasibility[i]											X	
Post-trial questionnaire[i]											X	
Study Personnel Time Assessment		X	X									
Study Personnel Satisfaction Survey				X							X	

Abbreviations: AE=adverse event; SAE=serious adverse event; UP=unanticipated problem; BA=blinded assessor

[a] child and caregiver; [c] child only; [d] remotely; [e] Participants will complete one three-day 24-hour food recall at baseline and one three-day 24-hour food recall (three days is two week days [Mon. – Fri.] and one weekend day [Sat. or Sun.] at post-intervention. [f] Collected via ActiGraph monitor and data transferred to USB and downloaded by site coordinator. [g] There must be at least five days, but not more than nine days, between weekly sessions. [h] There must be at least three weeks, but not more than five weeks, between

* Consent/screening and baseline activities can occur during the same visit. If this occurs, height and weight measurements will be used for screening & baseline

**Sites may collect activity monitors, score activity, and record activity monitor data in source document and EDC. Sites may not send activity monitors to participants during weeks 29-33.

***Under extenuation circumstances, the study-team leadership may grant a site an extension for post-intervention activity completion.

Table 1. Data and Collector

Data	Source	Collector	Blinded/Unblinded
Eligibility: Patient-visit data	Medical records and/or billing data	Clinic staff and ECHO ISPCTN site research team	Unblinded/NA collected prior to randomization
List of consecutively seen patients, potential participants, for potential consent/assent	Medical records and/or billing data, if needed supplemented with chart review	Clinic staff and ECHO ISPCTN site staff	Prior to randomization
Parental reasons for declining to participate	Primary caregiver	ECHO ISPCTN site coordinator	Blinded/NA collected prior to randomization
Height/Weight	Participants	ECHO ISPCTN site coordinator	Blinded
24-hour food recall	Participants	Dietetics & nutrition team member	Blinded
Physical activity monitoring	ActiGraph data	ECHO ISPCTN site coordinator	Blinded
Attendance at weekly and individual sessions	Participants who attended each session	iAmHealthy behavioral intervention team	Unblinded
Demographic information	Demographic questionnaire	Primary caregiver provides via link	Unblinded
Contamination effects and newsletter use	Post-trial questionnaire	Primary caregiver provides via link	Unblinded
Technology problems	Technology feasibility (as part of group and individual session attendance forms and post-trial parent questionnaire)	iAmHealthy behavioral intervention team<br>Primary caregiver	Unblinded
ECHO ISPCTN site and clinic staff satisfaction with recruitment options, intervention arms, and trial	Study Personnel Satisfaction Survey	DCOC	Unblinded
BMI/BMIaz	BMI and BMIaz: Standard formulas/equations	ECHO ISPCTN site coordinator	Blinded
Potential participants	Recruitment option 1: patients seen in the last year or scheduled patients who meet inclusion criteria<br><br>Recruitment option 2: not defined	ECHO ISPCTN site coordinator	Blinded/NA collected prior to randomization
Potential participants approached	Recruitment option 1: # of participants approached by ECHO ISPCTN site coordinator<br><br>Recruitment option 2: # of self-referred potential participants	ECHO ISPCTN Site awardee coordinator	Blinded/NA collected prior to randomization
Participants randomized	# of participants randomized from each site-associated clinic	Unblinded ECHO ISPCTN coordinator	Unblinded
Estimation of treatment assignment	ECHO ISPCTN site coordinator and backup	N/A: ECHO ISPCTN site coordinator and backup will enter their answers into EDC	Blinded
AEs/SAEs	Participant, primary caregiver, ECHO ISPCTN site study personnel, or behavioral intervention team members	Unblinded ECHO ISPCTN coordinator	Unblinded

2 INTRODUCTION

2.1 STUDY RATIONALE

Feasibility Trial. A goal of the ECHO ISPCTN is to involve rural children in state-of-the-art clinical trials.[4,5] The ECHO ISPCTN explores and develops its ability to reach consent, and randomize rural children and families in these trials, and as such, the proposed feasibility trial will serve as a model for reaching rural children through their primary care clinic. Rural children are difficult to reach for clinical trials. Utilizing primary care clinics takes advantage of clinics' access to well and sick rural children as well as their influence on needed care.[6,7] The proposed feasibility trial will explore different options for identifying and consenting rural children in clinical trials through primary care clinics (i.e. participant recruitment rate), while also measuring retention, dose, and blinding. The long-term goal of the feasibility trial in four clinics in the ECHO ISPCTN states is to assess the feasibility of conducting a large, fully powered treatment-outcome trial of iAmHealthy in all 17 ECHO ISPCTN states. There is an iAmHealthy trial in process in rural schools (R01 NR016255), but it has several limitations that we could address by a trial in primary care clinics. Namely, the school-based trial struggled to reach school-level recruitment goals and hence school-based interventions of this nature may be limited in their ability to reach all segments of the target population. Further, the limitation of school-based recruitment may result in a study population that yield different study outcomes from those observed in a clinic-based sample. A large clinic-based trial may help reach segments of the population that the school-based study team could not reach, and a large clinic-based trial could help determine if study outcomes might vary across the two populations. However, before we can conduct a fully powered trial, we must gather important information regarding participant recruitment, participant retention, intervention dose, and blinding in clinics serving rural children. Thus, these are the foci of the current feasibility study.

Obesity. It is also important to note that delivering obesity treatment would address a pressing issue in the health of rural children and their families. The prevalence of pediatric obesity is high among all children and disproportionately affects children from rural and/or medically underserved areas.[8,9] There are few interventions specifically targeting pediatric obesity treatment among rural and medically underserved children, a factor that contributes to health disparities. Previous research indicates that mobile health (mHealth), the use of mobile technology for the purposes of medical care, is feasible and acceptable for the delivery of pediatric obesity interventions to rural/medically underserved children.[10-12] This type of intervention lowers children's body mass index z-score (BMIz) and helps children and families improve their health behaviors. However, the trial detailed herein extends this research in a new and innovative direction—it moves the iAmHealthy mobile technology intervention into family homes, simultaneously increasing convenience and dose (i.e., contact hours). As described in more detail elsewhere (see Section 7.2, subsection *Description of the iAmHealthy Behavioral Intervention*), the iAmHealthy behavioral intervention is composed of group and individual/family-level components, based on new research that indicates this combination is highly effective. [13]

2.2 BACKGROUND

Pediatric Obesity among Rural Populations

The iAmHealthy behavioral intervention, for which this study acts as a feasibility pilot, addresses pediatric obesity. The prevalence and incidence of pediatric obesity in the US remains a critical public health concern. Data from the Centers for Disease Control and Prevention (2015-2016) indicate that 18.5% of US children and adolescents (ages 2 to 19 years) are classified as obese (BMI >95th percentile) and 35.1% are either overweight or obese (BMI ≥85th percentile).[14] Childhood obesity continues to be

associated with short-term health consequences and long-term risk for obesity and other health problems.[15]

Pediatric obesity disproportionately affects children in rural areas.[16] According to the 2015 National Survey of Children's Health, children living in rural areas are more likely to be overweight and obese when compared to urban children (38% rural versus 30% urban children).[17] Rural children experience higher rates of overweight and obesity than their urban counterparts, and rural children have a 50% higher probability of being obese than urban children.[18] Similarly, research completed in Kansas concurred that there was a higher prevalence of pediatric obesity in rural areas than in urban areas. In Kansas, 43% of rural children were either overweight or obese.[19]

Although prevention efforts can be helpful, experts agree that treatment programs are necessary to combat the pediatric obesity epidemic.[20,21] Few treatments target children and families from rural areas or those who live in medically underserved areas. These individuals face unique challenges, such as having to travel longer distances to receive intensive obesity care, lack of nutrition education, poor access to healthcare providers, lower socioeconomic status, and fewer opportunities for physical exercise.[16] Research indicates that there are also differences in the health behaviors of rural and urban children (e.g., rural children are more likely to eat junk food, skip breakfast, engage in lower levels of moderate to vigorous physical activity, and engage in higher rates of sedentary activity than their urban counterparts).[19]

Rationale for Feasibility Study and Objectives

There are many challenges to conducting clinical trials. Medically underserved populations, including rural families, have low participation in clinical trials.[22] Also, traditional methods of recruitment in clinical trials may result in samples that are not representative of the relevant population.[23] Among both urban and rural patients, common patient-reported barriers to participation in clinical trials include fear of clinical trials, concerns about side effects, limited understanding of clinical research, limited incentives for participation, and mistrust of healthcare system. Rural participants emphasized the time commitment for participation.[24] Interestingly, rural participants were more motivated to enroll by benefit to their family while urban patients mentioned individual benefit while both groups agreed that financial incentives would encourage participation.[24] The most common investigator-reported barriers include communication about and awareness of clinical trials by participants.[22] Experts agree, "building clinical trials around patients in their homes and community through remote visits and monitoring could enhance recruitment and increase convenience for participants." Recruitment of clinics that care for rural patients and the recruitment of rural patients themselves into research can be challenging, which may be why rural participants are under-represented in clinical trials.[25] However, some research indicates that engagement of local resources and providers as research sites can increase participation, [16] and that rural clinics may be more likely to engage in network-type research. Regarding participant recruitment specifically into behavioral obesity trials, common recruitment barriers include family and study logistics, family economics, lack of provider interest, invasive protocols, stigma, clinician time restraints, lack of patient interest, and lack of acceptance of weight status.[26]

Bjorn et al examined clinical trials that used different methods of recruitment.[23] These researchers compared clinical trials involving adult cardiac participants that research staff consecutively screened and enrolled to similar trials that used traditional recruitment methods. Bjorn et al found that consecutive enrollment resulted in better enrollment (17 to 26% versus 4 to 7% with traditional recruitment), in a trial population that was systematically different, and in better outcomes than the traditionally enrolled trials.[23] Our recruitment option 1 (Figure 2) involves consecutive screening and

consenting, while option 2 is a more traditional screening and consenting. This will allow us some insight into how different recruitment options affect the populations consented and their outcomes.

Researchers have proposed facilitators to enhance recruitment into clinical trials, including accommodating visits outside of regular clinic hours, incentivizing participants, cultivating relationships with clinics in advance of recruitment, emphasizing potential benefits to participants, and, for obesity trials, focusing on health, rather than obesity.[26] Other research has indicated that the main reasons individuals enroll in clinical trials are physician recommendation to participate in a clinical trial, expectation of personal benefit, and altruism.[27] In terms of the most frequently used recruitment options, the USPSTF systematic review indicates that 26% of studies used specific screening methods, 21% relied only on physician referral, and most studies used multiple methods of recruitment within each clinic.[28] This is important as other research indicates that relying on multiple methods of recruitment can result in a more representative sample.[29]

A trial that reported on the recruitment options that we are testing, the "Obesity Prevention Tailored for Health (OPT)" study[30], a project of Kaiser-Permanente of Southern California, enrolled families with a healthy 11 to 12 year-old child, irrespective of overweight or obesity. Periodically, researchers randomly selected potential participants from patient lists. Study personnel mailed parents of these children introductory letters and then contacted them; the patient's physician knew about the study and reviewed the recruitment list but did not participate in recruitment. Overall, study personnel sent 4,730 letters. These letters resulted in 222 (4.7%) of the potential participants being ineligible, 1,360 (28.8%) declined, 2,385 (50.4%) were uncontactable, and 763 (16.1%) accepted. Of the 763 who accepted, study personnel scheduled 66.8% of the potential participants for a baseline appointment. Note that of the 763 potential participants who accepted, study personnel consented and randomized 361 (47.3%), which was 7.6% of the potential participants initially contacted.[30] The most frequent reason for not participating in the study was lack of interest from the parent or child (49.6%), with 23.1% citing lack of time to participate. Results indicate that factors associated with trial participation include children being overweight or obese, the child or parent having had more outpatient visits, and the child or parent having received influenza vaccine.[30]

Another trial, Hamilton-Shield et al, reported on the recruitment of obese children, 5 to 11 years of age, into the "Community Mandolean Randomised Controlled Trial (ComMando)" in the United Kingdom.[31] One of the specific aims of this pilot project was to assess recruitment strategies. The recruitment strategies assessed were general practitioner-initiated recruitment, healthcare professional-initiated recruitment, recruitment through advertisement and record screening, and recruitment through school. Except for recruitment through advertisement, all of these recruitment strategies relied on on-site staff to identify, screen, and refer potentially eligible participants. The intervention in this trial was intensive. It involved participants putting food on a plate connected to a computer, and researchers measuring the change in meal weight over time. Using general-practitioner mailouts, study personnel sent 10,077 mailouts and 116 responded (1.2%). Of the 116 respondents, 37 (31.9%) were randomized. General practitioner and healthcare professional referral recruitment methods resulted in 35 respondents with 12 (34.3%) randomized. For all other recruitment strategies, there were 31 responses and 12 (38.7%) randomized.

Both Hamilton-Shield and Ghai have shown that there are substantial challenges to recruitment. We are likely to encounter these same challenges, along with the challenges associated with recruiting participants who live in rural areas. However, unlike Hamilton-Sheild, [31] we have enhanced our likelihood of success by relying on ECHO ISPCTN awardee-sites for recruitment, rather than solely relying on clinic personnel for participant identification and recruitment. We will compare two recruitment strategies previously used in clinical trials. Recruitment option 1 will be a consecutive recruitment

strategy where clinic personnel will approach potential participants based on past (retrospective) or current (prospective) appointments. Recruitment option 2 will be a traditional recruitment strategy. Using both strategies will provide key information on the relative effectiveness of each strategy, something rarely studied in clinical trials research. To optimize success, we will approach patients who recently visited the participating clinic (option 1), which should enhance our ability to contact them, avoiding the contact issues Ghai encountered by using random selection from patient lists.[30] Through these methods, our trial will answer the question: What is the efficacy of each recruitment strategy in primary care clinics for rural participants?

Beyond recruitment, additional challenges include concerns regarding retention of patients to study completion, retention of patients in the treatment group to receive the proper “dose”, and staff blinding in small rural clinics. Regarding retention, data from the USPSTF systematic review indicate that retention rates across studies range from 63.4% to 100%.[28] Another published literature review indicates that average retention rates in pediatric obesity trials are 86%,[32] and data from a recent three-arm trial, with a similar intervention dose to our proposed trial, show retention rates of 92%, 97%, and 89% for each of the three arms.[33] However, we know little about retention of rural parents and children in a behavioral obesity intervention trial, especially when researchers recruit these individuals from their primary care clinics. Because ECHO ISPCTN researchers understand that rural patients and families face greater barriers to participation, it stands to reason that these rates may be lower than those found with non-rural participants. Regarding dose, it stands to reason that if rural families encounter more barriers to participation in research, then they may also have difficulty achieving a sufficient dose of the proposed behavioral obesity intervention. Previous research indicates that several factors may influence retention in obesity trials, including the amount of child body mass change, child starting body mass, child quality of life, and parent educational level.[34] However, these factors may not have been studied in rural populations. Another concern is maintaining proper staff “blind” in small rural clinics, whereby the staff recording the height and weight measurements remain blinded to patient/family arm assignment, although there is little published research regarding this concern.[14] In this trial, we will add to the literature in this area and increase our likelihood of maintaining staff blind by blinding the ECHO ISPCTN site coordinator (and ECHO ISPCTN site backup assessor), not the clinic staff.

Treatment of Pediatric Obesity

The most common treatment for pediatric obesity is family-based behavioral groups. Research consistently demonstrates that these treatments are among the most effective and scalable treatments for pediatric obesity. [18,35,36] The recent USPSTF review indicates that these types of treatments are more effective if parents participate in treatment, include at least 26 hours of intervention contact, focus on healthy nutrition and physical activity, and include the use of behavioral and cognitive-behavioral techniques, such as goal setting, self-monitoring, positive reinforcement, and stimulus control.[28] It is also important to note that group treatment is very practical, allowing a single healthcare provider to treat 8 to 10 families in a single one-hour session. Recent research indicates that including individual coaching sessions with families to help them overcome family-level factors can improve treatment success of these behavioral groups.[18,37] Our study will build on this research by incorporating child/caregiver group and individual sessions delivered via video conference (similar to Skype) on tablets that will focus on healthy behaviors.

Rationale for Newsletter Control Arm

Experts agree that the most appropriate control arm for effectiveness trials is treatment as usual (TAU).[38] However, TAU is not appropriate for many ECHO ISPCTN site awardees that indicate they must

offer some sort of treatment to all participants. A recent review shows that a common alternative to TAU is enhanced "standard care," which is used in approximately 44% of obesity treatment trials,[39] including those published by experts[39-42] and those in NIH-funded projects (e.g., HD073237 Bentley; DK084331 Elder; MD007634 Farrell). This type of control arm "most often involved the receipt of standard off-the-shelf print material addressing health behaviours."[39] Data indicate that these types of control arms cause negligible effects on weight (median = +0.1 kg; IQR = −0.85 kg to +0.8 kg), and discussions with key stakeholders indicate that this type of intervention will increase palatability to participants and providers across the ECHO ISPCTN. We will us an enhanced standard-of-care control that will include a newsletter intervention and will also serve as a retention device (see Section 5.5), since research indicates that repeated contact is key to retention in longitudinal clinical studies.[40]

Rationale for Individual-Level Assignment

This study will randomize participants at the individual level, rather than the clinic level. In individual level randomization, it is important to guard against contamination of treatment arms, as this is a serious threat to validity.[43] Contamination occurs when individuals do not receive their assigned intervention, and in this case, when there is a potential for the newsletter-only arm to receive a behavioral intervention. In school-based treatment and prevention studies focused on pediatric obesity, there is some evidence of contamination across the assigned arms.[44] However, in clinic-based studies, such as those in the 2017 USPSTF literature review, the risk of contamination was extremely low. The current study further decreases this risk since all treatment takes place via a tablet in participant homes, and individuals in the newsletter-only arm cannot access these tablet-delivered sessions.

Rationale for Clinic-level assignment

We will randomly implement two recruitment options in each clinic so that, in each recruitment month, two clinics will implement each recruitment option (Table 2). This will allow us to compare recruitment options within and between clinics. The purpose of studying two recruitment options is not to compare one to another. Rather, it is to ensure that both recruitment options result in sufficient number of participants and should be included in the larger trial. This is especially useful since clinics differ in many aspects. In an examination of 61 clinics in 8 practice-based research networks, substantial differences were shown between practices by within practice clustering of factors of interest, including demographics, age (intraclass correlation coefficient [ICC]=.151), race (ICC=.265), behaviors, and unhealthy diet (ICC=.206).[45] There is also substantial variation between practices in the delivery of medical treatments, such as immunizations. When examining 15 clinics from 11 states in a national practice-based research network, researchers found that immunization rates varied by 40 to 50 percentage points, depending on the measure used.[46] The proposed random order of implementation within each clinic will allow us to compare the recruitment options within and between clinics, balancing the clinic-level differences.

2.4 RISK/BENEFIT ASSESSMENT

Known Potential Risks

A potential identified risk for this trial is loss of confidentiality. There is also the possibility that participants could engage in unhealthy weight loss habits, feel burdened by participation, or concerned that participating may negatively affect family interactions. There is also a possibility that participants could experience minor sprains or strains because of physical activity recommendations; however, the

risk of sprains or strains because of physical activity is relatively small compared to the health risks faced by participants who engage in no physical activity at all.

These concerns are not supported as a potential risk by the 2005 summary of the evidence from the USPSTF.[47] The 2010 USPSTF systematic review also identified no reported harms from obesity management behavioral interventions.[48]

Known Potential Benefits

The primary potential benefits of trial participation include health education and obesity treatment, which may lead to improved health habits and, later, to improved health. There could also be improvement in caregiver and child self-efficacy and social cohesion of families participating in the group intervention, as well as a lessening of family conflict about health behaviors. However, research has not documented these additional benefits.

Assessment of Potential Risks and Benefits

The trial has minimal possible risks, and the potential benefits of health education and obesity treatment outweigh the potential risks.

3 OBJECTIVES AND ENDPOINTS

OBJECTIVES	ENDPOINTS	JUSTIFICATION FOR ENDPOINTS
**Primary**		
Objective 1. Participant Recruitment Rate – Hypothesis: A) The percent of randomized participants, among those contacted with each recruitment option, will be greater than 20%.<br>Hypotheses: B) 1. The consecutive recruitment option (option 1) will yield a higher percent of participants randomized than those contacted through the traditional recruitment option (option 2). Hypothesis 2. Option 1 will require less "time to full recruitment" than option 2.	Whether or not each participant contacted was randomized	This percentage is a measure of the feasibility of each of the recruitment options
Objective 2. Participant Retention – Hypothesis: The study will retain greater than 75% of the randomized participants through the final measurement (sixth months) in each arm.	Whether or not the each randomized participant attended the final (sixth month) measurement	This percentage is to determine if participants consented through one recruitment option are more likely to be retained, as compared to participants consented through the other recruitment option
Objective 3. Dose – Hypothesis: 80% of the participants randomized to the iAmHealthy behavioral intervention arm who are retained through the final	The number of contact hours received by participants randomized to the iAmHealthy intervention and whether or	This percentage is a measure of the feasibility of the proposed

OBJECTIVES	ENDPOINTS	JUSTIFICATION FOR ENDPOINTS
measurement will receive a sufficient dose (i.e., 80% [20.8 hours] of the 26-planned contact hours) of the intervention.	not these participants attended the final (sixth month) measurement	iAmHealthy behavioral intervention
Objective 4. Staff Blinding - Hypothesis: The study will achieve blinding in both arms, as determined by calculating the New Blinding Index 95% confidence interval that includes 0.[2,3]	Each blinded staff member will indicate to which intervention arm each participant was assigned	The Index is a measure of the feasibility of the proposed blinding measures
**Secondary**		
1. Obtain a preliminary estimate of effectiveness of the iAmHealthy behavioral intervention on change in child BMIaz, compared to the newsletter-only intervention, at the final measurement time point (sixth month) and the baseline measurement.	Measurement of change in BMIaz (child) from baseline to final measurement (sixth month)	Change in BMIaz at sixth months is a measure of intervention effectiveness
2. Obtain a preliminary estimate of the effectiveness of the iAmHealthy behavioral intervention on change in child BMI, compared to the newsletter-only intervention, from the baseline measurement to the final measurement time point (sixth month).	Measurement of change in BMI (child) from baseline to six-month measurement (end of intervention)	Change in BMI at sixth months is a measure of intervention effectiveness
3. Obtain a preliminary estimate of the effectiveness of the iAmHealthy behavioral intervention on change in primary caregiver BMI, compared to the newsletter-only intervention, from the baseline measurement to the final measurement time point (sixth month).	Measurement of change in primary caregiver BMI from baseline to final measurement (sixth month)	Change in primary caregiver BMI is a measure of intervention effectiveness
4. Obtain a preliminary estimate of the effectiveness of the iAmHealthy behavioral intervention on child nutrition (i.e., fruits, vegetables, etc.; see Section 7.2, subsection *Individual Sessions*), compared to the newsletter-only intervention, from the baseline measurement to the final measurement time point (sixth month).	Measurement of change in the number of servings of sugar-sweetened beverages, servings of “red” foods (i.e., foods with ≥7 grams of fat and/or ≥12 grams of sugar per serving), and servings of fruits and vegetables, as measured by 24-hour food recall (child), at baseline and final measurement (sixth month)	Food recall for 24-hour periods (taken during three days) may explain changes in BMI
5. Obtain a preliminary estimate of the effectiveness of the iAmHealthy behavioral	Measurement of change in number of minutes of moderate to vigorous physical activity, as measured by physical activity	Physical activity monitoring may explain changes in BMI

OBJECTIVES	ENDPOINTS	JUSTIFICATION FOR ENDPOINTS
intervention on moderate to vigorous physical activity (child), compared to the newsletter-only intervention, from the baseline measurement to the final measurement time point (sixth month).	monitoring (child), from baseline to final measurement (sixth month)	
**Tertiary**		
1. Assess the reasons that parents decline to participate in the iAmHealthy study and whether those reasons differ by recruitment option or clinic.	For each child whose parent(s) decline participation, reasons for nonparticipation will be collected from the parent(s)	This is a measure of the feasibility of each of the recruitment options
2. Assess participant randomization by determining the total number of participants randomized via each recruitment option at each clinic and assess whether each clinic met its randomization goal of at least eight randomized participants via each recruitment option.	For each clinic, the number of randomized participants for each recruitment option will be determined	This is a measure of the feasibility of each of the recruitment options
**Abbreviations:** BMI=body mass index; BMIaz=body mass index (adjusted z-score)		

4 STUDY DESIGN

4.1 OVERALL DESIGN

This feasibility trial is a multisite, RCT of two different methods of identifying and recruiting participants into the iAmHealthy behavioral intervention. Children and caregivers in both arms will receive the usual care for all existing medical conditions throughout the trial period. They will also receive a monthly newsletter for six months (see Section 7.2; subsection *Newsletter Intervention*). In addition to the newsletter, child/primary caregiver pairs in the iAmHealthy behavioral intervention arm will receive both group and individual sessions (see Section 7.2, subsection *iAmHealthy Behavioral Intervention*). The trial intervention period will last for six months, and it will consist of 15 hours of group sessions and 11 hours of individual sessions (see Section 7.2, subsection *Description of iAmHealthy Behavioral Intervention*) for 26 contact hours. This falls within the 26- to 51-contact hour category, which is significantly more effective than the 6- to 25-contact hour category, per the recent USPSTF guidelines.[49] This feasibility trial will provide information toward the implementation of a fully powered, multisite, randomized behavior intervention trial that will compare the effectiveness of the iAmHealthy behavioral intervention to a newsletter-only intervention for the treatment of obesity among rural and underserved children and their primary caregivers.

See the Participant Schedule of Activities in Section 1.3 for a more detailed overview of time points for the activities noted above.

4.2 SCIENTIFIC RATIONALE FOR STUDY DESIGN

The objectives and hypotheses for this trial are as follows.

Primary Objective

Primary care practices often differ dramatically in their patient demographics, patient behaviors, [45] and delivery of needed services.[46,50] Because of these differences between the patients and capabilities of practices, we will test recruitment options both within and between clinics. This will be a feasibility trial for the iAmHealthy intervention with randomized controlled recruitment options. We will examine retention, dose, and blinding that will inform the development of a larger, fully powered RCT of the iAmHealthy behavioral intervention in the ECHO ISPCTN. The objectives and hypotheses for this trial are as follows.

Primary Objective: Conduct a rigorous feasibility trial of a future RCT (iAmHealthy versus newsletter) in four clinics affiliated with ECHO ISPCTN site awardees to assess key variables (participant recruitment rate, participant retention, intervention dose, blinding)

Objective 1. Participant Recruitment Rate.

A) Evaluate the two proposed recruitment options to determine which are feasible for recruitment of rural participants. Hypothesis: The percent of randomized participants, among those contacted with each recruitment option, will be greater than 20%.

B) Compare the two proposed recruitment options. Hypothesis: The consecutive recruitment option (option 1) will yield a higher percent of participants randomized among those contacted than the traditional recruitment option (option 2).

Objective 2. Participant Retention. Evaluate the percentage of randomized participants that remain in the study through the final measurement time point (sixth month) in both arms (i.e., iAmHealthy behavioral intervention and newsletter only). Hypothesis: The study will retain greater than 75% of the randomized participants through the final measurement (sixth month) in each arm.

Objective 3. Dose. Evaluate the percentage of participants in the iAmHealthy behavioral intervention arm and retained through the final measurement who receive a sufficient dose (i.e., 80% [20.8 hours] of the 26 planned contact hours) of the intervention. Hypothesis: 80% of the participants randomized to the iAmHealthy behavioral intervention arm who are retained through the final measurement will receive a sufficient dose (i.e., 80% [20.8 hours] of the 26 planned contact hours) of the intervention.

Objective 4. Staff Blinding. Evaluate the agreement between the blinded assessor's estimation of participant arm assignment and actual participant arm assignment. Hypothesis: The study will achieve blinding in both arms, as determined by calculating the New Blinding Index score with a 95% confidence interval that includes 0.[2,3]

Secondary Objectives

1. Obtain a preliminary estimate of the effectiveness of the iAmHealthy behavioral intervention on change in child BMIaz, compared to the newsletter-only intervention, from the baseline measurement to the final measurement time point (sixth month).
2. Obtain a preliminary estimate of the effectiveness of the iAmHealthy behavioral intervention on change in child BMI, compared to the newsletter-only intervention, from the baseline measurement to the final measurement time point (sixth month).

3. Obtain a preliminary estimate of the effectiveness of the iAmHealthy behavioral intervention on change in primary caregiver BMI, compared to the newsletter-only intervention, from the baseline measurement to the final measurement time point (sixth month).
4. Obtain a preliminary estimate of the effectiveness of the iAmHealthy behavioral intervention on child nutrition (i.e., fruits, vegetables, etc.; see Section 7.2, subsection *Individual Sessions*), compared to the newsletter-only intervention, from the baseline measurement to the final measurement time point (sixth month).
5. Obtain a preliminary estimate of the effectiveness of the iAmHealthy behavioral intervention on moderate to vigorous physical activity (child), compared to the newsletter-only intervention, from the baseline measurement to the final measurement time point (sixth month).

Tertiary Objectives

1. Assess the reasons that parents decline to participate in the iAmHealthy study and whether those reasons differ by recruitment option or by clinic.
2. Assess recruitment options by determining the total number of patients randomized via each recruitment option at each clinic and assess whether each clinic met its goal of at least eight randomized participants via each recruitment option.

4.3 JUSTIFICATION FOR INTERVENTION MODEL

For the feasibility trial, we will evaluate two proposed recruitment options (see Section 5.5, subsection *Recruitment of Participants*, for a description of recruitment options). Based on published literature, as well as own clinical trials work and our conversations with ECHO ISPCTN site awardees, the two proposed recruitment options are the ones most used in clinics recruiting for clinical trials.

The NIH ECHO ISPCTN is ideally suited to study participant recruitment, participant retention, intervention dose, and staff blinding, as well as for the delivery of an empirically supported pediatric obesity treatment program to rural families through primary care clinics. Previous research indicates that primary care clinics are the most trusted informational source for parents regarding their child's health and medical needs, and as such, it is the ideal venue for recruitment into pediatric obesity treatment services. Because the goal of the ECHO ISPCTN is to involve rural children in state-of-the-art clinical trials,[4] the current proposal will serve as a feasibility trial not only of iAmHealthy, but more broadly of future clinical trials recruiting rural children from primary care clinics.

Justification for Delivering the iAMHealthy Intervention via Mobile Technology

Although the use of mobile health technology in intervention research is innovative in and of itself, research indicates that many clinics and centers are now building these types of infrastructures to reach their patients for clinical purposes, making the technology widely available for other uses. Specific to obesity treatment, experts agree that face-to-face or in-person obesity treatment can be expensive[47] and even impractical for most rural and medically underserved families, making health technology interventions especially appealing. Outside of the medical system, adults increasingly use mobile technologies across varying geographies and socioeconomic statuses, with two-thirds of Americans having smartphone access and 42% of adults owning tablets.[51] Data also indicate that 74.4% of all families in the US have Internet in their homes, and 83.8% have a computer.[52] Finally, data also indicate that 80% of all Internet users have searched for a health-related topic online,[53] indicating that many consumers are already using technology to look for answers regarding health issues.

Delivery of health services to pediatric patient homes with mobile health technology has been widely recommended across various disease states.[54,55] A recent Strategic Policy Agenda by the American Heart Association listed delivery of treatment research to rural populations using telehealth and mobile health technology as a major strategic priority for 2017 to 2020.[56] However, no studies exist regarding the direct delivery of pediatric obesity treatment to family homes with mobile health technology, making this area of study extremely innovative. It is surprising that no studies exist in this area given that experts uniformly agree that technology-based approaches, such as that proposed in the iAmHealthy trial, are both cost effective and highly scalable. Previous research suggests that home-based technology approaches not only save significant costs for patients and families but also save costs for providers and healthcare systems. In a recent review of the literature,[57] the authors concluded that the limited evidence suggests mobile health technology is a "promising approach to pediatric weight management, particularly for rural families with limited access to treatments."[57] The iAmHealthy trial will allow the ECHO ISPCTN to capitalize on the growing use of technology-based approaches by conducting the first study to determine the effectiveness of an empirically supported intervention for pediatric obesity that will be delivered directly into rural family homes.

The iAmHealthy trial will be a multicomponent behavioral intervention primarily composed of group and individual sessions for caregivers and children. These sessions will focus on nutrition, physical activity, and behavior change. The iAmHealthy behavioral intervention teams (one per clinic) will deliver these sessions via video conference to participants who will remotely attend through study-provided tablets. Current 2017 data indicate that among American adults 88% use the Internet and more than 75% have broadband access at home, and these proportions are even higher (>95% for Internet use) in the likely age range of parents who will participate in this trial.[58] If the outcome of this trial is positive, we will be able to disseminate iAmHealthy to future children and families via their own devices and home broadband access. We will assess participating caregivers' Internet availability and usage to facilitate future dissemination in the unique communities served in this trial.

Note: Data suggest that Internet services are widely available; however, there is a possibility that these data are not representative of Internet use/availability among the unique populations available in the ECHO ISPCTN. Therefore, we will provide tablets equipped with cellular data service to child/caregiver pairs to equalize performance of the video conference software that we will use to deliver the group and individual sessions. For more information, see Section 7.2; subsection *Acquisition and Preparation of Tablets*.

iAmHealthy Behavioral Intervention in Clinic Settings

Adequately addressing overweight and obesity in children requires investigation in many settings, including those in which children typically access medical care. In this vein, we will test the iAmHealthy behavioral intervention in primary care clinics to determine if we can overcome the barriers encountered when delivering iAmHealthy in the currently funded iAmHealthy Schools R01 (NR016255), which targets rural elementary school children and their families in Kansas. Of note, primary care clinics have a fundamental interest in preventing, identifying and treating obesity. In the iAmHealthy Schools trial, our team developed a research program using interactive video conference (similar to Skype) to treat pediatric obesity among rural children and families. These studies led to the development of the iAmHealthy behavioral intervention. Data[59,60] for previous participants in rural schools indicate that the mean child BMIz score decreased from 1.88 (SD = 0.52) to 1.76 (SD = 0.52) in the treatment arm ($t = 3.02$, $P = .007$).

The iAmHealthy Schools trial has experienced strong participant recruitment and retention, with 30% to 50% of eligible children/caregivers consented and enrolled, and 97.2% (n = 36) of treatment participants

and 100% of control arm participants (n = 38) are still active. (Active for the treatment arm equals attending monthly weigh-ins and more than 80% of intervention sessions; active for the control arm equals attending monthly weigh-ins.) Because the study is in the initial phases, outcome data are unavailable. Feasibility data, however, indicate that delivering this type of intervention via technology to rural families is highly feasible, and participants spontaneously report high satisfaction with the intervention and the mobile technology.

However, the iAmHealthy Schools trial has also highlighted the many difficulties in delivering this type of treatment program through schools. While approximately 85% of US children attend public schools, schools must be active participants in the project to reach these children. This has proven challenging because state funding of kindergarten through 12th-grade schools has sharply declined since the 2008 recession, there is a significant increase in students with a simultaneous decrease in teachers, and the No Child Left Behind law, passed in 2002, increased testing and achievement standards. These stressors have heightened schools laser focus on educational outcomes and achievement, making schools challenging partners in health promotion programs, like iAmHealthy. In the current iAmHealthy Schools trial, we have completed two annual school recruitment cycles, and thus far, both years have fallen short of our school recruitment goals (which are separate from our participant recruitment goals). The major barrier reported by schools, who expressed interest but subsequently did not participate, was lack of time.

In the proposed iAmHealthy study, we will attempt to determine if targeting primary care clinics and consenting their patients will show a difference in the population reached and intervention effect, as compared to the results in iAmHealthy Schools. Data indicate that children and families are more likely to engage in health-related activities recommended by their medical provider, and that health promotion programs conducted through schools and those conducted through medical clinics may reach somewhat different population segments. In addition, because pediatric obesity is so prevalent, many have argued that offering (and therefore, testing) health promotion programs across many sectors, including both schools and medical clinics, is necessary.

4.4 END OF STUDY DEFINITION

The trial will end at the time point when all child/caregiver pairs complete the six-months of intervention (as shown in the Schedule of Activities in Section 1.3), technology feasibility survey, and post-trial questionnaire.

5 STUDY POPULATION

5.1 INCLUSION CRITERIA

To be eligible to participate in the proposed trial, a child/caregiver pair must attend a qualifying primary care clinic and meet all of the following inclusion criteria.

1. **Child is rural.** The child lives in a rural area, as defined by the United States Department of Agriculture (USDA) Rural-Urban Commuting Area (RUCA) codes (greater than or equal to 4). We will calculate this using the 2010 Zip Code, RUCA Code crosswalk.

2. **Child is ages 6-11 years at the time of consent.** At the time of consent, a child must be 6 to 11 years of age. A narrow age range is necessary to decrease developmental variability. ECHO ISPCTN site coordinators may consent a child no earlier than her or his sixth birthday (6 years, 0 months, 0 days), and may consent a child up to her or his 12th birthday (11 years, 11 months, 30 days).

3. **Child BMI percentile is ≥85th.** We will use the 85th percentile as a minimal cutoff for participation, as this is the minimal criteria for the definition of overweight. There is no upper limit on BMI percentile for inclusion.

4. **Child and primary caregiver speak English.** For the initial feasibility pilot, we will ask that both the child and primary caregiver speak English. For the larger trial that will follow, we will accommodate Spanish-speaking participants.

5. **For the iAmHealthy behavioral intervention arm, the child and primary caregiver are available when the behavioral intervention team offers the intervention for the participating clinic.** The child and primary caregiver must be available for most of the iAmHealthy behavioral intervention sessions when the behavioral intervention psychologist/social worker offers the group sessions at the participants' clinic. The behavioral team will conduct these sessions via video conference and families will attend via tablets.

Adult participants must be primary caregivers of child participants (biological or adoptive parent or legal guardian), and adult participants have no inclusion/exclusion criteria other than those stated in Sections 5.1 and 5.2. Only one adult identified as the primary caregiver will be the official measured participant, although any other adults in the household may also attend the sessions.

Note: There are no inclusion criteria regarding home technology; we will provide all required technology devices (including the tablet). The research team will supply participants with tablets equipped with cellular data services that can access the Internet even if the household does not have Internet service. We provide further information about selecting Internet providers to ensure connectivity in rural areas in Section 7.2, subsection *Selection of Tablets and Internet Providers*. Caregivers will complete the technology feasibility questionnaire (see Section 9.1, subsection *Other Measures*) at consent/screening/baseline (as part of the demographics questionnaire, *iAmHealthy Demographics Form*) and at the end of the intervention will assess technology available in homes for this feasibility trial and for future dissemination efforts.

5.2 EXCLUSION CRITERIA

We will exclude from the trial an individual who meets any of the following criteria.

1. **Child has a physical limitation or injury that substantially limits physical mobility or has a planned medical treatment during the course of the trial that will substantially limit physical mobility.** Because this trial recommends physical activity, we will exclude children who cannot comply with this health behavior.

2. **Child has a known medical issue that could affect protocol compliance (e.g., cancer).** If a child has a significant medical issue known to the clinic that could affect protocol compliance, we will exclude this child, as the protocol involves an intense intervention commitment.

3. **Child and/or primary caregiver has a developmental delay or cognitive impairment that could affect protocol compliance.** We will exclude primary caregivers and/or children with a known developmental delay, as this could negatively affect participation and measurement completion.

4. **Child is enrolled in a weight-loss trial.** If a child is enrolled in a weight-loss trial, we will exclude the child to avoid cross-pollination of trial interventions.

5. **Child has a sibling who has already consented in the trial.** If multiple children from the same family attempt to enroll, the statistical team will randomly select one child for consent. We will always allow siblings to attend the intervention, but we will not officially consent them.

5.3 LIFESTYLE CONSIDERATIONS

The iAmHealthy behavioral intervention arm will make a number of health recommendations to child/caregiver pairs that include improving healthy eating and diet choices, increasing physical activity, decreasing sedentary activity, and improving caregivers' behavior-management skills. This trial will also encourage primary caregivers to spend time reading the AAP newsletter on general child health.

In the iAmHealthy behavioral intervention arm, primary caregivers will need to participate in the group and individual sessions with their child. A culturally competent registered dietician will provide individual health coaching sessions to assist child/caregiver pairs with tailoring the general recommendations to meet the pairs' specific religious, ethnic, and/or cultural beliefs and customs. The training of the iAmHealthy behavioral intervention teams will ensure a standardized approach (see Section 7.4), and the constituents of the groups will influence the discussions, providing content that is specific to religious, ethnic, and/or cultural beliefs and customs (see Section 7.2 subsection *Individual Sessions*).

5.4 SCREEN FAILURES

We define screen failures as child/caregiver pairs who consent to screening for the trial but who do not meet all of the criteria for participation.

The information that ECHO ISPCTN site coordinators must record and retain for screen failures includes, but is not limited to, demography, screen failure details, and eligibility criteria.

ECHO ISPCTN site coordinators may rescreen individuals who do not meet the criteria for participation in this trial (i.e., screen failure), up to two times, until the end of the recruitment period. If rescreened child/caregiver pairs subsequently qualify for the trial, clinic sites should assign them the same participant number as at the initial screening. Once the intervention period begins, we will no longer allow individuals to rescreen. For the assessment of recruitment options, the individual will be assigned to the recruitment option under which the successful consent occurred.

5.5 STRATEGIES FOR RECRUITMENT AND RETENTION

Enrollment Halt

The iAmHealthy study team halted enrollment on March 13, 2020, due to the coronavirus pandemic, in an effort to protect the safety of study participants and personnel. Enrollment will resume when (1) the DSMB, NIH, and UAMS IRB approve the proposed changes for remote consent and baseline measures and extension of enrollment and baseline periods; and (2) all four sites can participate in remote research activities.

See sections 7.1, subsection *Participant Consent/Screening and Baseline Visits* for remote consenting and baseline measures.

Recruitment of Clinics

The ECHO ISPCTN site awardee will identify the primary care clinics selected for participation based on previous work and must meet these criteria:

- The clinic must have previously collaborated with an ECHO ISPCTN site awardee in research or quality improvement projects.
- The clinic must have at least 40% of its pediatric patient visits covered by Medicaid.[61]
- The clinic will qualify if they have seen a minimum of 100 potential participants (rural, 6 to 11 years of age) who are overweight or obese during the last year: We will recruit four clinics from among the participating ECHO ISPCTN site awardees, and we will individually enroll each clinic. For clinic qualification, we will assume that 33% of potential participants are overweight or obese (see Section 2.2, subsection *Pediatric Obesity among Rural Populations*).[17,19] This means that clinics will qualify if they have seen a minimum of 300 children who are rural and 6 to 11 years of age. We will provide instructions for clinic personnel to assess qualification criteria based on one year of patient-visit data. This data will include visit date, birthdate, and zip code. We will use zip codes to define rurality by using the zip code RUCA code crosswalk. Our instructions will include sending a file containing only zip codes to the research team for classification into rural versus not rural based on zip code RUCA code crosswalk (rural is RUCA ≥ 4, See 5.2 Inclusion Criteria). Clinic personnel perform qualification and research personnel do not view protected health information (PHI).
- The clinic must have an electronic medical records system.
- The clinic must have appropriate resources, including private space to consent participants, to hold height and weight measurement equipment, and to securely store, in a locked cabinet, tablets and activity monitors. The clinic must also have staff to serve as the nurse contact and backup person (approximately 10% time). The clinic nurse will not conduct study procedures such as data collection. The clinic nurse will only complete the following tasks: (1) provide contact information (of potential participants) to the ECHO ISPCTN site personnel, (2) provide IRB-approved recruitment materials to potential participants, and (3) collect names of potential participants who opt-out of the study.

We provide additional details on recruiting clinics in the MOP.

Randomization of Clinics

The DCOC will sequentially and randomly randomize clinics to the two participant recruitment options (see Table 2). The total recruitment period is two months (one month for each recruitment option).

Table 2. Clinic Randomization to Recruitment Options

**Clinic**	**Month**	
	**A**	**B**
**A**	1	2
**B**	2	1
**C**	1	2
**D**	2	1

Note: Two clinics will implement each recruitment option during the recruitment months.

Recruitment of Participants

For each clinic, the randomized participant goal will be a total of 28. The minimum is 16 participants, and the maximum is 32. We determined the upper and lower recruitment limits by the feasibility of leading groups via electronic tablet, and our experience suggests that the ideal group size is 8 to 16 participants. Individual randomization will occur within each clinic, and the DCOC will randomize child/caregiver pairs to the iAmHealthy behavioral intervention arm or to the newsletter-only intervention arm (see Section 7.3 for more information on randomization).

We describe a framework for evaluating consent in Primary Outcome Measures (Section 9.1). Figure 2 (below) provides the potential participant flow diagram for the different recruitment options in each clinic.

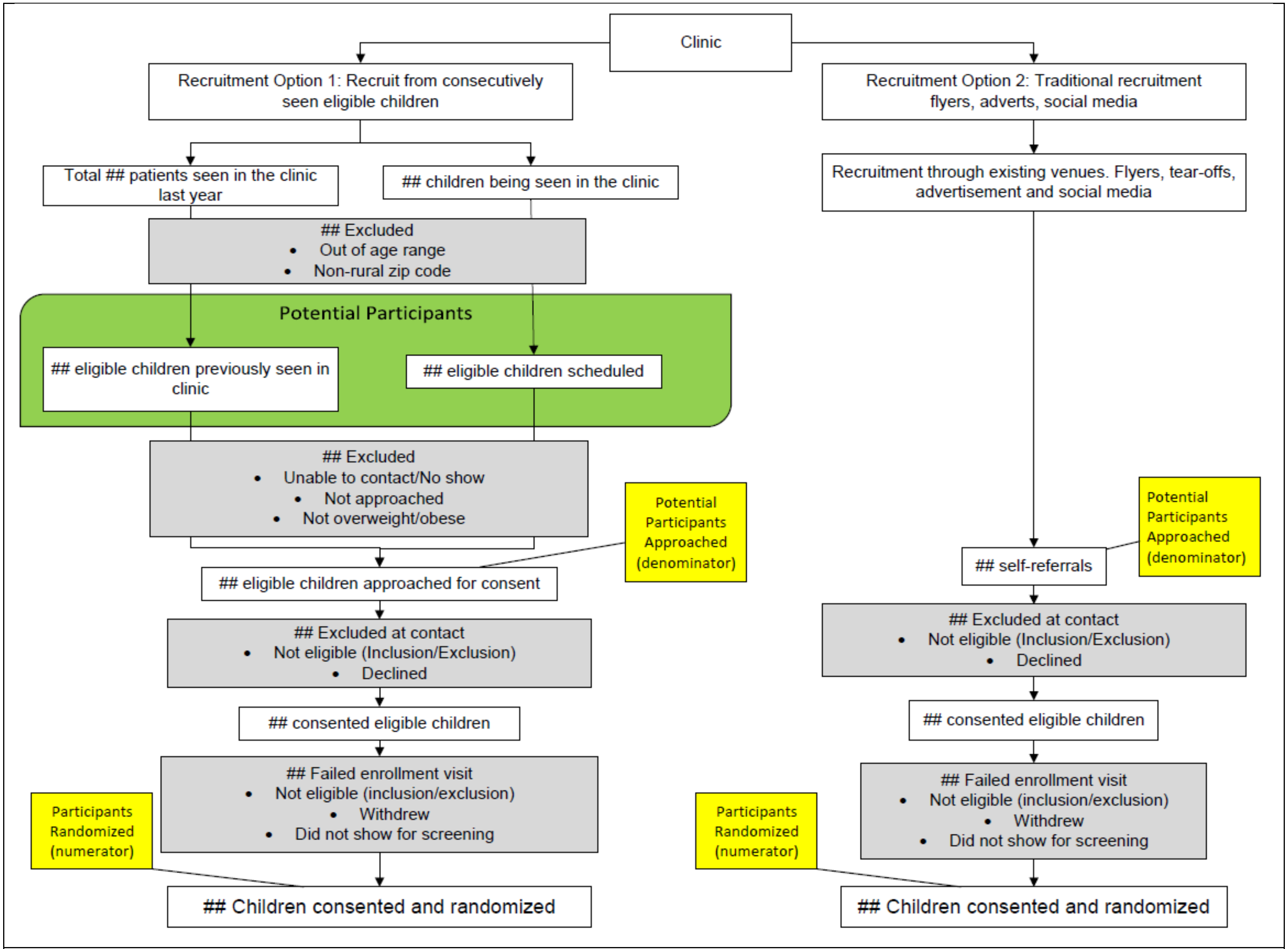

Figure 2. Potential Participant Flow Diagram

RECRUITMENT OPTION 1: CONSECUTIVE

Retrospective. There will be a four-week run-up period where clinical staff with the assistance of ECHO ISPCTN site research staff will search their electronic medical records and/or billing software to develop a list of patients seen consecutively by clinicians in the last year and who meet the inclusion criteria (see Section 5.1). Specifically, clinic staff will provide ECHO ISPCTN site staff with 12 to 24 months of patient-visit data (e.g., billing records). The site staff will organize this list from more recent clinic visit dates to older visit dates. Then, the list will be unduplicated by patient (potential participant list). Because we expect that clinics may not be able to easily classify patients as overweight or obese from billing data, the ECHO ISPCTN site research staff will group the list into 60 potential participant segments. Then, the ECHO ISPCTN site research staff will give the abbreviated list to the clinic staff and ask them to provide BMI percentiles within the last year for the potential participants. Once ECHO ISPCTN site research staff receive the BMI percentiles, they will begin approaching potential participants, and the clinic staff will begin providing BMI percentile for the next 60 most recently seen participants. ECHO ISPCTN site research staff will continue to provide abbreviated potential participant lists until enrollment has been satisfied. If the clinic staff can produce a list from electronic medical records that shows the BMI percentages of potential participants, this back and forth procedure is not required.

Prospective. From appointment data, each week, clinic staff will send ECHO ISPCTN site research staff a list that includes age and zip code of patients who medical personnel will see the coming week. ECHO ISPCTN site research staff will revise this list according to rurality and age to identify patients meeting inclusion criteria. Site research staff will then return the list to the clinic and ask clinic staff to approach patients who meet the inclusion criteria, including BMI percentile, during the patient's visit.

Potential participants may contact the ECHO ISPCTN site research staff or the clinic to inquire about the iAmHealthy trial while a clinic is implementing recruitment option 1. ECHO ISPCTN site research staff will develop a log to capture the self-referrals who inquire about the trial, and they will evaluate these potential participants for consent. The log will include how the potential participant learned about the study, the contact outcome (consented or not), and reason for not consenting, if applicable (e.g., out of age range, non-rural zip code, not overweight or obese, or declined).

RECRUITMENT OPTION 2: TRADITIONAL

Clinics will engage in traditional clinical trial recruitment methods focused on participant self-identification. The DCOC will provide a menu of options from which clinics can choose, such as hanging flyers in clinics, distributing flyers at check-in, newsletters, email, or social media, or other means in consultation with the DCOC communication team. The DCOC will obtain IRB approval for all recruitment materials. These recruitment materials will ask interested parties to contact ECHO ISPCTN site staff. For those potential participants who contact the clinic for the information, clinic personnel will provide information about the study (see next paragraph). As in recruitment option 1, ECHO ISPCTN site staff will develop a log to capture the self-referrals who inquire about the trial. The log will include how the potential participant learned about the study, the contact outcome (consent or not), and reason for not consenting, if applicable (out of age range, non-rural zip code, not overweight or obese, or declined).

POTENTIAL PARTICIPANTS WHO CONTACT THE CLINIC

For both recruitment options, if the caregiver of a potential participant contacts the clinic nurse or provider, he or she will provide basic information about the research trial to the caregiver/child pair. The clinic nurse or provider may send basic information by mail or email, or the nurse or provider may

present the information to the caregiver/child pair when they come to the clinic. The informational material will include contact information for the ECHO ISPCTN site staff.

RECRUITMENT GOALS AND MONITORING

There will be goals for each of the recruitment options. For each clinic, the recruitment goal for both options will be 28 participants total or 14 participants per treatment arm. The minimum recruitment for each recruitment option is eight participants (16 total), and the maximum for each recruitment option is 16 participants (32 total).

During recruitment, the DCOC and trial PIs will hold weekly calls between the ECHO ISPCTN site investigator, research staff, and clinic staff. The ECHO ISPCTN research staff affiliated with each clinic will present their progress on recruitment and receive supervision on tracking potential participants and potential participants approached, and participants consented. During these calls, if one of the clinics in a specific recruitment option has not met the minimum target (eight participants) and the other clinic in that recruitment option has reached the goal (14 participants), then we will ask the clinic meeting its goal to continue its current recruitment option up to a maximum of 16 participants.

At the end of recruitment month two, if sites have not met the randomized target (112 participants), we will notify all participating ECHO ISPCTN sites during the weekly call. We will extend the recruitment window for up to four weeks. If sites successfully recruits 16 participants in recruitment month two, sites can continue recruiting in the same recruitment option up to 32 participants without waiting for the four-week recruitment extension period. If sites are not successful in recruiting 16 participants, sites must continue recruiting in the recruitment option until 16 participants are recruited or the recruitment option ends. During the four-week recruitment extension, all sites can recruit participants using the recruitment option in which the site was most successful, up to the cap of 32 participants/site.

Stopping Rule. Our target is 112 randomized participants. At the end of 12 weeks of implementing the recruitment options and the baseline period, we will assess the overall recruitment. If we have not reached 64 consented and screened-in participants (minimum of eight per each intervention group per clinic, and four clinics), then study PIs will recommend stopping the iAmHealthy feasibility trial to the DSMB.

Retention Plans

Compensation. Participant families will receive compensation for their time. Sites will prorate this compensation based on the months of participation and will dispense compensation during the study. Each participant family will receive $20.00 when the site receives the signed informed consent and/or assent, if applicable. Participant families will also receive $20.00 for each month they remain in the study, during the six-month intervention period. When a participant family completes the post-intervention measurements, diet recalls, activity monitoring, and returns all study equipment (e.g., ActiGraph, tablet computer), the participant family will receive $100. The total possible compensation is $240 ($20 [consent] + $120 [$20 each month of intervention period] + $100 [post-intervention activities completed and equipment returned]).

Additional retention plans. After a participant gives consent, the unblinded coordinators will make retention calls approximately monthly to participants using IRB approved scripts. Additional processes to aid in retention are described in the following tables.

**Study Timeline**	
**Consent/Screening, Baseline, Day 0**	**Retention Contact**
After consent/screening	Thank-you card sent after screening
	Touch-point card sent with a message thanking the participant for her or his patience during the screening process. The message could be similar to the text below.<br><br>"Thank you for joining the iAmHealthy study! We appreciate your patience while we recruit other participants. Soon, you will receive information from our team about setting a time when you can come back to the clinic to measure your weight and height."
Randomization	Touch-point card sent with message similar to text below.<br><br>Congratulations! You have been placed in the XX (newsletter or electronic tablet) group.<br><br>Text for newsletter group: In about 4 weeks, you will receive in the mail a HealthyChildren.org newsletter. Please read this newsletter or have someone read it to you. You should receive a HealthyChildren.org newsletter every month for the next 6 months. You will receive a phone call from a member of our team who will ask about the newsletter, how you are doing in the study and if you have experienced any problems while participating in the study.<br><br>Text for electronic tablet group: In the next couple of weeks, you will receive an electronic tablet and instructions for getting the tablet ready for use. Please follow these instructions and contact the name and number on the instruction sheet if you have problems setting up the tablet. We will also contact you to set up the group sessions in which both you and your child will participate, and after that, to set up your individualized family health coaching sessions.<br><br>You will also receive a phone call from a team member monthly who will ask how you are doing in the study and if you have experienced any problems while participating in the study.

**Study Timeline**	
**Study Activated Weeks 1-12**	**Retention Contact**
After 1st newsletter sent	Follow-up card with message similar to text below<br><br>Thank you for continuing to participate in the iAmHealthy! We're glad to have you in our study. Keep up the good work.

Weeks 1-4	Feedback phone call question for caregiver at time of call about AEs/SAEs[1]
After 2nd newsletter sent	Follow-up card with message similar to text below<br><br>Thank you for making our study a success. All of your hard work is helping us learn more about ways to improve health, and we will use this to help other families like yours. Thank you for contributing to the future and for building a healthy foundation for your family.
Weeks 5-8	Feedback phone call question for caregiver at time of call about AEs/SAEs[1]
After 3rd newsletter sent	Follow-up card with message similar to text below<br><br>Congratulations! You've made it half way. We're so proud of you for sticking with it! (refrigerator magnet included with card)
Weeks 9-12	Feedback phone call question for caregiver at time of call about AEs/SAEs[1]

[1] This table is for retention materials. For schedule for AE/SAE calls, see sub-section "Time Period and Frequency for Event Assessment and Follow-Up" within section 9.2, *Adverse Events/Serious Adverse Events.*

**Study Timeline**	
**Weeks 13-24**	**Retention Contact**
After 4th newsletter sent	Follow-up card with message similar to text below<br><br>Hi There! Keep up the good work in the iAmHealthy study. We're rooting for you!
Weeks 13-16	Feedback phone call question for caregiver at time of call about AEs/SAEs[1]
After 5th newsletter sent	Follow-up card with message similar to text below<br><br>Attention VIP (Very Important Participant)! Thanks for choosing to be healthy. Keep up the good work!
Weeks 17-20	Feedback phone call question for caregiver at time of call about AEs/SAEs[1]
After 6th newsletter sent	Follow-up card with message similar to text below<br><br>You've almost made it! The iAmHealthy study only has a few weeks to go. Don't lose steam in the last few weeks. Thanks for all you do!
Weeks 21-24	Feedback phone call question for caregiver at time of call about AEs/SAEs[1]
After post-intervention measurements complete	Touch-point card sent with message similar to text below.<br><br>Congratulations!! You've graduated from the iAmHealthy study. We're very proud of you! You'll find your iAmHealthy diploma in this package. Thank you again for participating in this study. The knowledge we've gained wouldn't be possible without you.

[1] This table is for retention materials. For schedule for AE/SAE calls, see sub-section "Time Period and Frequency for Event Assessment and Follow-Up" within section 9.2, *Adverse Events/Serious Adverse Events.*

Tracking Retention Plans

Thank-You Cards. The unblinded coordinator will send thank-you cards to participants and caregivers. The unblinded coordinator will track to whom he or she mailed the cards, as well as the mailing and delivery date. The unblinded coordinator will mail thank-you cards to participants and caregivers, as

close as possible to the schedule outlined in the Retention Plans subsection above, to keep participants and caregivers engaged while waiting for randomization and intervention.

Touch-point Cards. The unblinded coordinator will USPS mail or FedEx (or use a suitable substitute) touch-point cards to participants and caregivers, as close as possible to the schedule outlined in the Retention Plans subsection, above. The unblinded coordinator will record the date he or she mailed the card and track any responses from the participant or caregiver. The unblinded coordinator will record responses the participant or caregiver may have to the cards, and the ECHO ISPCTN site coordinator or designee, as applicable, will record any response the participant or caregiver has to the cards, if applicable.

6 TRAINING

To ensure participant safety and consistent and high-quality data submission in compliance with protocol and study operations, the DCOC and iAmHealthy study team will train ECHO ISPCTN site study staff and behavioral intervention team members. The DCOC expects all ECHO ISPCTN site study staff and behavioral intervention team members to take responsibility for learning the iAmHealthy study, primarily by studying the protocol and MOP, to ensure compliance with the protocol and GCP. The ECHO ISPCTN site study staff will receive training through the following methods: a mandatory training session in the form of an Investigator Meeting, one-on-one ECHO ISPCTN site-specific training, and other targeted training as needed. Dr. Ann Davis will host mandatory training for the behavioral intervention team on the iAmHealthy behavioral intervention.

DCOC personnel will train all ECHO ISPCTN site study staff, unblinded coordinators, and behavioral intervention team members on the protocol, reporting procedures for study deviations, potential study deviations, and noncompliance, as well as essential documentation for study conduct. The DCOC will train ECHO ISPCTN site study staff on in-person and teleconsent informed consent processes and documentation. The DCOC will train all ECHO ISPCTN site coordinators, investigators, and backups in standard in-person research anthropometric measurement techniques based upon World Health Organization procedures for assessing height and weight.[62] Previous data indicate that staff trained in these techniques have 95% reliability with an expert in their fidelity to the measurement protocol. The DCOC will also train all ECHO ISPCTN site coordinators, backups, and investigators in coaching participants in taking remote height and weight measurements, according to the instructions in the MOP. This training will include how to instruct participants on taking their own height and weight measurements.

ECHO ISPCTN site study staff and behavioral intervention team members must document all training and provide the DCOC with this training documentation. The DCOC will train the behavioral intervention team on identifying severe adverse events (AEs) and serious adverse events (SAEs) and if these events occur to refer the participant to his or her primary care physician and to inform the unblinded ECHO ISPCTN coordinator.

The DCOC will train ECHO ISPCTN site study staff and behavioral intervention team members on any relevant study electronic data capture (EDC) system(s) to ensure that staff understand how to utilize the system to perform study-specific data collection and other necessary processes.

6.1 HEIGHT AND WEIGHT INTER-RATER RELIABILITY

If not previously completed and when social distancing measures have been decreased or eliminated, blinded ECHO ISPCTN site coordinators will measure height and weight for three people at her or his site, using the instructions provided in the MOP. The backup-blinded assessor will re-measure height and weight for the same three people, as a measure of inter-rater reliability. At each re-measurement, if

the difference between initial and re-measured height and weight measurement is more than 5% (difference = [Initial – re measured]/Initial x 100), then the study team will retrain both assessors before starting/restarting the consent process.

Please see the MOP for remote height and weight measurement inter-rater reliability instructions.

6.2 CLINICAL HELPLINE AND EMAIL

If needed, ECHO ISPCTN site coordinator or investigator can call the iAmHealthy clinical help line to speak with the feasibility PI (Paul Darden, MD) or the content expert and PI (Ann Davis, PhD) regarding specific protocol questions. We will provide this phone number and a study-specific email address in the MOP.

7 STUDY INTERVENTION

7.1 CONSENT, SCREENING, BASELINE, AND POST-INTERVENTION

As outlined above, each of the four clinics will use two randomly assigned recruitment options (see Table 2). See Section 5.5, subsection *Recruitment of Participants*, for more information about the staff procedures during this part of the feasibility study. After identification of potential participants, the ECHO ISPCTN site coordinator and/or site investigator will schedule a visit, telephone call, or video chat to begin the consent process (see Section 1.3).

Consent/Assent and Other Informational Documents Provided to Participants

IN-PERSON CONSENT

Once a potential participant has agreed, ECHO ISPCTN site coordinators or research-trained designee will coordinate with clinic staff to schedule a series of days for consent/screening and baseline visits for the primary caregiver/child pair. If a potential participant is identified during a regular clinic visit that coincides with previously scheduled consent/screening or baseline visits, the ECHO ISPCTN site coordinator or a research-trained designee (from the ECHO ISPCTN site) will conduct these consent/screening/baseline activities during that visit; otherwise, the clinic nurse will schedule the potential participant for a consent/screening/baseline visit. ECHO ISPCTN site coordinators or research-trained designee will verify eligibility and conduct the consent/assent process.

TELECONSENT

Once ECHO ISPCTN site coordinator or research-trained designee assesses eligibility criteria, they will contact the potential participant to describe the study. If the participant can complete the consent/assent and/or screening processes at that time, the ECHO ISPCTN site coordinator or research-trained designee will conduct these procedures. If the participant cannot conduct these procedures at that time, the ECHO ISPCTN site coordinator or research-trained designee will schedule an appointment to conduct these procedures via teleconsent/assent and internet for screening procedures.

If a potential participant is identified during a regular clinic or telemedicine visit, the clinic nurse can give, fax, or email the participant iAmHealthy study information (e.g., study pamphlet) and can give, fax, or email the participant contact information to the ECHO ISPCTN staff. ECHO ISPCTN staff may contact

the potential participant to describe the study and, if participant agrees, complete the consent/assent process, including verifying eligibility.

INFORMED CONSENT PROCESS

ECHO ISPCTN site coordinators or research-trained designee will provide (e.g., hand deliver, postal mail, email, fax, REDCap, or electronic platform) participants consent forms describing in detail the trial intervention, trial procedures (including that study personnel will access participants' medical records, alternative contact information [as described in Participant Discontinuation/Withdrawal from the Study], and record group sessions), and risks. We will require written documentation of the informed consent prior to starting or administering the intervention. We will submit the informed consent and assent forms with this protocol.

Informed consent is a process that starts before a participant agrees to participate in the trial and continues throughout the individual's trial participation. For this trial, participants include one primary caregiver plus one child. Children meeting the reviewing IRB's requirements for signing an assent will sign the assent form. The IRB will approve consent and assent forms, and ECHO ISPCTN site coordinators or research-trained designee will ask participants to read and review the document(s). Via telecommunication or in person, the ECHO ISPCTN site coordinators or research-trained designee will explain the research trial to the participant(s) in terms the participant(s) can understand and answer any questions that may arise. The explanation will state the purposes, procedures, and potential risks of the trial and describe participants' rights as research participants. Participants will have the opportunity to carefully review the consent form and ask questions before signing. The ECHO ISPCTN site coordinator or research-trained designee will give participants the opportunity to discuss the trial with their family or surrogates or think about participating in the trial prior to agreeing to participate. The primary caregiver, and child, if applicable, can do any of the following to indicate consent/assent to the study:

1. Sign the form and hand it back to the ECHO ISPCTN site investigator or coordinator
2. Sign the form and postal mail it back to the ECHO ISPCTN site investigator or coordinator;
3. print, sign, scan, and electronically or fax return to the ECHO ISPCTN site investigator or coordinator;
4. print, sign, photograph the signature page, and text or email a picture of the signature page to the ECHO ISPCTN site investigator or coordinator;
5. type the signature and date into the electronic version of the consent document, save, and electronically return to the ECHO ISPCTN site investigator or coordinator with a note stating that this is the legal authorized representative's electronic signature. If this method is used ECHO ISPCTN site investigators or coordinators will confirm the returned consent/assent forms contain the same text as the consent/assent forms sent to the participant;
6. have the participant send a well-worded email back to the ECHO ISPCTN investigator or coordinator that states the name of the study, that the participant has read and discussed the consent document (and assent document, if applicable), and that the participant gives consent to participate.

Any of the above will be completed prior to any trial-specific procedures.

The ECHO ISPCTN site coordinator or a research-trained authorized designee will inform participants that participation is voluntary, that they may withdraw from the trial at any time without prejudice, and that nonparticipation will not adversely affect their medical care if they decline to participate in the trial. The ECHO ISPCTN site coordinator or research-trained authorized designee will ask the participant to keep a signed copy of the consent and assent forms for her or his record. Please see the iAmHealthy MOP for instructions.

ASSENT PROCESS

Assent is a process for children who have not reached the age of majority, but are old enough to understand, in general, the proposed research, its expected risks and possible benefits, and the activities expected of them as participants. The age at which participants must give assent is dependent on the IRB of record and any local site requirements for relying sites. UAMS IRB will be the IRB of record, and the age of assent is seven years old. All children who are seven years old or older must agree to be participants in the trial. Simply not disagreeing to participate does NOT constitute assent. Assent requires an expression of willingness to participate in research.

ECHO ISPCTN site coordinators or a research-trained authorized designee will conduct the informed consent process, as well as the child's assent process, via telecommunication or in person. They will document the processes in the source documents. Consent and assent documentation will include, at a minimum:

(1) the title of the trial,

(2) the date the participant entered into the trial,

(3) the name of the clinic physician or primary physician

(4) the name of the ECHO ISPCTN site investigator,

(5) the name of the person(s) obtaining the informed consent and assent, and

(6) a statement that the participant or legally authorized representative received a copy of the signed form.

The DCOC strongly recommends the following additional documentation, but it is not required:

(1) a description of how the consent/assent process occurred, including how the consent/assent form was transmitted and signed

(2) a list of who else was present during the process,

(3) a description of the type of questions asked by the participant,

(4) a list of witnesses and what they witnessed

(5) a summary of details that demonstrate the participant understood the information, and

(6) a description other specific details related to that case.

Screening and Baseline

For participants consented and screened before the enrollment pause, sites will take baseline measurements during the baseline period. Baseline measurements consist of height and weight measurements for the child and primary caregiver participants.

For participants consented after the enrollment pause, sites will conduct screening measurements during the consent/screening period. If sites cannot take screening measures during the consent/screening period, sites may take these measurements during the baseline period. Screening measurements consist of height and weight measurements for the child participant.

SCREENING

Screening will occur after consent and will include the process of confirming the participant meets all of the inclusion criteria and none of the exclusion criteria. The screening process will include height and weight measurements of the child participant to verify the participant qualifies for the study. The ECHO ISPCTN site coordinator or staff member will coach the participant, via telecommunications, in taking height and weight measurements at their home or where the participant is sheltering in place, or the ECHO ISPCTN site coordinator or staff member will take the height and weight measurements in person in the clinic. If the participants are taking measurements themselves, the ECHO ISPCTN site coordinators/investigators will watch via mobile device/computer camera. If the participant does not have Internet access, the KUCTT will loan the participant a tablet. These will be the same tablets described in Section 7.3, subsections *Acquisition and Preparation of Tablets and Selection of Tablets and Internet Providers*. Participants will receive instructions on how to take measurements, and the ECHO ISPCTN site coordinator/investigator will coach the participant in taking these measurements. ECHO ISPCTN study personnel will instruct participants to take measurements after voiding, in light clothing, and without shoes. Please see the iAmHealthy MOP for specific instructions on height and weight measurements.

Participants will take height and weight measurements on provided standardized equipment. The participant will tell the ECHO ISPCTN coordinator/investigator the measurement results, and the coordinator/investigator will enter these measurements on the appropriate CRFs. The ECHO ISPCTN site coordinator or staff member will mail or ship participants the scale and tape measure listed below or suitable replacements, approved by the iAmHealthy leadership team. Please see the iAmHealthy MOP for a complete list of equipment. If the participant qualifies for the study, the participant will keep the equipment. If the participant does not qualify for the study, the participant will return the equipment in postage-paid box that the ECHO ISPCTN site coordinator or staff member will provide.

- Etekcity Digital Body Weight Bathroom Scale With Step-On Technology (model number 025706343039)
- Amazon Basics Tape Measure (model number DS-TAM10-16ft)

If measurements are taken in the clinic, ECHO ISPCTN study staff will use the standardized equipment described in the MOP.

These measurements will not be the baseline measurements. Baseline measurements will occur at a separate time (see Baseline subsection).

ActiGraph Activity Monitor and Diet Recall. The ECHO ISPCTN site coordinator will provide participants with the activity monitor and instructions and information regarding how to schedule the 24-hour food recalls (see Section 9.1, subsection *Secondary Outcome Measures*) and instructions for completing the food recalls. Participants will complete one consent/screening/baseline three-day 24-hour food recall. The ECHO ISPCTN staff can postal mail or email the *iAmHealthy Demographics Form* (see Section 9.1, subsection *Other Measures*), link to the primary caregiver, or can ask the primary caregiver the questions via telecommunications and write the answers on the Demographics Form. This form must be completed before randomization. ECHO ISPCTN site awardee or clinic staff can obtain participant contact information to send the primary caregiver a link to the demographics questionnaire.

BASELINE MEASUREMENTS

Baseline measurements will occur after the enrollment period ends and will end before randomization. Baseline measurements will include height and weight measurements of the child and primary caregiver. An ECHO ISPCTN site staff member will either virtually coach participants in taking their own height and weight measurements or will take height and weight measurement in person. These measurements will be taken in triplicate, when feasible. The process for taking baseline measurements will be the same as the process for taking screening measurements. Please see the iAmHealthy MOP for baseline procedures instructions.

Post-intervention Measurements

For post-intervention measurements, ECHO ISPCTN site coordinators/investigators will take remote height and weight measurements in parallel with clinic-site height and weight measurements. However, if a participant cannot come to the clinic site, the participant can complete the post-intervention measurements following the same procedures for baseline measurements.

For clinic site post-intervention measurements, the ECHO ISPCTN site coordinator/investigator will use provided standardized equipment.

During the post-intervention measurements and if the equipment is used for screening or baseline measurements, the ECHO ISPCTN site coordinator will weekly assess all equipment for reliability, and he or she will conduct an accuracy check on equipment during post-intervention, and throughout the study, as necessary. The ECHO ISPCTN site coordinator will take all measures in triplicate, and the ECHO ISPCTN site coordinator will weigh the child and caregiver after they void and only in light clothing and no shoes.

Participants will also complete activity monitoring and dietary recalls at post-intervention. ECHO ISPCTN study personnel will follow the same process as outline in the Screening subsection.

7.2 RANDOMIZATION

Randomization will occur after completion of the following measures.

1. Demographics form: completion of the child's age and gender
2. Height Weight Form Baseline/Follow-up: 1 child height and weight and 1 parent height and weight
3. Diet Recall- NDSR form: 1 day of dietary recall completion

After randomization, the unblinded ECHO ISPCTN coordinator will notify child/caregiver pairs about the intervention assignment and will encourage continued participation. The ECHO ISPCTN site coordinator and a backup ECHO ISPCTN site staff member will remain blinded to intervention assignment and will perform outcome assessments.

7.3 ADMINISTRATION OF FEASIBILITY STUDY INTERVENTION

This section details the content and administration of the interventions in this trial. Please note that the ECHO ISPCTN site coordinator and the ECHO ISPCTN backup site staff member will remain blinded to intervention assignment.

Newsletter Intervention (Both Arms)

All child/caregiver pairs participating in the trial—in both the iAmHealthy behavioral intervention arm and the newsletter-only intervention arm—will receive one newsletter per month for months one through six. See Appendix A for a sample of the newsletter that we will use.

DESCRIPTION OF NEWSLETTER INTERVENTION

All newsletters used in the trial will be from the website HealthyChildren.org, a website developed and maintained by the AAP. The AAP publishes two newsletters per month that focus on child health topics, such as back-to-school illness, injury prevention, and antibiotic use. You can find the entire archive of AAP Healthy Children newsletters at: https://www.healthychildren.org/English/tips-tools/newsletters/Pages/default.aspx.

The AAP writes the newsletter at a fourth-grade reading level and designs it for the whole family. The intention is that the primary caregiver and child, if able, will read the newsletter and share/discuss relevant content with the child in the home.

ADMINISTRATION OF NEWSLETTER INTERVENTION

The unblinded ECHO ISPCTN site coordinator will send the newsletter each month to child/caregiver pairs. The unblinded ECHO ISPCTN coordinators can ship the newsletters via FedEx (or suitable replacement), or the unblinded ECHO ISPCTN coordinators can mail the newsletters via priority mail with the US Post Office, which includes tracking services.

See Section 7.4 for information regarding intervention compliance.

iAmHealthy Behavioral Intervention

In addition to the newsletter intervention, the child/caregiver pairs in the iAmHealthy behavioral intervention arm will receive the iAmHealthy behavioral intervention that includes the group and individual sessions.

Each clinic will have its own iAmHealthy behavioral intervention team that will include a fully licensed psychologist (PhD or MA) or a licensed clinical social worker with experience in weight management. These individuals will be responsible for delivering the group sessions.

Each iAmHealthy behavioral intervention team will also include a registered dietician, who will be responsible for delivering the individual sessions and co-facilitating the group sessions (see Section 7.2, subsections *Individual Sessions* and *Group Sessions*).

DESCRIPTION OF THE IAMHEALTHY BEHAVIORAL INTERVENTION

All participants randomized to the iAmHealthy behavioral intervention arm will receive this intervention, composed of a previously developed, participant tailored, telemedicine intervention that is family based and provides information on the following behavioral, nutritional, and physical activity topics.[19,59,63,64]

- Goal setting and sticker charts
- Reading food labels
- Stop Light Diet
- Energy balance
- Primary caregiver health behavior changes
- Use of privileges (trip to library, family walk)

- Decreasing sedentary activity and increasing physical activity for caregivers and children
- Self-esteem
- Family exercise ideas
- Cooking with a limited budget
- Praising and ignoring
- Making healthy choices
- Parties, barbeques, and eating out
- Monitoring screen time
- Nutrient density
- Portion sizes
- Healthy foods in the home

The iAmHealthy behavioral intervention content is founded on cognitive behavioral theory[65] and child weight theory of Davison and Birch.[66] The topics of the iAmHealthy behavioral intervention include all child and caregiver behaviors/characteristics from child weight theory[67] and those identified as key in previous qualitative research with rural parents.[64] The iAmHealthy behavioral intervention builds on previous research by delivering this intervention to families in their homes (see Section 4.3 for a detailed description of the significance and innovation of this intervention).

The iAmHealthy behavioral intervention is composed of 26 contact hours: 15 hours of group sessions and 11 hours of individual sessions (see Section 4.1).

CONTENT OF IAMHEALTHY BEHAVIORAL INTERVENTION

The content of the iAmHealthy behavioral intervention focuses on behavioral, nutritional, and exercise topics and contains both activities and didactic lessons. These topics are discussed at the individual, family, and community level, allowing families to discuss barriers and facilitators unique to their rural communities and to develop social connectivity among participants. Topics covered include the following:

- Decreased focus on fast food/junk food
- Increased focus on healthy eating at social gatherings
- Increased information on exercise activities for children that can be done alone (due to poor proximity to neighbors, etc.)
- Resources and support available in the community
- Increased attention to self-esteem

The behaviorial intervention team will make dietary and physical activity recommendations to the child/caregiver pairs during the group (see Section 7.2, subsection *Group Sessions*) and individual sessions (see Section 7.2, subsection *Individual Sessions*). Child/caregiver pairs will be taught meal preparation and exercise ideas as part of the intervention. Each iAmHealthy behavioral intervention team will refer child/caregiver pairs to tools that are available online, and the group sessions will allow child/caregiver pairs to share tools and activities available in their communities.

ADMINISTRATION OF THE IAMHEALTHY BEHAVIORAL INTERVENTION

The iAmHealthy behavioral intervention teams will work with the participating child/caregiver pairs to deliver the iAmHealthy behavioral intervention using video conference and a tablet computer.

Both members of the behavioral intervention team will be experienced in pediatric obesity intervention work and will receive additional training specific to the iAmHealthy behavioral intervention. Both behavioral intervention team members will follow the iAmHealthy intervention manual and will receive

four to eight hours of training with Dr. Davis, PI, prior to the start of their intervention groups. This training will focus on proper delivery of the treatment groups via video conference. Treatment sessions will focus on making the groups fun and getting caregivers and children interacting and involved during the sessions.

We will centrally engage and hire behavioral interventionists and assign them to cohorts based upon a variety of factors, including familiarity with the population and coordination of availability. This central model of behavioral intervention team engagement greatly increases feasibility and efficiency of the iAmHealthy behavioral intervention. Of note, we have engaged the appropriate number of experienced, ethnically appropriate interventionists for implementation of the current proposed trial.

For more information regarding trial intervention compliance, see Section 7.4.

GROUP SESSIONS

The iAmHealthy behavioral intervention period is six months. This includes weekly group sessions for the first three months, followed by monthly group sessions for three months (Table 3 and Schedule of Activities, Section 1.3). Each child/caregiver pair consented from the same clinic will attend each group session.

Table 3. Intervention Timeline

Intervention Period:		Months 1-3	Month 4	Month 5	Month 6
iAmHealthy Intervention	Group sessions	12 weekly sessions	1 session	1 session	1 session
	Individual sessions	11 hours of homework help via remote technology			

The iAmHealthy behavioral intervention psychologist/social worker and dietician will deliver one-hour group sessions via video conferencing technology on the tablet to child/caregiver pairs at times convenient for the child/caregiver pairs within the group (typically evenings and weekends). Previous work using these modalities shows feasibility for finding one hour during the week (evening or weekend) that accomodates all participating child/caregiver pairs.[59,63] If participating child/caregiver pair miss a group session, the pair will receive a makeup phone call from the behavioral intervention dietician. The dietician will review all content that was covered in the missed group session. (see Section 8.2 for information on participant discontinuation and/or withdrawal). This makeup session will count as a regularly attended session, and the dietician will document the attendance and contents covered in the makeup session.

Each session will begin with a review of progress since the last meeting and will end with primary caregivers and children setting goals. Primary caregivers and children will work together throughout each session, and both must be present for the entire meeting.

INDIVIDUAL SESSIONS

The iAmHealthy behavioral intervention also includes 11 hours of individual sessions (or “homework”) for each participating child/caregiver pair that the pair must complete during the six months of the trial. The iAmHealthy behavioral intervention dietician will flexibly schedule the individual sessions and the content will be the same as outlined above. Based on individual pace, the homework time may exceed 11 hours.

We will preload the individual session assignments onto the tablet and each child/caregiver pair will complete the session with the iAmHealthy behavioral intervention dietician. For example, after the Week 3 Stop Light Diet lesson, the iAmHealthy behavioral intervention dietician will ask child/caregiver pairs to identify all of the food items in their cabinets/refrigerators as “red,” “yellow,” or “green” by using stickers mailed to the participant prior to this lesson. “Red” foods are food items with ≥7 grams of

fat and/or ≥12 grams of sugar per serving. The iAmHealthy behavioral intervention dietician will encourage child/caregiver pairs to eat fewer "red" food items and more "green" food items (fruits and vegetables with nothing added) and to eat "yellow" food items (foods that are not green but do not meet the threshold for "red") in moderation. The iAmHealthy behavioral intervention dietician will be able to help each individual child/caregiver pair complete this homework activity through the video conferencing technology, actually looking into the child/caregiver pair's cabinets with them and helping them to appropriately categorize foods according to their labels.

The culturally competent registered dietician will work with each child/caregiver pair during the individual sessions to tailor any content to their family, ethnic, or cultural beliefs, and/or their community, as needed. Examples might include how to exercise in religious garments or how to cook without meat. The iAmHealthy behavioral intervention dietician and child/caregiver pairs will discuss common behavioral concepts (e.g., goal setting, problem solving, reinforcement, and tracking) in their individual sessions, and iAmHealthy behavioral intervention psychologist/social worker will also teach these same concepts in the group sessions.

We have tailored all materials to the targeted age group in this intervention. The goal is that the child and caregiver will complete these assignments together, with the help of the iAmHealthy behavioral intervention dietician. If a child/caregiver pair misses an individual session, it will be rescheduled at a more convenient time.

Acquisition and Preparation of Tablets

The KUCTT will purchase and format tablets for the trial. Once KUCTT properly formats the tablets, KUCTT will ship two tablets to each ECHO ISPCTN site for the ECHO ISPCTN site coordinators to use during consent/screening/baseline measures and during the post-intervention measures. KUCTT will also ship the appropriate number of tablets (one tablet for each family in the iAmHealthy behavioral intervention arm) to the ECHO ISPCTN unblinded coordinator, who will distribute them to the appropriate child/caregiver pairs. If a participating child/caregiver pair loses the tablet or if a tablet breaks or malfunctions, KUCTT or the DCOC will replace it.

The iAmHealthy behavioral intervention team will not receive tablets. They will connect to the group and individual sessions through their computer or mobile device.

KUCTT will format the tablets to ensure that no PHI is stored on the tablet. KUCTT will preload the tablets with the intervention manual, the video conferencing software, recommended applications, and other trial-related educational materials.

Once the intervention period is complete, ECHO ISPCTN site coordinators will instruct participants to return the tablets to KUCTT via the prepaid shipping materials that participants will receive with the tablet. All tablets will be "cleaned" of information after the trial, and they will be available for future ECHO ISPCTN needs. The KUCTT technology team has proven strategies for tablet retrieval and tracking that it has used in several other NIH-funded protocols.

Selection of Tablets and Internet Providers

The technology team will select tablets that include the most efficient technology and functionality for this trial-related software, as well as a strong case with a stand feature for the tablet. The technology team will load the tablets with fully functional unlimited cellular data plans, selected by the technology team to be the optimal provider for the use area.

The technology team will select Internet providers based on high-quality coverage in the specific region where participants live. In past studies, the technology team has obtained high-quality video conferencing streaming service in 24 rural areas. If child/caregiver pairs encounter difficulties, as a back-up plan, they will be able to call into the meeting via a toll-free telephone number. Additionally, technical support will be available, via one-on-one teleconference or telephone call.

Tablet Delivery and Storage

Once tablets are prepared, KUCTT will ship them to the unblinded ECHO ISPCTN coordinators, who will store them in a locked cabinet until shipped to the participants in the iAmHealthy behavioral intervention arm. The unblinded ECHO ISPCTN coordinators will be responsible for tracking tablets via a paper log and for contacting the KUCTT team if any equipment or usage problems arise.

7.4 MEASURES TO MINIMIZE BIAS: RANDOMIZATION AND BLINDING

ECHO ISPCTN site awardees will recruit clinics, and each of those clinics will randomize up to 32 child/caregiver pairs. Once pairs complete the consent/assent (if appropriate) processes and all baseline assessments, the ECHO ISPCTN site coordinator will note this in the EDC. Once all pairs complete the consent/assent processes and baseline assessments, the DCOC will randomize participants. The DCOC will randomize all child/caregiver pairs into the interventions at one time. The DCOC will inform the unblinded ECHO ISPCTN coordinator of the intervention assignments, who will notify child/caregiver pairs and distribute tablets as necessary.

The ECHO ISPCTN site coordinator will remain blinded and serve as the blinded assessor. The ECHO ISPCTN site investigator will also designate a research-trained blinded backup coordinator from the ECHO ISPCTN site. The blinded coordinators will not have access to clinic charts. The unblinded ECHO ISPCTN coordinator will ask participants and their caregiver not to disclose their intervention assignment to or discuss previous weights with the blinded ECHO ISPCTN site coordinator. The blinded ECHO ISPCTN site coordinator, or the designated backup, will take the height and weight measurements at consent/screening/baseline and post-intervention, and the study team will use these measurements to evaluate the secondary endpoints. In case the ECHO ISPCTN site coordinator is accidentally unblinded, the backup assessor will take over all assessments for that participant.

At each measurement time point, the blinded assessor will enter data into the EDC system for that time point. A member of the Kansas dietetics and nutrition team will perform the 24-hour food recalls at consent/screening/baseline and post-intervention.

Table 4. Blinded and Unblinded Personnel

Blinded	Unblinded
ECHO ISPCTN Site coordinator	Clinic nurse
Backup site staff member	Clinic provider
ECHO ISPCTN site investigator	iAmHealthy intervention team
Medical monitor*	Statistician
Dietetics and nutrition team	Unblinded ECHO ISPCTN coordinator
*Can be unblinded for safety reasons	

7.5 STUDY INTERVENTION COMPLIANCE

The iAmHealthy behavioral intervention psychologist/social worker will be responsible for delivering the group sessions with assistance from the dietician, and the iAmHealthy behavioral intervention dietician will be responsible for delivering the individual sessions for the iAmHealthy behavioral intervention arm

(see Sections 7.2, subsection *Administration of the iAmHealthy Behavioral Intervention*). To ensure compliance, we will assess fidelity to the group and individual sessions of the iAmHealthy behavioral intervention through fidelity checklists that each iAmHealthy behavioral intervention team leader will keep. The iAmHealthy behavioral intervention team will use fidelity checklists during iAmHealthy behavioral interventions to ensure that they cover the appropriate topics. The developed checklists include 6 to 10 major topics for each group session, and the iAmHealthy behavioral intervention team member will check off each topic as he or she covers it during the session. At the end of the session, a member of the iAmHealthy behavioral intervention team will record attendance and topics covered using an EDC system.

The iAmHealthy behavioral intervention teams will receive weekly supervision from a licensed clinical psychologist (i.e., supervising psychologist) who has experience delivering pediatric obesity intervention programs. The supervising psychologist will review digital video recordings of group sessions and provide feedback to the intervention teams. If participants divulge any unanticipated psychological or medical issues, these team supervision meetings will allow for general clinical supervision.

The supervising psychologist will review 25% of all sessions and compare them to the developed fidelity checklists. To assess for reliability, a second independent coder (an alternate supervising psychologist) will code 50% of the already coded sessions by using the same fidelity checklist.

In addition, the iAmHealthy behavioral intervention teams will participate in a weekly meeting with Dr. Ann Davis, PI, to discuss fidelity findings and to retrain as necessary.

We will assess fidelity to the newsletter intervention on a post-trial questionnaire (see Section 9.1, subsection *Other Measures*) in which study personnel will ask caregivers about the amount of time they spent reading each newsletter.

7.6 CONCOMITANT THERAPY

We will allow child/caregiver pairs to continue any concomitant medications or therapies for which they are receiving treatment.

A post-trial questionnaire (see Section 9.1, subsection *Other Measures*) will ask child/caregiver pairs about concomitant therapies they used during the intervention period.

8 STUDY INTERVENTION DISCONTINUATION AND PARTICIPANT DISCONTINUATION/WITHDRAWAL

8.1 DISCONTINUATION OF STUDY INTERVENTION

Since this is a behavioral intervention trial, the trial team does not expect any intervention-related safety events causing the investigator, the medical monitor, the Data Safety Monitoring Board (DSMB), or the IRB to suspend or stop the trial. However, there could be events related to emotional distress from answering questions regarding nutrition and physical activity.

If a child or caregiver within any clinic has an intervention-related SAE (see Section 9.2), the ECHO ISPCTN site team will review or modify the trial, with input from the DSMB, assessing impact on risk.

Consented child/caregiver pairs will continue all remaining trial measurement procedures, as indicated in the trial protocol. If ECHO ISPCTN site awardees or clinic sites identify a clinically significant finding after the intervention period begins, the ECHO ISPCTN site investigator or qualified designee will determine if the site needs to change any aspect of participant management. We will report any new clinically significant finding as a severe AE.

The ECHO ISPCTN site investigator will determine if a severe AE or SAE is clinically significant, with input from the DSMB and/or medical monitor, as appropriate. If a severe AE or SAE is a clinically significant event, we will specifically monitor for this event, as specified in the MOP, (as well as any event for which the child seeks additional medical care [e.g., emergency department visits or hospitalizations]). Please see the MOP for the definition of clinically significant.

8.2 PARTICIPANT DISCONTINUATION/WITHDRAWAL FROM THE STUDY

A child/caregiver pair may withdraw from the trial at her or his own request, or the primary caregiver, site investigator, DSMB, and/or DCOC can withdraw a child/caregiver. If a child/caregiver pair withdraws from the study, ECHO ISPCTN site coordinators, investigators, or unblinded ECHO ISPCTN coordinators can ask the child/caregiver pair why they chose to withdraw. The pair can choose not to answer this question. Reasons for withdrawal include:

- Child/caregiver pair is unwilling or becomes unable to comply with the assessment procedures required by the protocol.
- Child/caregiver pair is withdrawn for safety reasons (e.g., occurrence of a severe AE).
- Child/caregiver pair meet any exclusion criteria and no longer meet the inclusion criteria, with the exception of BMI.

If a child/caregiver pair no longer receives care at the clinic at which they are consented, the study team will make every attempt to retain the participant. ECHO ISPCTN site personnel will record the reasons for withdrawal in the trial documentation.

Clinic sites can replace child/caregiver pairs if they withdraw prior to the start of the intervention; however, clinic sites will not replace child/caregiver pairs who withdraw after the start of the intervention.

While it is unlikely, a child’s primary caregiver could change during the trial. If this occurs, we will unblind the ECHO ISPCTN site coordinator (and the backup assessor will take over all assessments for that participant [see Section 7.3]), and the ECHO ISPCTN site coordinator will contact the new primary caregiver to seek their participation, according to applicable local laws. If the new primary caregiver chooses to consent, the ECHO ISPCTN site coordinator will remove the original caregiver’s BMI data from analyses, but will keep the child’s data and both the new caregiver and child will continue to participate. If the new primary caregiver does not consent, the participant will be withdrawn.

To allow for child/caregiver pairs who do not complete the trial, clinic sites will consent up to 32 child/caregiver pairs, per clinic, with only 28 child/caregiver pairs needed for a cohort (i.e., each ECHO ISPCTN site will consent 28 participants for randomization). We will consider a child/caregiver pair lost to follow-up if the clinic team, ECHO ISPCTN site team, or PIs are unable to contact the pair.

9 STUDY ASSESSMENTS AND PROCEDURES

9.1 EFFICACY ASSESSMENTS

The following section outlines the assessments and assessment-related procedures. Please note that the ECHO ISPCTN site coordinator will remain blinded, but we will not blind the clinic nurse.

The unit of measurement will be at the participant level. Further details will be included in the statistical analysis plan (SAP).

Primary Outcome Measures

Objective 1 – Participant Recruitment Rate. For each recruitment option, recruitment will occur over four weeks (Table 2). There will be three numbers collected: potential participants, potential participants approached, and participants randomized. We will measure participant recruitment rate for both recruitment options. The numerator will be the number of participants randomized, and the denominator will be the number of participants approached. Potential participants for recruitment option 1 will be the number of patients seen in the last year, who meet inclusion criteria (retrospective), and the number of patients seen during the four-week recruitment period, who meet inclusion criteria (prospective). We do not define the potential participants for recruitment option 2. For recruitment option 1, the number for potential participants approached will be the number of potential participants approached by ECHO ISPCTN site coordinator. For recruitment option 2, the potential participants approached will be the number of self-referred potential participants. We will obtain the number of potential participants and potential participants approached from a list maintained by the ECHO ISPCTN site coordinator with the assistance of clinical staff, as needed (see Section 5.5, subsection *Recruitment Option 1*). ECHO ISPCTN research staff will enter these numbers into an EDC system during the recruitment period. We will also discuss the data during the weekly study-team calls. Please see Figures 3 and 4 for a graphical representation of these numbers.

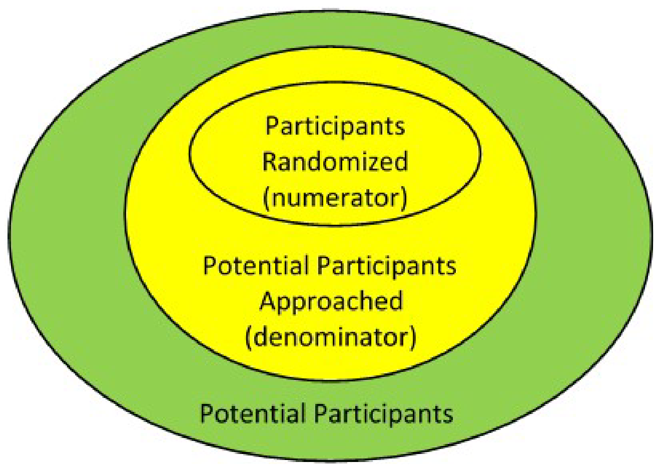

Figure 3. Participant Recruitment Overview in each Clinic

Objective 2 – Participant Retention. We will calculate participant retention as the percent of randomized participants who remain in the study through the final measurement time point. The numerator will be the number of randomized participants who complete the final measurement time point and the denominator will be the total number of randomized participants. We will then calculate the retention rate for each clinic site and by treatment arm across all clinic sites.

Objective 3 – Dose. Among participants randomized to the iAmHealthy behavioral intervention arm who are retained through the final measurement, we will calculate the proportion of the participants who receive at least 80% of the planned intervention (dose). The planned dose is 26 contact hours, and 80% of the planned dose is 20.8 planned contact hours. The iAmHealthy behavioral intervention psychologist/social worker will enter into an EDC system participant attendance at each group session, and the iAmHealthy behavioral intervention dietician will enter into an EDC system participant attendance at each individual session. The total number of sessions possible will serve as the denominator and the total number of sessions attended will serve as the numerator; we will calculate this dose rate for each child/caregiver pair.

Objective 4 – Staff Blinding. We will assess blinding with the New Blinding Index.[2,3] This index estimates the percentage of correct guessing beyond the level expected by chance in each treatment arm. Specifically, the ECHO ISPCTN site coordinator and a designated backup will remain blinded and serve as the blinded assessor. We will ask the blinded assessor at the time of the final measurements to enter into an EDC system her or his estimation of the treatment assignment for each participant. The options

for assessors to select from will include treatment, control, and do not know (options required by the New Blinding Index).

Secondary Outcome Measures

1. **Child and Primary Caregiver Body Mass Index (BMI) and Child BMI adjusted z-score (BMIaz)**. These measures are associated with Secondary Objectives 1 through 3. Although there has been debate about the best measure of childhood adiposity,[68] the recently released USPSTF guidelines indicate that BMI is a widely used metric for pediatric treatment outcome research. [28] Among adults, BMI is clearly the preferred and most widely used metric of adiposity in large-scale treatment outcome research. BMI is a measure of adiposity that is also highly feasible, based primarily on the measurement of height and weight. To calculate BMI for both children and adults, ECHO ISPCTN site coordinators and/or site investigators will use standard formulas/equations.

   As a secondary measure, we will use BMIaz to measure childhood adiposity change. BMIaz is a recently developed calculation that is designed to be more sensitive to change for children over the 95th percentile.[1] A comparison between BMI and BMIaz in this large and well-studied sample will be a valuable addition to the literature.

2. **24-hour Food Recall.** This measure is associated with Secondary Objective 4. The 24-hour food recall is a standardized five-pass method developed by the US Department of Agriculture for use in national dietary surveillance. Although there are weaknesses to every method of dietary assessment, we selected this method because several large trials, which served as models for the current trial, used it.[69,70-72] Data suggest that the 24-hour food recall is the most valid and reliable method of dietary assessment for children.[73]

   All child participants will complete the 24-hour food recall at consent/screening/baseline and at post-intervention. For each 24-hour food recall assessment period, a member of the Dietetics and nutrition team will collect data via telecommunications from child/caregiver pairs for two weekdays (i.e., two days during a Monday through Friday time period) and one weekend day (i.e., one day during a Saturday and Sunday time period) , as is recommended.[73] Regarding school lunches eaten away from home, per typical procedures, caregivers will review the school lunch menu with their child at the end of each day and have the child report, to the best of his or her ability, what he or she consumed for lunch at school that day.

   We will analyze recalls with the Nutritional Data System for Research (version 2018; University of Minnesota, Minneapolis, MN). Although much information is available from this analysis, the current trial will specifically focus on the number of servings of sugar-sweetened beverages per day, the number of "red" food items per day (foods with ≥7 grams of fat and/or ≥12 grams of sugar per serving), and the number of servings of fruits and vegetables per day. (For more information on "red" food items as part of the Stop Light Diet, see Section 7.2, subsection *Individual Sessions*).

   An expert in dietary training will train all staff completing dietary recalls to collect the 24-hour food recall data using highly standardized probes, and we will require the staff to meet testing requirements before we allow them to collect the baseline 24-hour food recall.

3. **ActiGraph Physical Activity Monitors.** This measure is associated with Secondary Objective 5. We will measure physical activity by using the ActiGraph physical activity monitor (ActiGraph LLC, Pensacola, FL), a small, lightweight activity monitor that is worn on an adjustable belt over the hip contralateral to the dominant hand, allowing objective measurement of physical activity. The

monitor measures start time and data collection intervals (one-second epochs). The ActiGraph has a filter that rejects motion outside normal human movements. The ActiGraph has been shown to provide valid assessments of physical activity for adults and children during both laboratory activities (treadmill walking/running) [74,75] and daily living activities.[76-78]

All child participants will complete physical activity monitoring at consent/screening/baseline and at post-intervention, and we will ask child participants to wear the monitor on the same hip during the consent/screening/baseline and post-intervention monitoring phases. For each monitoring point (consent/screening/baseline and post-intervention), participants will receive detailed instructions on wearing and caring for the monitor, and ECHO ISPCTN site coordinator or investigator will ask them to wear the monitor for a maximum of seven consecutive days and a minimum of four days during a one week period. For analyses, we will consider at least eight hours of wear time in a 24-hour period a "valid" day (consistent with previous research).[79] Participants will wear monitors during waking hours, excluding water-based activities (i.e., swimming, bathing). We will provide postage-paid mailing supplies for the caregiver to mail, via priority mail, the activity monitor to the ECHO ISPCTN site coordinator or investigator. The site coordinator or investigator will be responsible for retrieving data for analysis by downloading the data to a computer via a USB port. If a participating family loses the activity monitor or if an activity monitor breaks or malfunctions, ECHO ISPCTN site or DCOC will replace the activity monitor.

The DCOC/ISPCTN sites will process all data with ActiLife software, which accounts for age and gender cut-offs.[80] We will then normalize these data for wear time. The primary outcome will be average time spent per day in moderate to vigorous physical activity across valid wear days, as well as the percent of time spent in moderate to vigorous physical activity. We will assess the ActiGraph monitors for proper functioning before use and between child/caregiver pairs.

Tertiary Outcomes

1. Assess the reasons that parents decline to participate in the iAmHealthy trial and whether those reasons differ by recruitment option or by clinic. Specifically, ECHO ISPCTN site coordinators/investigators will ask every family who declines to participate for the primary reason for their negative response. We will code and categorize those reasons by recruitment option and clinic, as well as across recruitment options and across clinic.
2. Assess recruitment options in more detail by determining the total number of participants randomized via each recruitment option at each clinic, as well as whether each clinic met its recruitment goal of at least eight randomized participants via each recruitment option. Specifically, in addition to recruitment rate (Objective 1) study staff will track the total number of participants randomized via each recruitment option at each clinic, as well as a dichotomous variable of whether or not the clinic met its overall goal by and across recruitment option.

Other Measures

Other measures for this trial include a demographics questionnaire (*iAmHealthy Demographics Form*), the *Post-Trial Questionnaire*, and a technology feasibility log. We will conduct the following measures via an EDC link, telecommunications, or postal or electronically mailing the measures to participants, ask them to complete and return to the consenting ECHO ISPCTN site coordinator or research-trained backup.

Demographics. This questionnaire (*iAmHealthy Demographics Form*) will ask about age, race, ethnicity, household income, Medicaid/insurance status, caregiver education, and zip code (to determine rurality

using RUCA codes). Additionally, this questionnaire will assess the availability of home computers, high-speed Internet access, tablets, smartphones, or other technology already present in the home that participants could use for secure videoconference connections in future studies. In addition, this questionnaire will ask participating caregivers how they heard about the iAmHealthy project and if the caregiver was approached through other recruitment options. Participating caregivers will complete this questionnaire before randomization.

Post-Trial Questionnaire. To assess for possible contamination effects and to assess use of the newsletter, caregivers will complete a short questionnaire asking about any contact they had with their primary care physician during the intervention period, as well as other weight loss efforts or health behavior change efforts in which the caregiver or the child engaged. In addition, we will ask participants to record receipt of the newsletter, how often they read the newsletter, amount of time reading the newsletter, and helpfulness in implementing weight loss efforts. Participating caregivers will complete this questionnaire during the post-intervention period.

Technology Feasibility. Individual and group session leaders will keep technology problem logs to record difficulties they have 1) connecting to the child/caregiver pairs for the sessions or 2) with technology interruptions during sessions. Previous research indicates these problems are rare, but the degree of these types of problems when delivering interventions to the home is unknown.

As a second measure of technology feasibility, participating caregivers will complete the questionnaire during the post-intervention time point.

Study Personnel Time Assessment. During the recruitment phase, the DCOC will weekly send a time assessment survey and ask all study personnel (ECHO ISPCTN study personnel, unblinded ECHO ISPCTN coordinator, and clinic nurse) to report the time spent on different trial activities during that week. At the end of the feasibility study, we will assess these time logs to determine the actual time study personnel devoted to the trial activities.

Study Personnel Satisfaction Survey. The DCOC will ask all study personnel to complete a brief satisfaction survey shortly after the recruitment period ends and after the final measurements are completed.

9.2 ADVERSE EVENTS/SERIOUS ADVERSE EVENTS

Definition of Adverse Events

An AE is defined as any untoward occurrence associated with the use of an intervention in humans, whether or not considered intervention related (21 CFR 312.32 [a]). We include a list of potential AEs; however, we will record and track SAEs, per the definition below. We will only record severe AEs.

Definition of Serious Adverse Events

We will consider an AE or suspected adverse reaction "serious" if, in the view of the PIs, medical monitor, DSMB, or sponsor, it results in any of the following outcomes:

- Death
- Life-threatening AE
- Inpatient hospitalization or prolongation of existing hospitalization
- Persistent or significant incapacitation or substantial disruption of the ability to conduct normal life functions

- Congenital anomaly/birth defect.

Important medical events that may not result in death, be life-threatening, or require hospitalization may be considered serious when, based upon appropriate medical judgment, they may jeopardize the participant and may require medical or surgical intervention to prevent one of the outcomes listed in the SAE definition. Examples of such medical events include allergic bronchospasm requiring intensive treatment in an emergency room or at home, blood dyscrasias, or convulsions that do not result in inpatient hospitalization, or the development of drug dependency or drug abuse.

Classification of an Adverse Event

SEVERITY OF EVENT

These are the guidelines to describe the severity of AEs:

Mild. Events require minimal or no treatment and do not interfere with the participant's daily activities.

Moderate. Events result in a low level of inconvenience or concern with the therapeutic measures. Moderate events may cause some interference with daily functioning.

Severe. Events interrupt a participant's usual daily activity and may require systemic drug therapy or other treatment. Severe events are usually potentially life threatening or incapacitating. Of note, the term "severe" does not necessarily equate to "serious."

RELATIONSHIP TO TRIAL INTERVENTION

All severe AEs and SAEs must have their relationship to trial intervention assessed by the clinician who examines and evaluates the participant based on temporal relationship and her/his clinical judgment. We will grade the degree of certainty about causality by using the categories below.

Related. We know the AE occurred with the trial intervention, there is a reasonable possibility that the trial intervention caused the AE, or there is a temporal relationship between the trial intervention and event. Reasonable possibility means that there is evidence to suggest a causal relationship between the trial intervention and the AE.

Not Related. There is not a reasonable possibility that the administration of the trial intervention caused the event, there is no temporal relationship between the trial intervention and event onset, or there is an established alternate etiology.

EXPECTEDNESS

The ECHO ISPCTN site investigators will be responsible for determining whether a severe AE or SAE is expected or unexpected. A severe AE or SAE will be considered unexpected if the nature, severity, or frequency of the event is not consistent with the risk information previously described for the trial intervention.

Of note, the evidence report and systematic review for the USPSTF addressing screening for obesity and intervention for weight management in children and adolescents did not report any SAEs among 10 lifestyle-based intervention trials.[28] Potential events could include the following.

- Distress from being identified as overweight or obese
- Conflict between parent or family and child related to
    - Recommendations for exercise
    - Recommendations for change in food buying and/or dietary changes

- Increased body image concerns
- Discomfort in social settings where recommended healthy diet may be in conflict with food served
- Conflict with peers related to recommendations for a healthy diet
- Soreness related to increased activity
- Injury related to increased activity

Time Period and Frequency for Event Assessment and Follow-Up

While not expected, a participant may experience psychological and/or behavioral difficulties during the trial. If this occurs, the caregiver may contact the group psychologist or clinical social worker to discuss these issues (if the participant is in the iAmHealthy behavioral intervention arm) or contact the ECHO ISPCTN unblinded coordinator (if the participant is in the newsletter only arm). If the participant is in need of psychological care, a behavioral intervention team member or the operational PI, depending on the issue, will ask the parent to contact the participant's primary care physician for referral, or other support as needed. If the group psychologist, clinical social worker, operational PI suspects that a severe AE or SAE has occurred, he or she will contact the unblinded ECHO ISPCTN coordinator. If the unblinded ECHO ISPCTN coordinator determines that a severe AE or SAE has occurred, he or she will enter this into the EDC system.

Beginning on Week 1 of the intervention period, the unblinded study coordinator will call participants approximately monthly to inquire about severe AEs and SAEs. The occurrence of a severe AE or SAE may also come to the attention of trial personnel (e.g., ECHO ISPCTN site study personnel or behavioral intervention team members) during contact with a trial participant (child or primary caregiver). When trial personnel learn of a severe AE or SAE, he or she will refer the participant to his or her primary care physician. The trial personnel learning of the severe AE or SAE will also inform the unblinded ECHO ISPCTN coordinator, who will record the severe AE or SAE into the EDC and will track the SAE.

The unblinded ECHO ISPCTN coordinator will record severe AEs, as described in the next section and in the MOP. Severe AE data collection typically includes event description, time of onset, if available, resolution time and day, if available, and ECHO ISPCTN site investigator's assessment of severity, relationship to study participation or intervention (assessed only by those with the training and authority to make a diagnosis), and time of resolution/stabilization of the event. The unblinded ECHO ISPCTN coordinator must appropriately document severe AEs occurring during the trial, regardless of relationship to the trial. The DCOC safety personnel will track severe AEs until one of the following criteria are met: resolution, the condition stabilizes, the event is otherwise explained or is judged by the ECHO ISPCTN site investigator to be no longer clinically significant, or the participant is withdrawn from the study. DCOC safety personnel will follow all SAEs by asking the unblinded coordinator about the SAE every 14 days until resolution or until the ECHO ISPCTN site investigator deems the event to be chronic, or until the participant is stable. The DCOC may request other supporting documentation of the event and should be provided as soon as possible.

We will not record any medical condition that is present when the ECHO ISPCTN site coordinator screens the participant at consent/screening/baseline as an AE. However, if the trial participant's condition deteriorates at any time during the trial to meet the definition of a severe AE, the unblinded ECHO ISPCTN coordinator will record this.

The unblinded ECHO ISPCTN coordinator will document changes in a severe AE in the source document and EDC to allow an assessment of the duration of the event. Severe AEs characterized as intermittent require documentation of onset and duration of each episode.

The unblinded ECHO ISPCTN coordinator will inquire about severe AEs and SAEs via monthly (approximately) participant phone calls and will record all reportable events that occur from Week 1 of the intervention period. Participants may report severe AEs for seven days post-intervention and SAEs for 30 days post-intervention. Unblinded coordinators and/or DCOC safety personnel will record and track according to this protocol and DCOC standard operating procedures.

Adverse Event Reporting

We will record severe AEs in the EDC system, include them in the statistical analysis, and follow the reporting procedures outlined in the trial-specific MOP. The MOP section on reporting severe AEs will encompass the requirements of:

1. Reviewing IRB's policies and procedures
2. Standard Operating Procedures (SOPs) for the Streamlined, Multisite, Accelerated Resources for Trials IRB Reliance platform (SMART IRB)
3. ICH E6(R2), Good Clinical Practice: Integrated Addendum to ICH E6(R1): Guidance for Industry
4. Following local IRB's policies and procedures, when applicable. If there are discrepancies between the procedures, we will follow the most stringent of the procedures.

We will yearly report severe AEs to the reviewing IRB for its continuing review. The lead trial team will report to the Central IRB, as specified in the study-specific IRB communication plan, which is based on recommendations of SMART IRB. We will also report severe AEs to the local IRB, per local IRB policies and procedures, and make them available to the sponsor (DCOC) on a continuing basis through the EDC system. We will report to the DSMB, per the DSMB charter.

The trial-specific MOP will describe the details of the reporting structure and additional details related to timelines for reporting.

The unblinded ECHO ISPCTN coordinator will record severe AEs and SAEs into the EDC, as described in the MOP. The record must include, at a minimum, the following information: 1) description of the severe AE and/or SAE, 2) onset time and day of the severe AE, if available, and 3) resolution time and day of the severe AE, if available. The unblinded ECHO ISPCTN coordinator must enter all severe AEs and SAEs on a severe AE and log.

The specific regulations of the Food and Drug Administration (FDA) do not apply to this protocol because this is not an FDA-regulated trial.

Serious Adverse Event Reporting

The unblinded ECHO ISPCTN coordinator will record all SAEs in the EDC, and the DCOC will report SAEs, per Table 5, below. The DCOC will notify monitoring personnel (e.g., medical monitor) and monitoring bodies (e.g., DSMB), per the DSMB charter. The DCOC will make these reports whether or not the trial clinician or medical monitor consider the event trial-intervention related; reports must include an assessment of whether there is a reasonable possibility that the trial intervention caused the event.

Table 5. Serious Adverse Event Reporting

	Report to	Timing	Notes
1	Reviewing IRB (SMART IRB definition)	Yearly (minimum)/at continuing review	For continuing review<br>Submitted to IRB via DCOC
2	Local IRB	Per local IRB policies and procedures	

3	DCOC (Sponsor, per SMART IRB definitions, and lead trial team)	Immediately, but not later than 48 hours after finding out about the event	CO-SOP-012.v1.0/SOP
4	Overall PIs (per SMART IRB definitions)	Immediately, but not later than 24 hours after finding out about the event	
5	Medical Monitor	Per DSMB charter	
6	DSMB	Per DSMB charter	

DCOC safety personnel will track all SAEs until satisfactorily resolved or the ECHO ISPCTN site investigator deems the event is chronic or the participant is stable. The DCOC/trial sponsor may request other supporting documentation, and sites should provide this as soon as possible.

The DCOC will ensure the summary and report of SAE data to the reviewing IRB in time for consideration at the next continuing review. The DCOC will also create and provide any SAE data summaries requested by the monitors or specified in the DSMB charter. The ECHO ISPCTN site investigator will be responsible for following their local institution's requirements.

The trial-specific MOP has additional details.

Reporting Events to Participants

We will notify participants of those trial-related (or potentially trial-related) SAEs that may affect their willingness to continue with the trial or the future health of the primary caregiver or child. Any of the following can determine if study personnel should contact participants: the IRB, the medical monitor, the DSMB, or the PIs. The person or oversight body that makes the determination will inform the DCOC, which will instruct the ECHO ISPCTN site investigators and coordinators to contact the participants consented through their site.

ECHO ISPCTN site research staff will record any contact with participants, if necessary, in the EDC and/or trial log.

Events of Special Interest

We have not identified other reportable events for this trial.

Reporting of Pregnancy

In the unlikely event that a child participant becomes pregnant, she will be withdrawn from the trial. If a participant reports pregnancy, the site will maintain contact with pregnant study participants to obtain pregnancy outcome information. Study personnel will follow the pregnant participant until delivery or until the end of pregnancy (in the case of miscarriage or pregnancy termination).

Healthy weight management clinics during pregnancy are likely different from healthy weight management clinics outside of pregnancy, and changes in the outcomes of interest (BMI and BMIaz) should be different from those of non-pregnant participants.

If an adult caregiver becomes pregnant, study personnel will exclude from analysis all data for that adult caregiver. However, we will allow the family to remain in the study, and we will use the child's data in all analyses. All nutrition and activity guidance given during either intervention is appropriate for pregnant individuals.

9.3 UNANTICIPATED PROBLEMS

Definition of Unanticipated Problems

The Office for Human Research Protections (OHRP) considers unanticipated problems involving risks to subjects or others (UPIRTSOs) any problem, event, or new information that is:

- Unanticipated or unexpected;
- Related to the research; and
- Involves new or increased risks to subjects or others.

A UPIRTSO is not necessarily an AE or an SAE. For example, a breach of confidentiality is a potential UPIRTSO that is not an AE or SAE. Events that are potential UPIRTSOs must be reported to the central IRB, typically via the DCOC, for the central IRB to determine if the event is a UPIRTSO. These potential events must also be reported to local IRBs according to local IRB policies and procedures. The information that must be included in reports of potential UPIRTSOs is provided in the contemporaneous central (UAMS) IRB policy 10.2, *Principal Investigator Responsibilities*, which is available via *http://irb.uams.edu/irb-policies/current-irb-policies/principal-investigator-responsibilities/*

The trial-specific MOP provides additional details.

Unanticipated Problem Reporting

Study personnel must report potential UPIRTSOs, per this protocol, to the reviewing IRB, via the method(s) specified in the MOP, and directly to the DCOC. If the UAMS IRB determines the issue is, indeed, a UPIRTSO, the DCOC will notify persons and entities as required by UAMS IRB policies and procedures, the study-specific communication plan, and the DSMB charter. Study personnel must also report these events to their local institutions according to the rules and regulations of the local institution. The reviewing IRB will report the issue to OHRP, per the policies of the reviewing IRB. For the University of Arkansas for Medical Sciences (UAMS) IRB, which will be the reviewing IRB for sites in this trial, the required reporting times are provided in the contemporaneous version of Policy 10.2, *Principal Investigator Responsibilities*, which is available via *http://irb.uams.edu/irb-policies/current-irb-policies/principal-investigator-responsibilities/* The times, at the time of the approval of this protocol, are in the table below.

Table 6. Unanticipated Problem Reporting (per UAMS IRB policy at time of approval of this protocol)

**Unanticipated Problem**	**Required Reporting Time to UAMS IRB**
Death or life-threatening	Immediately to IRB office or IRB Chair
All other events	Within 10 days of event or notification of event if non-local

Reporting Unanticipated Problems to Participants

For reporting UPIRTSOs to participants, we will follow the same procedures as described for reporting event to participants (Section 9.2, subsection *Reporting Events to Participants*).

10 STATISTICAL CONSIDERATIONS

10.1 STATISTICAL HYPOTHESES

Primary Objective

Our primary objective is to conduct a rigorous feasibility trial of a proposed RCT (iAmHealthy behavioral intervention versus newsletter) in four clinics affiliated with ECHO ISPCTN site awardees to assess key variables (participant recruitment rate, participant retention, intervention dose, blinding) related to feasibility of a larger fully powered treatment-outcome RCT.

While this is a feasibility trial for the iAmHealthy intervention, this is an RCT of recruitment options. This trial does examine the recruitment rate, retention, dose, and blinding that will inform the development of a larger, fully powered RCT of the iAmHealthy behavioral intervention in the ECHO ISPCTN.

Objective 1. Participant Recruitment.

A) Evaluate the two proposed recruitment options to determine which are feasible for recruitment of rural participants. Hypothesis: The percent of randomized participants, among those contacted with each recruitment option, will be greater than 20%.

B) Compare the two proposed recruitment options. Hypothesis 1: Option 1 will yield a higher percent of participants randomized among those contacted than option 2. Hypothesis 2: Option 1 will require less time to full recruitment than option 2.

Objective 2. Participant Retention. Evaluate the percentage of randomized participants that remain in the study through the final measurement time point (six months) in both arms (i.e., iAmHealthy behavioral intervention and newsletter-only). Hypothesis: The study will retain greater than 75% of the randomized participants through the final measurement (six months) in each arm.

Objective 3. Dose. Evaluate the percentage of participants in the iAmHealthy behavioral intervention arm and retained through the final measurement who receive a sufficient dose (i.e., 80% [20.8 hours] of the 26 planned contact hours) of the intervention. Hypothesis: 80% of the participants randomized to the iAmHealthy behavioral intervention arm who are retained through the final measurement will receive a sufficient dose (i.e., 80% [20.8 hours] of the 26 planned contact hours) of the intervention.

Objective 4. Staff Blinding. Evaluate the agreement between the blinded assessor's estimation of participant assignment and actual participant arm assignment. Hypothesis: The study will achieve blinding in both arms, as shown by the New Blinding Index score with a 95% confidence interval that includes 0.[2,3]

Secondary Objectives

1. Obtain a preliminary estimate of effectiveness of the iAmHealthy behavioral intervention on change in child BMIaz, compared to the newsletter-only intervention, from the baseline measurement to the final measurement time point (sixth month).
2. Obtain a preliminary estimate of the effectiveness of the iAmHealthy behavioral intervention on change in child BMI, compared to the newsletter-only intervention, from the baseline measurement to the final measurement time point (sixth month).
3. Obtain a preliminary estimate of the effectiveness of the iAmHealthy behavioral intervention on change in primary caregiver BMI, compared to the newsletter-only intervention, from the baseline measurement to the final measurement time point (sixth month).

4. Obtain a preliminary estimate of the effectiveness of the iAmHealthy behavioral intervention on child nutrition (i.e., fruits, vegetables, etc.; see Section 7.2, subsection *Individual Sessions*), compared to the newsletter-only intervention, from the baseline measurement to the final measurement time point (sixth month).
5. Obtain a preliminary estimate of the effectiveness of the iAmHealthy behavioral intervention on moderate to vigorous physical activity (child), compared to the newsletter-only intervention, from the baseline measurement to the final measurement time point (sixth month).

10.2 SAMPLE SIZE DETERMINATION

We based the sample size on the primary efficacy endpoint, *percentage of participants randomized among those contacted by recruitment option (Objective 1A).* We will evaluate this for each of the two proposed recruitment options to determine the options that are feasible for recruitment of rural participants in a randomized design trial. We will hypothesize that consecutive recruitment (option 1) will yield a higher percent of participants randomized than the traditional recruitment option (option 2).

We will randomize up to 112 participants. This translates into 28 participants per clinic; participants will be 1:1 randomized to the newsletter-only and iAmHealthy intervention arms. For each option, we will use a one-sample test of proportions (one-sided) to test that the participant recruitment rate, percentage of participants randomized among those contacted, will be greater than 20%.

We will randomize up to 112 participants (28 participants per clinic) in the study. ECHO ISPCTN site coordinators will approach and contact up to 560 potential participants (280 per recruitment option) to consent up to 224 participant and randomize up to 112 total participants. A sample size of **280 contacted potential participants per recruitment option** will achieve 90% power to detect a difference (P1 to P0) of 0.0700 using a one-sided Z-test for proportions that use S(Phat) to estimate the standard deviation and has a continuity correction. The target significance level will be 0.05. The actual significance level achieved by this test will be 0.028. These results assume that the population proportion under the null hypothesis will be 0.13 and will be greater than 0.20 under the alternative hypothesis.

While we have hypothesized a recruitment rate of 0.20, we may find it to be higher during study implementation. Assuming a null hypothesis of 0.13, the table below provides the respective power achieved under a range (i.e., 0.2 to 0.6) of higher attained recruitment rates. As shown below (Table 7), we will maintain the study's power at 90% or above under the null hypothesis of 0.13 while detecting a difference of 0.07 (i.e., recruitment rate of 0.20).

Table 7. Power Calculation Table

Alpha	Randomized per method, per site	Recruitment rate	# approached per site	Total over 4 sites	Power
0.05	14	0.2	70	280	0.90
0.05	14	0.3	47	188	1.00
0.05	14	0.4	35	140	1.00
0.05	14	0.5	28	112	1.00
0.05	14	0.6	24	96	1.00

Based on the power calculation shown above, the number of contacted potential participants will be 280 for each recruitment option. We predict that there may be clinic-to-clinic variability (i.e., a small intraclass correlation [ICC = 0.01] at four clinical sites), implying that it is reasonable to assume that our typical cluster size will be up to m = 70 (280 participants/4 clinics) for each of the recruitment options. When participants within a clinic are more similar to one another than participants in other clinics are, the model will underestimate its unadjusted variance, and we will increase it to reflect the true variance accounting for the clinic-to-clinic variability. The variance inflation factor (VIF) will calculate the multiplying factor that we will need to increase the number of contacted potential participants to adjust for the clinic-to-clinic variability. The previously described variance inflation factor (VIF = 1 + (m-1)*ICC) indicates that we will inflate our variance by 1+(70-1)*0.01 = 1.69. The effective sample size for contacted potential participants is 166 (280/1.69). Assuming a reference proportion of 20% per recruitment option, we have 90% power to detect a 16-percentage point difference in arms with a two-sided test at 0.05 significance level, while factoring in VIF.[81]

For Objective 1B, we hypothesize that consecutive recruitment will be superior to the traditional recruitment option. Under the assumptions outlined above, we will have the power to detect a difference of 16 percentage points or the difference between 0.36 and 0.20 across the two recruitment options.

10.3 POPULATIONS FOR ANALYSES

Intent-to-Treat (ITT) Population. This population includes all randomized child/caregiver pairs. Analysis of the ITT population will be a primary analysis.

Per-Protocol (PP) Population. This population includes all randomized child/caregiver pairs who met all inclusion and no exclusion criteria, completed at least 50% of the assigned intervention, and had no major protocol deviations (i.e., a protocol deviation that has a major impact on the participant's rights, safety, or well-being; or the completeness, accuracy, and reliability of the study data).

We will first use the ITT population to conduct all analyses, and we will consider this the primary set of analyses on which to base inferences. We will also conduct analyses with the PP population.

10.4 STATISTICAL ANALYSES

General Approach

We will conduct all statistical analyses following the statistical principles for clinical trials, as specified in ICH Topic E9. We will describe and justify any deviations from the planned analyses in the final integrated clinical study report.

We will separately analyze each treatment arm, and we will summarize descriptive statistics (i.e., demographics, a post-trial questionnaire, and technology feasibility) for continuous data by mean and standard deviation or median and interquartile range, as appropriate. We will summarize categorical data by frequency and percent. We will investigate any outliers detected during data review, and we will define methods for handling outliers or data transformation in the SAP.

We will analyze all data using SAS v9.4 software (SAS Institute Inc., Cary, NC).

Analysis of the Primary Endpoint(s)

Objective 1A. Participant Recruitment: Individual Recruitment Options. We will separately calculate the percentage of participants randomized among those contacted with each recruitment option across

sites. For participants contacted through more than one recruitment option, these participants will be analyzed based on the option in which consent occurred. For each recruitment option, we will use a one-sample test of proportions to test that the *participant recruitment rate* (i.e., percentage of participants randomized among those contacted) will be greater than 20% (alternative hypothesis) versus 13% (null hypothesis). We will also calculate corresponding 95% confidence intervals for each method.

Objective 1B. Participant Recruitment: Comparison of Recruitment Options. We will compare the percent of participants randomized among those contacted between each recruitment option using a generalized linear mixed model (GLIMMIX) with a binomial distribution and logit link function. We will include a month and recruitment option as fixed effects and clinic as a random effect in the model. Using recruitment option 2 as the reference, we will also calculate odds ratios comparing recruitment options and their corresponding 95% confidence intervals.

Comparison of time to full recruitment across the two recruitment options: Four clinics will implement the two recruitments options (in random order). Each clinic will recruit 14 participants per recruitment option. The time to full recruitment per recruitment option for each clinic is defined as the number of days from start of recruitment for first participant to screening and then consent, at which point diet and activity monitoring begin for last participant. Thus for each clinic, we will have a paired set of time to full recruitment corresponding to the two recruitment options. The DCOC statistical team will use the Wilcoxon-signed rank test to compare the difference in time to full recruitment across the two recruitment options.

Objective 2. Participant Retention. We will separately calculate the percentage of randomized participants who remain through the final measurement for each intervention arm (iAmHealthy behavioral intervention, newsletter only). For each intervention arm, we will use a one-sample test of proportions to test if the *participant retention* is greater than 75%. We will also calculate the corresponding 95% confidence intervals for each condition.

Objective 3. Dose. We will calculate the percentage of families in the iAmHealthy behavioral intervention arm who receive a sufficient dose: at least 80% (20.8 planned contact hours) of the 26 planned contact hours. We will use a one-sample test of proportions to test if the percent of the families who receive this dose is greater than 80%. We will also calculate the corresponding 95% confidence intervals.

Objective 4. Staff Blinding. We will measure blinding by evaluating the agreement between the blinded assessor's estimation of participant assignment and actual participant assignment (e.g., iAmHealthy, newsletter only). To assess the agreement, a weighted kappa, we will conduct corresponding 95% confidence intervals and hypothesis test by using the Blinding Index, proposed by Bang et al (New Blinding Index).[2,3] Hypothesis testing will be whether the 95% confidence interval excludes zero. We can estimate the treatment-specific New Blinding Index and corresponding variance for treatment (i) by using the following formulas:

$$\widehat{new}BI_i = \left(2\hat{r}_{i|i} - 1\right) * (n_{i1} + n_{i2})/(n_{i1} + n_{I2} + n_{I3}), \text{ for } i=1,2$$

$$Var(\widehat{new}BI_i) = \left\{P_{1|i}\left(1 - P_{1|i}\right) + P_{2|i}\left(1 - P_{2|i}\right) + 2P_{1|i}P_{2|i}\right\}/n_i, \text{ for } i=1,2.$$

The numerator, $\left(2\hat{r}_{i|i} - 1\right) * (n_{i1} + n_{i2})$, of the New Blinding Index estimates the number of people who guess the treatment correctly beyond chance level. The denominator takes into account the number of assessors who thought the participants were assigned to the iAmHealthy behavioral intervention, newsletter-only, or “I don't know”. We will implement the calculations separately to answer the questions: 1) Was the treatment arm blinded? and 2) Was the placebo arm blinded? The

following hypotheses will be tested: $H_0: P_{1|1} = P_{2|1}$ versus $H_A: P_{1|1} > P_{2|1}$ for the treatment arm and $H_0: P_{2|2} = P_{1|2}$ versus $H_0: P_{2|2} > P_{1|2}$ for the placebo arm, where $P_{J|i}$ (*i,j*=1,2) is the "conditional" probability, and we can estimate it by $P_{j|i} = {}^{n_{ij}}/_{n_{i\cdot}}$. $P_{j|i}$=P(guess j|assigned treatment i) for i=1 (iAmHealthy, newsletter-only) And j=1 (iAmHealthy), 2(newsletter), 3 (I don't know). We will conduct this test by using the normal approximation to the binomial distribution. The New Blinding Index takes on a value between -1 and 1, where a null value of 0 indicates the most desirable situation under successful blinding. A positive value implies failure in masking above random accounting (i.e., majority of participants correctly guess child/caregiver pairs treatment allocation), and a negative value suggests success of masking or failure of masking in the other direction (i.e., more individuals mistakenly name the alternative treatment).

Analysis of the Secondary Endpoint(s)

1. To assess the effectiveness of the iAmHealthy behavioral intervention on change in **child BMIaz**, compared to the newsletter-only intervention, at six months from the baseline measurement, we will use an ANCOVA model. We will include baseline child BMIaz as a covariate in the model. For each intervention type (newsletter + iAmHealthy behavioral, newsletter only), we will also calculate corresponding 95% confidence intervals.

2. To assess the effectiveness of the iAmHealthy behavioral intervention on change in **child BMI**, compared to the newsletter-only intervention, at six months from the baseline measurement, we will use an ANCOVA model. We will include baseline child BMI as a covariate in the model. For each intervention type (newsletter + iAmHealthy behavioral, newsletter only), we will also calculate corresponding 95% confidence intervals.

3. To assess the effectiveness of the iAmHealthy behavioral intervention on change in primary **caregiver BMI**, compared to the newsletter-only intervention, at six months from the baseline measurement, we will use an ANCOVA model. We will include baseline primary caregiver BMI as a covariate in the model. For each intervention type (newsletter + iAmHealthy, newsletter only), we will also calculate corresponding 95% confidence intervals.

4. To measure the effectiveness of the iAmHealthy behavioral intervention on change in **child nutrition** (i.e., fruits, vegetables, etc.; see Section 9.1, subsection *Secondary Outcome Measures*), compared to the newsletter-only intervention, at six months from the baseline measurement, we will use an ANCOVA model. We will use baseline child nutrition as a covariate in the model. For each intervention type (newsletter + iAmHealthy behavioral, newsletter only), we will calculate corresponding 95% confidence intervals.

5. To measure the effectiveness of the iAmHealthy behavioral intervention on **moderate to vigorous physical activity (child)** compared to the newsletter-only intervention at six months from the baseline measurement as measured by the change in the number of minutes of moderate to vigorous physical activity, we will use an ANCOVA model. We will include baseline physical activity as a covariate in the model. For each intervention type (newsletter + iAmHealthy behavioral, newsletter only), we will calculate corresponding 95% confidence intervals.

Analysis of Tertiary Endpoint(s)

We will capture and assess the reasons that parents decline to participate in the iAmHealthy trial to see if reasons differ by recruitment option and/or by clinic (see Figure 2, Recruitment Flow Diagram).

We will capture and evaluate the number of participants randomized (numerator) and the number of potential participants approached (denominator) across the different recruitment options. Additionally, we will capture the number of participants randomized during a one-month period within each recruitment option as well as whether that number was at least eight randomized participants (the minimum randomized participants for each site and recruitment option). We will use these numbers to explore further the number of participants we will need to approach in a future full-scale study to obtain the planned number of consented participants.

Safety Analyses

We will detail all treatment-related severe AEs and SAEs experienced by the child/caregiver pair, and we will summarize these by the treatment condition. We will present summary statistics for each recruitment option as overall and by clinics.

Baseline Descriptive Statistics

We will summarize baseline and child/caregiver demographic characteristics by each intervention arm, and we will apply summary statistics for both continuous and categorical variables. For continuous measures, descriptive measures will include number of non-missing values, mean, standard deviation, minimum, and maximum. For categorical variables, descriptive statistics will include frequency counts and percentages by category.

Planned Interim Analyses

We are not planning an interim analysis for this trial.

Sub-Group Analyses

We will explore the potential moderating effects of race/ethnicity, insurance status, rurality, age, or other demographic factors on primary and exploratory endpoints.

Sensitivity Analysis

We will assess the robustness of study findings to “participants’ prior knowledge of recruitment options/research team’s previous contact” via a sensitivity analysis. We will analyze the Objective 1 endpoints by using the aforementioned methods based on a subset of participants who report contact by only one recruitment option (i.e. excluding those who report being contacted via a different recruitment option).

We will also assess the robustness of study findings to “study participants who qualified before enrollment halt but did not qualify after enrollment halt (when measured at baseline)” via a sensitivity analysis. We will analyze the Objective 1 endpoints by using the aforementioned methods based on a subset of participants who do not meet the pre-post halt qualification-discrepancy (i.e., excluding those participants who qualified before enrollment halt but did not qualify after enrollment halt).

Tabulation of Individual Participant Data

DCOC staff will provide a detailed description of child and caregiver disposition across all enrolled clinics. We will tabulate the number of consented and randomized participants by treatment arm and clinic, and we will present tabulations as counts and percentages. Additionally, we will summarize the number of child/caregiver pairs either completing or discontinuing the trial by using counts and percentages.

11 SUPPORTING DOCUMENTATION AND OPERATIONAL CONSIDERATIONS

11.1 REGULATORY, ETHICAL, AND STUDY OVERSIGHT CONSIDERATIONS

Informed Consent Process

CONSENT/ASSENT AND OTHER INFORMATIONAL DOCUMENTS PROVIDED TO PARTICIPANTS

See subsection 7.1

CONSENT PROCEDURES AND DOCUMENTATION

See subsection 7.1

(7)

Study Discontinuation and Closure

The trial may also be suspended or stopped per any stopping/suspension specifications in the DSMB charter. It may also be stopped or suspended by the central IRB.

Early termination may be permanent if there is sufficient cause.

The suspending or terminating party will provide, directly or indirectly, written notification documenting the reason for trial suspension or termination to the following, as applicable: trial participants, PIs, ECHO ISPCTN site investigators, reviewing IRB, local IRBs, NIH, DCOC, and OHRP. Persons and offices notified will include those specified in the trial MOP, the reviewing IRB's policies and procedures, and the SMART IRB policies and procedures.

The suspending or terminating party will also contact trial participants and inform them of any changes to the trial visit schedule. Circumstances that may warrant termination or suspension include, but are not limited to:

- Determination of unexpected, significant, or unacceptable risk to participants
- Demonstration of efficacy that would warrant stopping
- Insufficient compliance to protocol requirements
- Data that are not sufficiently complete and/or evaluable
- Determination that the primary endpoint has been met
- Determination of futility

If the trial is temporarily suspended, it may resume once concerns about safety, protocol compliance, and data quality are addressed and are satisfactory to the DSMB, the reviewing IRB, the local IRB(s) (when applicable), the funding agency, and the sponsor (DCOC).

Confidentiality and Privacy

We will conduct all trial activities in as private a manner as possible.

Records will be maintained as required by the privacy and security rules promulgated by the Health Insurance Portability and Accountability Act (HIPAA) (Title 45 of the CFR Part 164).[82,83]

During the trial, study personnel will keep all trial records in secure locations that only authorized personnel can access. Examples of secure locations include, but are not limited to: 1) locked file cabinet(s) in a limited (badge or key) access room, or 2) password-protected computer systems. Study personnel may only transmit records that contain PHI, as defined by HIPAA, through an open email system if the personnel encrypt the data. Password protection alone is insufficient for data transmission through an open email system (e.g., Outlook). After trial completion, access to trial records will be limited (see next Section).

Certain bodies/institutions may need to review information, including the participant information, for any of the following reasons: to process information or to ensure compliance with the protocol and other applicable requirements (such as the policies and procedures of the reviewing IRB). Institutions/bodies that may have access to the participants' information include:

- UAMS IRB and other oversite offices
- IRB for the site through which the participant is consented
- OHRP
- DCOC
- NIH

Individuals with access to trial records will be:

- PIs
- Site PIs
- Site coordinators
- Data managers at participating site(s)

Future Use of Stored Specimens and Data

No specimens will be collected or stored for this trial. Regarding stored data, study personnel will document all trial interactions, and these will be password protected in secured facility/location.

The study team will place participant's de-identified data and other limited information, such as race and ethnic group, into one or more centralized database(s). The study team will share this data in compliance with the NIH data sharing policy.

For future studies using any procedures or analysis not specified in this protocol, IRB approval is required.

In the event that another investigator/collaborator has a meaningful purpose for accessing the data retrieved in this protocol, the DCOC must consult the PIs and the IRB must approve.

Key Roles and Study Governance

**Protocol Chair**	
Name, degree, title	Paul Darden, MD<br>Chief, General and Community Pediatrics
Institution	University of Oklahoma Health Sciences Center
Address	1100 N. Lindsay, Oklahoma City, OK 73104
Phone Number	(405) 271-4407
Email	paul-darden@ouhsc.edu
**Principal Investigators**	
Name, degree, title	Paul Darden, MD<br>Chief, General and Community Pediatrics
Institution	University of Oklahoma Health Sciences Center
Address	1100 N. Lindsay, Oklahoma City, OK 73104
Phone Number	(405) 271-4407
Email	paul-darden@ouhsc.edu
Name, degree, title	Ann M. Davis, PhD, MPH, ABPP<br>Ralph L. Smith Professor of Pediatrics
Institution	University of Kansas Medical Center
Address	3901 Rainbow Blvd., MS 4004, Kansas City, KS 66160
Phone Number	(913) 588-5928
Email	adavis6@kumc.edu
**Operational Principal Investigator**	
Name, degree, title	Jessica Snowden, MD<br>Associate Professor, Pediatric Infectious Diseases
Institution	University of Arkansas for Medical Sciences
Address	13 Children's Way, Slot 512-35, Little Rock, AR 72202
Phone Number	501-364-4693
Email	jsnowden@uams.edu
**Statistician**	
Name, degree, title	Milan Bimali, PhD<br>Assistant Professor, College of Public Health
Institution	University of Arkansas for Medical Sciences
Address	4301 West Markham, # 781, Little Rock, AR 72205
Phone Number	(501) 526-6724
Email	MBimali@uams.edu
**Data and Coordinating Operations Center (DCOC) PI**	
Name, degree, title	Jeannette Lee, PhD<br>Professor, College of Public Health
Institution	University of Arkansas for Medical Sciences
Address	4301 West Markham, # 820, Little Rock, AR 72205
Phone Number	(501) 526-6700
Email	jylee@uams.edu

**Medical Monitor**	
Name, degree, title	Alberta Kong, MD, MPH
Institution	University of New Mexico Health Sciences Center
Address	915 Camino De Salud NE, Albuquerque, NM 87131
Phone Number	(505) 272-5152
Email	akong@salud.unm.edu

The clinical trial outlined in this protocol is part of the ECHO ISPCTN, a branch of the Environmental influences on Child Health Outcomes (ECHO) program supported by the NIH.

The data coordination, technical instruction, data standards, quality control (QC) and quality assurance (QA), and operational coordination for the clinical trial protocol outlined here (and for the ECHO ISPCTN overall) is provided by the DCOC.

The Steering Committee governs the ECHO ISPCTN and includes representatives from all site awardees, as well as representatives from the DCOC and the NIH. Overseeing the work of the Steering Committee is the NIH ECHO office, as well as an executive Leadership Committee.

A team of content experts and DCOC staff completed this protocol, and a pediatric obesity-working group within the ECHO ISPCTN reviewed it. The NIH Protocol Review Committee and the DSMB further reviewed the protocol.

Safety Oversight

Ensuring participant safety is the responsibility of all trial team personnel, especially the PIs, ECHO ISPCTN site investigators, site coordinators, behavioral intervention team members, and monitors. A medical monitor and the DSMB will provide oversight.

The medical monitor will be a pediatrician with expertise in childhood obesity and behavioral interventions and will be independent of the trial. The NIH will convene the DSMB, and it will meet regularly, according to its charter.

The entities that will receive reports include, but are not limited to, the DCOC and the NIH. The DSMB charter will provide additional information.

Clinical Monitoring

We will conduct clinical site monitoring to ensure that study personnel are protecting the rights and well-being of trial participants, that the reported trial data are accurate, complete, and verifiable, and that the conduct of the trial complies with the currently approved protocol/amendment(s), with ICH GCP, and with applicable regulatory requirement(s).

- A DCOC team member or designee will remotely monitor site awardees to ensure data quality and integrity via weekly phone meetings and queries of EDC entries.
- On-site monitoring visits will also be performed at each site, per the Site Performance Plan, at least once during the course of the trial and if needed for cause.
- Clinical site monitors will document details of their activities and findings, per the Site Performance Plan. The Site Performance Plan describes the monitoring details (i.e., who will conduct, at what frequency, at what level of detail, and distribution of monitoring reports).

We will not conduct independent audits.

Quality Assurance and Quality Control

Each IRB-approved research site entering data will perform internal quality management of study conduct, data collection, documentation, and completion. Each site will follow the trial-specific MOP and any additional written site-specific SOPs. The operating procedures will include, but are not limited to: 1) procedures for consent; 2) data collection, entry, review, and submission processes; 3) roles and responsibilities of site personnel; and 4) training methods for study staff. The clinics and ECHO ISPCTN site awardees will provide direct access to all their facilities, source data/documents, and reports for the purpose of monitoring and auditing by the DCOC and inspection by local and regulatory authorities. When electronic health records are source data/documents, sites must provide read-only access for the monitors and auditors and anyone else authorized to inspect or verify records.

Following the DCOC monitoring SOPs, the monitors will verify that the clinical trial is conducted and that data are generated, documented (recorded), and reported in compliance with the protocol, the trial-specific Site Performance Plan, site-specific SOPs, the ICH GCP E6(R2), and applicable regulatory requirements.

We will implement QC procedures for the database and DCOC-maintained records in accordance with the Site Performance Plan, MOP, data safety monitoring plan (DSMP), and applicable SOPs. We may communicate information about any data anomalies to the site(s) for clarification/resolution.

We will address issues uncovered during QA, QC, or monitoring activities through simple corrections or root-cause analysis, followed by instituting corrective and preventative action (CAPA), as appropriate and as described in the MOP.

Data Handling and Record Keeping

DATA COLLECTION AND MANAGEMENT RESPONSIBILITIES

A formal data safety-monitoring plan will describe and document the data and workflow for the trial. The data management plan and associated documentation will specify all operations performed on data from origination to database lock, including detailed descriptions of source documentation, CRFs, instructions for completing forms, data handling and record keeping procedures, procedures for data monitoring, and reconciliation procedures and coding dictionaries to be used, if applicable. The data management plan will also describe the specific data collection and management responsibilities required of the sponsor, PIs, the site PIs, the site awardees, the clinics, and the DCOC. The data-management plan contents will be consistent with those described in the Good Clinical Data Management Practices (GCDMP). The DCOC will provide the data management plan components that document operations performed on the data to the PIs for review and approval prior to implementation.

Data collection is the responsibility of the clinical trial staff at the individual ECHO ISPCTN sites under the supervision of the site investigator. The site investigator is responsible for ensuring the accuracy, completeness, legibility, and timeliness of the data reported. All source documents must be completed using standard good documentation practices (i.e., the ALCOA-C method [attributable, legible, contemporaneous, original, accurate and complete]).

It is best practice for ECHO ISPCTN site coordinators to use hardcopies of any data recorded on paper case-report forms or trial visit worksheets/assessment forms as source document worksheets for

recording data for each participant consented in the trial. Data recorded in EDC derived from source documents must be consistent with the data recorded on the source documents.

Study personnel will enter clinical data (including demographics, physical examinations, and intervention-specific questionnaires) into an EDC system that complies with HIPAA regulations, provided by the DCOC at UAMS. The EDC system includes password protection and internal quality checks, such as automatic range checks, to identify data that appear inconsistent, incomplete, or inaccurate. Study personnel will enter clinical data directly from the source documents.

STUDY RECORDS RETENTION

Throughout the course of the trial, all site awardees and clinics will retain the source documents on site in accordance with current site-specific medical record storage procedures.

Sites must retain all trial documents in accordance with local and/or federal regulations, whichever is most stringent. Sites will not destroy any records without the written consent of the sponsor, if applicable. It is the responsibility of the sponsor to inform all investigators when these documents no longer need to be retained.

Study Deviations

Study deviations are any instances in which study personnel do not follow the study procedures as written in the protocol. Sites must record all deviations in the trial source documents. Whenever a deviation occurs, the DCOC will conduct an assessment (which they do not need to write) of the severity and risk of the deviation. Depending on the results of the assessment, the DCOC will request/ensure that there is either a corrective action or a simple one-time correction, as appropriate. A corrective action institutes a process designed to keep the specific problem from reoccurring. Typically, assessors will determine the root cause of the problem to develop the most appropriate corrective action plan.

We will provide the specific methods for handling deviations in the trial-specific MOP and/or trial-specific SOPs.

Publication and Data Sharing Policy

We will conduct this trial in accordance with the following publication and data sharing policies and regulations:

- **NIH Public Access Policy**, which ensures that the public has access to the published results of NIH-funded research. It requires scientists to submit final peer-reviewed journal manuscripts that arise from NIH funds to the digital archive PubMed Central upon acceptance for publication.
- **ECHO ISPCTN Publications and Presentations Policy**, which ensures accurate, responsible, and efficient communication of findings from ECHO ISPCTN clinical trials. The ECHO ISPCTN Steering Committee has approved and ratified the ECHO ISPCTN Publications and Presentations Policy, which includes representatives from all site awardees, as well as representatives from the NIH and the DCOC.
- **NIH Data Sharing Policy and the policy on the Dissemination of NIH-Funded Clinical Trial Information and the Clinical Trials Registration and Results Information Submission Rule.** We will register this trial at ClinicalTrials.gov, and we will submit trial results to ClinicalTrials.gov. In addition, we will make every attempt to publish results in peer-reviewed journals. Other researchers my request data from this trial by contacting Jeannette Lee, PhD, at the DCOC.

Conflict of Interest Policy

The independence of this trial from any actual or perceived influence, such as by the pharmaceutical industry, is critical. Therefore, we will disclose and manage any actual conflict of interest of persons who have a role in the design, conduct, analysis, publication, or any aspect of this trial. Furthermore, persons who have a perceived conflict of interest will be required to have such conflicts managed in a way that is appropriate to their participation in the design and conduct of this trial. The trial leadership in conjunction with the NIH ECHO office has established policies and procedures for all trial group members to disclose all conflicts of interest and will establish a mechanism for the management of all reported dualities of interest.

APPENDIX A: SAMPLE NEWSLETTER

1/2018 HC Newsletter Colds and Coughs January 2018

Find Us

healthychildren.org
Powered by pediatricians. Trusted by parents.
from the American Academy of Pediatrics

The only site and newsletter backed by 66,000 AAP pediatricians!

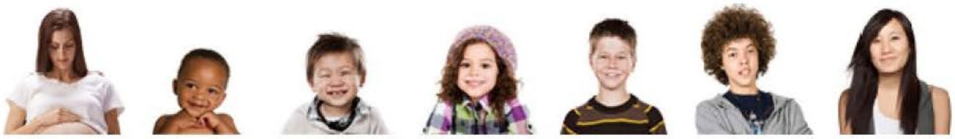

Focus on Winter Colds & Coughs

January 2018 | Issue No. 162

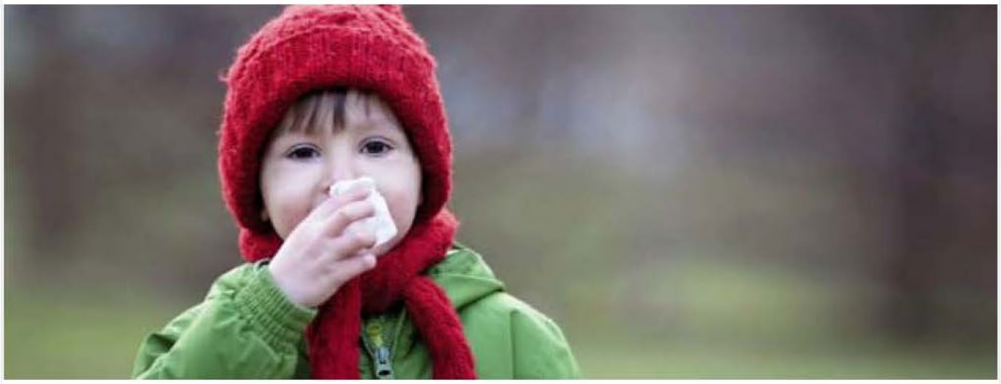

Caring for Your Child's Cold or Flu

Unfortunately, there is no cure for the common cold. Antibiotics may be used to combat bacterial infections, but they have no effect on viruses. The best you can do is to make your child comfortable.

Here are some tips from the American Academy of Pediatrics to relieve a stuffy nose and other symptoms of your child's cold.

Also In This Issue:

- **Coughs and Colds: Medicines or Home Remedies?**
- **Choosing Over-the-Counter Medicines for Your Child**
- **Why Does My Child Have a Chronic Cough?** VIDEO
- **Why Most Sore Throats, Coughs & Runny Noses Don't Need Antibiotics**

Ask the Pediatrician:

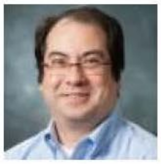

Geoffrey Simon, MD, FAAP

Why should my son get the flu shot at the pediatrician's office vs. a retail based pharmacy?

Influenza can be serious, make sure your child gets the flu vaccine this season!

:///C:/Users/adavis6/Downloads/HC_Focus-on-ColdsandCoughs_Jan2018.html

- **Preventing the Flu: Resources for Parents & Child Care Providers**
- **The Flu**
- **The Difference Between Sinusitis and a Cold**

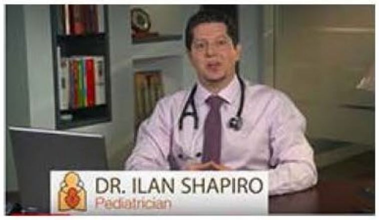

Thank You to Our Sponsors

Children's TYLENOL
Sklice (ivermectin) Lotion, 0.5%
Perrigo Nutritionals Makers of Store Brand Infant Formulas
IKEA
VICKS VapoRub

American Academy of Pediatrics
DEDICATED TO THE HEALTH OF ALL CHILDREN

The AAP is the world's largest publisher of pediatric content. HealthyChildren.org is the digital extension of the AAP mission to provide the most trustworthy health content to parents and caregivers at home, on the go, and from anywhere in the world.

The information contained on this newsletter and on HealthyChildren.org should not be used as a substitute for the medical care and advice of your pediatrician. There may be variations in treatment that your pediatrician may recommend based on individual facts and circumstances.

© Copyright 2018 American Academy of Pediatrics. All rights reserved.
American Academy of Pediatrics | 345 Park Boulevard | Itasca, IL 60143, USA

Log Into Your Account | Contact Us | About Us | Privacy Policy | Terms of Use | Editorial Policy
Unsubscribe

REFERENCES

1. Freedman DS, Berenson GS. Tracking of BMI z Scores for Severe Obesity. *Pediatrics.* 2017;140(3).
2. Bang H. Random Guess and Wishful Thinking are the Best Blinding Scenarios. *Contemp Clin Trials Commun.* 2016;3:117-121.
3. Bang H, Ni L, Davis CE. Assessment of blinding in clinical trials. *Control Clin Trials.* 2004;25(2):143-156.
4. U.S. Department of Health and Human Services. RFA-OD-16-001 Clinical Sites for the IDeA States Pediatric Clinical Trials Network. *https://grants.nih.gov/grants/guide/rfa-files/RFA-OD-16-001.html*. Published 2015. Accessed2018.
5. National Institutes of Health. Environmental Influences on Child Health Outcomes (ECHO) Program. *https://www.nih.gov/echo*. Accessed May 8, 2018.
6. Darden PM, Jacobson RM. Impact of a physician recommendation. *Human vaccines & immunotherapeutics.* 2014;10(9):2632-2635.
7. Pandolfi E, Marino MG, Carloni E, et al. The effect of physician's recommendation on seasonal influenza immunization in children with chronic diseases. *BMC public health.* 2012;12:984.
8. Ogden CL, Carroll MD, Kit BK, Flegal KM. Prevalence of childhood and adult obesity in the United States, 2011-2012. *JAMA.* 2014;311(8):806-814.
9. Johnson JA, 3rd, Johnson AM. Urban-rural differences in childhood and adolescent obesity in the United States: a systematic review and meta-analysis. *Childhood obesity.* 2015;11(3):233-241.
10. Okuboyejo S, Eyesan O. mHealth: Using Mobile Technology to Support Healthcare. *Online J Public Health Inform.* 2014;5(3):233.
11. Godino JG, Merchant G, Norman GJ, et al. Using social and mobile tools for weight loss in overweight and obese young adults (Project SMART): a 2 year, parallel-group, randomised, controlled trial. *Lancet Diabetes Endocrinol.* 2016;4(9):747-755.
12. Mummah S, Robinson TN, Mathur M, Farzinkhou S, Sutton S, Gardner CD. Effect of a mobile app intervention on vegetable consumption in overweight adults: a randomized controlled trial. *Int J Behav Nutr Phys Act.* 2017;14(1):125.
13. Hayes JF, Altman M, Coppock JH, Wilfley DE, Goldschmidt AB. Recent Updates on the Efficacy of Group Based Treatments for Pediatric Obesity. *Curr Cardiovasc Risk Rep.* 2015;9(4):16.
14. Fryar CD, Carroll MD, Ogden CL. NCHS health E-stats: Prevalence of overweight, obesity, and severe obesity among children and adolescents aged 2–19 years: United States, 1963–1965 through 2015–2016 [Pamphlet (or booklet)]. 2018. *https://stacks.cdc.gov/view/cdc/58669*.
15. Rossen LM, Talih M. Social determinants of disparities in weight among US children and adolescents. *Ann Epidemiol.* 2014;24(10):705-713 e702.
16. Freedman DS, Mei Z, Srinivasan SR, Berenson GS, Dietz WH. Cardiovascular risk factors and excess adiposity among overweight children and adolescents: the Bogalusa Heart Study. *The Journal of pediatrics.* 2007;150(1):12-17 e12.
17. U. S. Department of Health and Human Services, Health Resources and Services Administration. The Health and Well-Being of Children in Rural Areas: A Portrait of the Nation, 2011-2012. In. Rockville, Maryland.: U.S. Department of Health and Human Services,; 2015.
18. Epstein LH, Paluch RA, Roemmich JN, Beecher MD. Family-based obesity treatment, then and now: twenty-five years of pediatric obesity treatment. *Health Psychol.* 2007;26(4):381-391.

19. Davis AM, Boles RE, James RL, et al. Health behaviors and weight status among urban and rural children. *Rural Remote Health.* 2008;8(2):810.
20. Katzmarzyk PT, Barlow S, Bouchard C, et al. An evolving scientific basis for the prevention and treatment of pediatric obesity. *Int J Obes (Lond).* 2014;38(7):887-905.
21. Cloutier MM, Wiley J, Wang Z, Grant A, Gorin AA. The Early Childhood Obesity Prevention Program (ECHO): an ecologically-based intervention delivered by home visitors for newborns and their mothers. *BMC public health.* 2015;15:584.
22. Tanner A, Kim SH, Friedman DB, Foster C, Bergeron CD. Barriers to medical research participation as perceived by clinical trial investigators: communicating with rural and african american communities. *J Health Commun.* 2015;20(1):88-96.
23. Bjorn M, Brendstrup C, Karlsen S, Carlsen JE. Consecutive screening and enrollment in clinical trials: the way to representative patient samples? *J Card Fail.* 1998;4(3):225-230; discussion 231.
24. Friedman DB, Foster C, Bergeron CD, Tanner A, Kim SH. A qualitative study of recruitment barriers, motivators, and community-based strategies for increasing clinical trials participation among rural and urban populations. *Am J Health Promot.* 2015;29(5):332-338.
25. Geana M, Erba J, Krebill H, et al. Searching for cures: Inner-city and rural patients' awareness and perceptions of cancer clinical trials. *Contemporary Clinical Trials Communications.* 2017;5:72-79.
26. Parikh Y, Mason M, Williams K. Researchers' perspectives on pediatric obesity research participant recruitment. *Clinical and Translational Medicine.* 2016;5(1):20.
27. Reed RL, Barton CA, Isherwood LM, Baxter JM, Roeger L. Recruitment for a clinical trial of chronic disease self-management for older adults with multimorbidity: a successful approach within general practice. *BMC family practice.* 2013;14:125.
28. O'Connor E, Evans C, Burda B, Walsh E, Eder M, Lozano P. *Screening for Obesity and Intervention for Weight Management in Children and Adolescents: A Systematic Evidence Review for the US Preventive Services Task Force.*: Rockville, MD: Agency for Healthcare Research and Quality; June 2017 2017. Publication No. 15-05219-EF-1.
29. Maghera A, Kahlke P, Lau A, et al. You are how you recruit: a cohort and randomized controlled trial of recruitment strategies. *BMC Med Res Methodol.* 2014;14(1):111.
30. Ghai NR, Reynolds KD, Xiang AH, et al. Recruitment results among families contacted for an obesity prevention intervention: the Obesity Prevention Tailored for Health Study. *Trials.* 2014;15:463.
31. Hamilton-Shield J, Goodred J, Powell L, et al. Changing eating behaviours to treat childhood obesity in the community using Mandolean: the Community Mandolean randomised controlled trial (ComMando)--a pilot study. *Health Technol Assess.* 2014;18(47):i-xxiii, 1-75.
32. Cui Z, Seburg EM, Sherwood NE, Faith MS, Ward DS. Recruitment and retention in obesity prevention and treatment trials targeting minority or low-income children: a review of the clinical trials registration database. *Trials.* 2015;16(1):564.
33. Wilfley DE, Saelens BE, Stein RI, et al. Dose, Content, and Mediators of Family-Based Treatment for Childhood Obesity: A Multisite Randomized Clinical Trial. *JAMA Pediatr.* 2017;171(12):1151-1159.
34. Warschburger P, Kröller K. Loss to follow-up in a randomized controlled trial study for pediatric weight management (EPOC). *BMC pediatrics.* 2016;16(1):184.
35. Barlow SE. Expert committee recommendations regarding the prevention, assessment, and treatment of child and adolescent overweight and obesity: summary report. *Pediatrics.* 2007;120 Suppl 4:S164-192.
36. Epstein LH, Valoski A, Wing RR, McCurley J. Ten-year outcomes of behavioral family-based treatment for childhood obesity. *Health Psychol.* 1994;13(5):373-383.

37. Altman M, Wilfley DE. Evidence Update on the Treatment of Overweight and Obesity in Children and Adolescents. *J Clin Child Adolesc Psychol.* 2015;44(4):521-537.
38. Gartlehner G, Hansen RA, Nissman D, Lohr KN, Carey TS. Criteria for distinguishing effectiveness from efficacy trials in systematic reviews. In: *Technical Reviews.* Vol 12. Rockville (MD): Agency for Healthcare Research and Quality; 2006.
39. Waters L, St George A, Chey T, Bauman A. Weight change in control group participants in behavioural weight loss interventions: a systematic review and meta-regression study. *BMC Med Res Methodol.* 2012;12(1):120.
40. Nicholson LM, Schwirian PM, Klein EG, et al. Recruitment and retention strategies in longitudinal clinical studies with low-income populations. *Contemporary clinical trials.* 2011;32(3):353-362.
41. Implementation Science Information and Resources. National Institutes of Health Web site. Published 2017. Accessed September 28, 2017.
42. Chavez H, Ozolins D, Losek JD. Hypoglycemia and propranolol in pediatric behavioral disorders. *Pediatrics.* 1999;103(6 Pt 1):1290-1292.
43. Pence BW, Gaynes BN, Thielman NM, et al. Balancing contamination and referral bias in a randomized clinical trial: An application of pseudo-cluster randomization. *Am J Epidemiol.* 2015;182(12):1039-1046.
44. Kropski JA, Keckley PH, Jensen GL. School-based obesity prevention programs: an evidence-based review. *Obesity.* 2008;16(5):1009-1018.
45. Thompson DM, Fernald DH, Mold JW. Intraclass correlation coefficients typical of cluster-randomized studies: estimates from the Robert Wood Johnson Prescription for Health projects. *Annals of family medicine.* 2012;10(3):235-240.
46. Darden PM, Taylor JA, Slora EJ, et al. Methodological issues in determining rates of childhood immunization in office practice. A study from pediatric research in office settings (PROS). *Arch Pediatr Adolesc Med.* 1996;150(10):1027-1031.
47. Whitlock EP, Williams SB, Gold R, Smith PR, Shipman SA. Screening and interventions for childhood overweight: a summary of evidence for the US Preventive Services Task Force. *Pediatrics.* 2005;116(1):e125-144.
48. Whitlock EP, O'Connor EA, Williams SB, Beil TL, Lutz KW. Effectiveness of weight management interventions in children: a targeted systematic review for the USPSTF. *Pediatrics.* 2010;125(2):e396-418.
49. O'Connor EA, Evans CV, Burda BU, Walsh ES, Eder M, Lozano P. Screening for Obesity and Intervention for Weight Management in Children and Adolescents: Evidence Report and Systematic Review for the US Preventive Services Task Force. *JAMA.* 2017;317(23):2427-2444.
50. Lemon HM, Darden PM. Measuring practice immunization rates quickly and accurately in the era of HIPAA: validation of the quick count method in practice settings. *Journal of the South Carolina Medical Association (1975).* 2008;104(6):194-197.
51. Pew Research Center. The Smartphone Difference. *http://www.pewinternet.org/2015/04/01/us-smartphone-use-in-2015/*. Published 2015. Accessed April 2, 2015.
52. United States Census Bureau. Computer and Internet Use in the United States: 2013. *www.census.gov/content/dam/Census/library/publications/2014/acs/acs-28.pdf*. Published 2014. Accessed April 2, 2015.
53. Fox S. Pew Internet: Health, Highlights of the Pew Internet Project's research related to health and health care. 2013. *http://www.pewinternet.org/Commentary/2011/November/Pew-Internet-Health.aspx*. Published December 16, 2013. Accessed January 10, 2014.

54. Linder LA, Ameringer S, Erickson J, Macpherson CF, Stegenga K, Linder W. Using an iPad in research with children and adolescents. *Journal for specialists in pediatric nursing : JSPN.* 2013;18(2):158-154.
55. Gund A, Sjoqvist BA, Wigert H, Hentz E, Lindecrantz K, Bry K. A randomized controlled study about the use of eHealth in the home health care of premature infants. *BMC Med Inform Decis Mak.* 2013;13:22.
56. Wennberg RP. Bilirubin recommendations present problems: new guidelines simplistic and untested. *Pediatrics.* 1992;89:821-822.
57. Cohen GM, Irby MB, Boles K, Jordan C, Skelton JA. Telemedicine and Pediatric Obesity Treatment: Review of the literature and lessons learned. *Clin Obes.* 2012;2(3-4):103-111.
58. Pew Research Center. Internet/Broadband Fact Sheet. *http://www.pewinternet.org/fact-sheet/internet-broadband/#*. Published 2017. Updated January 12, 2017. Accessed January 5, 2018.
59. Davis AM, Sampilo M, Gallagher KS, Landrum Y, Malone B. Treating Rural Pediatric Obesity Through Telemedicine: Outcomes From a Small Randomized Controlled Trial. *Journal of pediatric psychology.* 2013.
60. Gallagher KS, Davis AM, Malone B, Landrum Y, Black W. Treating rural pediatric obesity through telemedicine: baseline data from a randomized controlled trial. *Journal of pediatric psychology.* 2011;36(6):687-695.
61. Barlow SE, Butte NF, Hoelscher DM, Salahuddin M, Pont SJ. Peer Reviewed: Strategies to Recruit a Diverse Low-Income Population to Child Weight Management Programs From Primary Care Practices. *Prev Chronic Dis.* 2017;14.
62. Onis M. Reliability of anthropometric measurements in the WHO Multicentre Growth Reference Study. *Acta Paediatrica.* 2006;95(S450):38-46.
63. Davis AM, James RL, Boles RE, Goetz JR, Belmont J, Malone B. The use of TeleMedicine in the treatment of paediatric obesity: feasibility and acceptability. *Matern Child Nutr.* 2011;7(1):71-79.
64. Davis AM, James RL, Curtis MR, Felts SM, Daley CM. Pediatric obesity attitudes, services, and information among rural parents: a qualitative study. *Obesity (Silver Spring).* 2008;16(9):2133-2140.
65. Lazarus AA. *Behavior Therapy and Beyond.* New York: McGraw-Hill; 1971.
66. Davison KK, Birch LL. Childhood overweight: a contextual model and recommendations for future research. *Obes Rev.* 2001;2(3):159-171.
67. Birch LL, Ventura AK. Preventing childhood obesity: what works? *Int J Obes (Lond).* 2009;33 Suppl 1:S74-81.
68. Fialkowski MK, DeBaryshe B, Bersamin A, et al. A community engagement process identifies environmental priorities to prevent early childhood obesity: the Children's Healthy Living (CHL) program for remote underserved populations in the US Affiliated Pacific Islands, Hawaii and Alaska. *Maternal and child health journal.* 2014;18(10):2261-2274.
69. Ogden CL, Carroll MD, Kit BK, Flegal KM. Prevalence of obesity and trends in body mass index among US children and adolescents, 1999-2010. *JAMA.* 2012;307(5):483-490.
70. Daniels SR. The consequences of childhood overweight and obesity. *Future Child.* 2006;16(1):47-67.
71. Deshpande A, Khoja S, A. M, Jadad AR. *Real-Time (Synchronous) Telehealth in Primary Care: Systematic Review of Systematic Reviews.* Ottawa2008.
72. Deshpande A, Khoja S, Lorca J, McKibbon A, Rizo C, Jadad AR. *Asynchronous Telehealth: Systematic Review of Analytic Studies and Environmental Scan of Relevant Initiatives.* Ottawa2008.

73. Frank GC, Berenson GS, Schilling PE, Moore MC. Adapting the 24-hr. recall for epidemiologic studies of school children. *J Am Diet Assoc.* 1977;71(1):26-31.
74. Freedson PS, Melanson E, Sirard J. Calibration of the Computer Science and Applications, Inc. accelerometer. *Med Sci Sports Exerc.* 1998;30(5):777-781.
75. Brage S, Brage N, Wedeerkopp N, K. R. Reliability and validity of the computer science and applications accelerometer in a mechanical setting. *Meas Phys Educ Exerc Sci.* 2003;7:101-119.
76. Sirard JR, Melanson EL, Li L, Freedson PS. Field evaluation of the Computer Science and Applications, Inc. physical activity monitor. *Med Sci Sports Exerc.* 2000;32(3):695-700.
77. Hendelman D, Miller K, Baggett C, Debold E, Freedson P. Validity of accelerometry for the assessment of moderate intensity physical activity in the field. *Med Sci Sports Exerc.* 2000;32(9 Suppl):S442-449.
78. Ainsworth BE, Haskell WL, Whitt MC, et al. Compendium of physical activities: an update of activity codes and MET intensities. *Med Sci Sports Exerc.* 2000;32(9 Suppl):S498-504.
79. Masse LC, Fuemmeler BF, Anderson CB, et al. Accelerometer data reduction: a comparison of four reduction algorithms on select outcome variables. *Med Sci Sports Exerc.* 2005;37(11 Suppl):S544-554.
80. Troiano RP, Berrigan D, Dodd KW, Masse LC, Tilert T, McDowell M. Physical activity in the United States measured by accelerometer. *Med Sci Sports Exerc.* 2008;40(1):181-188.
81. Campbell MK, Mollison J, Grimshaw JM. Cluster trials in implementation research: estimation of intracluster correlation coefficients and sample size. *Stat Med.* 2001;20(3):391-399.
82. The Security Rule. Health Information Privacy Web site. Published 2017. Accessed June 12, 2017.
83. The HIPAA Privacy Rule. Health Information Privacy Web site. Published 2015. Accessed June 12, 2017.
