# Supplementary material for: Active vs Traditional Methods of Recruiting Children for a Clinical Trial in Rural Primary Care Clinics: A Cluster-Randomized Clinical Trial
Source: JAMA Netw Open. 2022 Nov 29;5(11):e2244040. doi: 10.1001/jamanetworkopen.2022.44040 (PMC9709648; doi:10.1001/jamanetworkopen.2022.44040)
Supplement: Supplement 2. — Data Sharing Statement [file jamanetwopen-e2244040-s002.pdf]

Data Sharing Statement

Darden, II. Active vs Traditional Methods of Recruiting Children for a Clinical Trial in Rural Primary Care Clinics. *JAMA Netw Open*. Published November 29, 2022.
doi:10.1001/jamanetworkopen.2022.44040

Data
Data available: Yes
Data types: Deidentified participant data, Data dictionary
How to access data: Data can be requested from askdcoc@uams.edu
When available: With publication

Supporting Documents
Document types: None

Additional Information
Who can access the data: researchers whose proposed use of the data has been approved
Types of analyses: Any purpose
Mechanisms of data availability: after approval of a proposal and a signed data access agreement
